# Multicenter Collaborative Study to Optimize Mass Spectrometry Workflows of Clinical Specimens

**DOI:** 10.1021/acs.jproteome.3c00473

**Published:** 2023-11-28

**Authors:** Oliver Kardell, Christine von Toerne, Juliane Merl-Pham, Ann-Christine König, Marcel Blindert, Teresa K. Barth, Julia Mergner, Christina Ludwig, Johanna Tüshaus, Stephan Eckert, Stephan A. Müller, Stephan Breimann, Pieter Giesbertz, Alexander M. Bernhardt, Lisa Schweizer, Vincent Albrecht, Daniel Teupser, Axel Imhof, Bernhard Kuster, Stefan F. Lichtenthaler, Matthias Mann, Jürgen Cox, Stefanie M. Hauck

**Affiliations:** † Metabolomics and Proteomics Core (MPC), Helmholtz Zentrum München,German Research Center for Environmental Health (GmbH), Munich 80939, Germany; ‡ Clinical Protein Analysis Unit (ClinZfP), Biomedical Center (BMC), Faculty of Medicine, Ludwig-Maximilians-University (LMU) Munich, Großhaderner Straße 9, Martinsried 82152, Germany; § Bavarian Center for Biomolecular Mass Spectrometry at Klinikum Rechts der Isar (BayBioMS@MRI), Technical University of Munich, Munich 80333, Germany; ∥ Bavarian Center for Biomolecular Mass Spectrometry (BayBioMS), TUM School of Life Sciences, Technical University of Munich, Freising 85354, Germany; ⊥ Chair of Proteomics and Bioanalytics, Technical University of Munich, Freising 85354, Germany; # German Center for Neurodegenerative Diseases (DZNE) Munich, DZNE, Munich 81377, Germany; ¶ Neuroproteomics, School of Medicine, Klinikum Rechts der Isar, Technical University of Munich, Munich 81675, Germany; ∇ Department of Neurology, Ludwig-Maximilians-Universität München, Munich 80539, Germany; ○ Department of Proteomics and Signal Transduction, Max-Planck Institute of Biochemistry, Martinsried 82152, Germany; ⧫ Institute of Laboratory Medicine, University Hospital, LMU Munich, Munich 81377, Germany; †† Munich Cluster for Systems Neurology (SyNergy), Munich 81377, Germany; ‡‡ Computational Systems Biochemistry Research Group, Max-Planck Institute of Biochemistry, Martinsried 82152, Germany

**Keywords:** round-robin study, clinical specimen, LC–MS, mass spectrometry, data-dependent acquisition, data-independent acquisition, R package mpwR, interlaboratory, intralaboratory, CSF, plasma

## Abstract

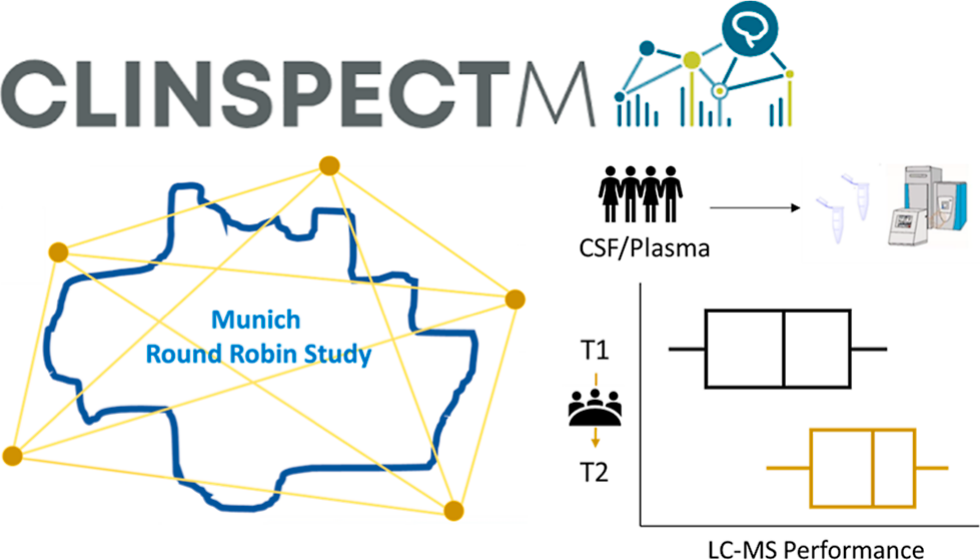

The foundation for integrating mass spectrometry (MS)-based proteomics into systems medicine is the development of standardized start-to-finish and fit-for-purpose workflows for clinical specimens. An essential step in this pursuit is to highlight the common ground in a diverse landscape of different sample preparation techniques and liquid chromatography–mass spectrometry (LC–MS) setups. With the aim to benchmark and improve the current best practices among the proteomics MS laboratories of the CLINSPECT-M consortium, we performed two consecutive round-robin studies with full freedom to operate in terms of sample preparation and MS measurements. The six study partners were provided with two clinically relevant sample matrices: plasma and cerebrospinal fluid (CSF). In the first round, each laboratory applied their current best practice protocol for the respective matrix. Based on the achieved results and following a transparent exchange of all lab-specific protocols within the consortium, each laboratory could advance their methods before measuring the same samples in the second acquisition round. Both time points are compared with respect to identifications (IDs), data completeness, and precision, as well as reproducibility. As a result, the individual performances of participating study centers were improved in the second measurement, emphasizing the effect and importance of the expert-driven exchange of best practices for direct practical improvements.

## 1 Introduction

The pursuit of gaining insights into systems medicine available for clinical utility is ongoing, and translating findings into diagnostics and therapy for various diseases are among the key objectives of mass spectrometry (MS)-based proteomics. Currently, many combinations of sample preparation techniques, instrument setups, and proteomic software tools are being explored and optimized for specific sample types and diseases to gain clinically relevant results. Imperative for a successful translation are standardized start-to-finish and fit-for-purpose workflows, which demonstrate among other key characteristics a high degree of quantitative accuracy, reproducibility, and high sample throughput.^1^ Often such fit-for-purpose workflows evolve around a specific preparation and liquid chromatography–mass spectrometry (LC–MS) setup combination. As an example, Messner et al. developed a strategy called scanning SWATH for recording precise proteomes with short gradients. The study displays the potential and clinical utility of the method by detecting prognostic plasma proteome biomarkers of COVID-19.^2^ However, the development of such standardized workflows comes at the cost of a dependency of the used analytical setup, sample preparation technique, and expertise of the laboratory. For instance, some interlaboratory studies have highlighted the importance and dependency of the operator’s expertise in generating accurate results. Highly skilled personnel are essential for each step of the workflow from sample preparation to data analysis.^3−5^

The ProteoRed Consortium performed a multicenter experiment for quality control to determine intralaboratory and interlaboratory reproducibility across multiple instrument platforms and 12 study centers. Each laboratory received both undigested and proteolyzed yeast samples, which were analyzed following a predetermined measurement protocol. Main findings showed that involved operators’ expertise had a greater impact on essential performance characteristics such as reproducibility and sensitivity than the used instrumentation.^3^

The Protein Research Group of the Association of Biomolecular Resource Facilities performed a longitudinal study to systematically evaluate the intralaboratory performance, reproducibility, and consistency over time. Over 60 participating laboratories analyzed a standard bovine protein tryptic digest mixture monthly for 9 months. While the study demonstrated that MS-based proteomics is reproducible, it also suggested that low-quality data often originated from the operator and/or the performance of the high-performance LC (HPLC) system coupled to the mass spectrometer.^4^

Furthermore, Bell et al. demonstrated in 2009 in a Human Proteome Organization (HUPO) trial study that a lack of expertise in handling the downstream data analysis can also lead to impeded results. The HUPO group distributed to 27 different laboratories an equimolar mixture of 20 highly purified recombinant human proteins for analysis. Each laboratory could perform its LC–MS method and bioinformatic analysis without constraints. Briefly, the study emphasized that differences in data analysis strategies can result in distinct protein assignments even though after standardization of the bioinformatic process all laboratories were able to detect the correct proteins.^5^ This study exemplifies the importance of bioinformatics expertise for reproducible results. While the bioinformatic pipelines and software tools have matured tremendously over the past decade, ensuring a high degree of consistency and reproducibility between different software tools, the expertise of personnel in rigorously analyzing MS data remains a crucial prerequisite in proteomic analysis. Especially for clinical studies, which generate huge data volumes, expertise in handling sophisticated pipelines is essential.^6^ Also, as the number of biomedical and translational applications in MS-based proteomics increases, new analytical challenges arise, underscoring the need for automated quality control systems. As such, Chiva et al. developed a cloud-based system, called Q-Cloud, to aid in daily quality assessment of LC–MS systems over time and to ensure that the performance is maintained at a high level, which is essential for generating reliable data.^7^

A consequent next step, going into the direction of clinical utility, is highlighting the current status quo of measuring complex clinical matrices with various standard proteomic workflows to sharpen the awareness of the benefits and limits of individual strategies as well as to gain knowledge about common tendencies. The CLINSPECT-M consortium initiated a round-robin study among its six proteomic laboratories to assess the current state of the performance of their respective workflows for measuring clinically relevant body fluids such as plasma and cerebrospinal fluid (CSF). For this purpose, the consortium partners received pooled undigested plasma and CSF samples and applied their current “best practices” for sample preparation and LC–MS measurement. No guidelines, protocols, or other restrictions were imposed to ensure the applicability of each laboratory’s workflow and to prevent any potential performance interference. After the initial evaluation of the results, workflow details including LC–MS settings and preparation protocols were shared among the laboratories, and a second round of preparation and measurements of the same samples was conducted with transferred preparation and/or LC–MS settings for fine-tuned workflows. In addition, to exclude the variable of data analysis, all generated MS data were analyzed centrally by a common pipeline using MaxQuant (7,8) as software and the R package mpwR, which offers a toolset for standardized proteomic workflow comparisons including large-scale multicenter studies.^8^ By that means, consistency in the data analysis was guaranteed, and hence, a solid foundation to compare both experimental rounds across all laboratories was provided. The various preparation and LC–MS combinations were compared in several aspects such as the number of identifications (IDs), data completeness, retention time, and quantitative precision, as well as interlaboratory reproducibility.

## 2 Experimental Procedure

### 2.1 Study Design

Pooled samples of plasma and CSF were obtained from anonymized leftover material of clinical patient diagnostics at the Institute of Laboratory Medicine, LMU Hospital, LMU Munich. The Ethics Committee of the Medical Faculty of LMU Munich has provided a waiver for the procedures involving human materials used in this study (reg. no. 23-0491 KB). Participants of the round-robin study were provided with pooled samples of plasma and CSF for an analysis with their respective best methods including preparation techniques and LC–MS settings. All partners were asked to measure ten replicates per setup and 50 ng of HeLa standard samples (Pierce HeLa, Thermo Scientific) as quality control before and after the round-robin study measurements (see Figures S1 and Figure S2). Additionally, indexed retention time (iRT) peptides (PROCAL, JPT Peptide Technologies GmbH) were spiked to the samples by the laboratories (T1:100 fmol/injection; T2:25 fmol/injection).^9^ No other restrictions were imposed on the study centers. For both T1 and T2, the same framework was used. For T2, each laboratory could adjust sample preparation and LC–MS settings considering T1 results and shared protocols and methods. An overview of changes per laboratory setup from T1 to T2 is shown in Figures S3 and S4. A detailed description of all preparation procedures and LC–MS methods is given in Sections S9–S10.

### 2.2 Software Analyses

The resulting MS files from all laboratories were collected centrally and analyzed by using a standardized data analysis pipeline. The data-dependent acquisition (DDA) data were analyzed for every measurement batch (10 technical replicates) separately with MaxQuant^10^ (v2.0.3.0) and searched against the UniProt Human Reference Proteome database (UP000005640_9606, 20,950 entries) and an iRT PROCAL sequence database. Default MaxQuant parameters were used with label-free quantification and match between runs enabled. Trypsin was specified as the enzyme, and up to two missed cleavages were allowed. Carbamidomethylation of cysteine was specified as the fixed modification, and protein N-terminal acetylation and oxidation of methionine were considered as variable modifications. The false discovery rate was set to 1% for peptide spectrum matches and the protein level. The data-independent acquisition (DIA) data were analyzed for every measurement batch separately (10 technical replicates) with MaxDIA^11^ (v2.0.3.0) with the library-based strategy. For library generation, the result file of the MaxQuant DDA search for the corresponding setup was used. Default settings for MaxDIA were used with adjustments similar to those of the DDA data analysis. For the small-scale software comparison, MaxQuant v2.4.4 and v2.7.4 were used with similar settings to MaxQuant v2.0.3.0. In addition, Spectronaut^12^ (v18.1) was used with the directDIA approach and default settings including a precursor q-value cutoff of 1% and an experiment-wise protein q-value cutoff of 1%.

### 2.3 Statistical Methods

Output files from MaxQuant were analyzed in R (v4.0.4) with the R package mpwR (https://cran.r-project.org/package=mpwR). Reversed sequences and potential contaminants only identified by site and PROCAL iRT identifications were removed prior to downstream analysis. Also, based on the achieved number of IDs across replicates, the following two outliers were removed: for CSF, T2_LabD_nanoElute_timspro_DIA_R10 and for plasma T2_LabF_ultimate_qexachf_R10. Descriptive summaries of essential performance characteristics are provided for both CSF and plasma (Tables S1 and S2). The bar plots in Figure 2 are based on these summaries. In detail, for the displayed comparisons, first, median values are determined for both T1 and T2 for a specific metric, e.g., number of protein IDs, and second, the calculated median for T1 and T2 is divided by the respective highest value of both and multiplied with 100 to get a percentage scale. Note that in Figure 2 only absolute performance indicators are included. In addition, the same principle is applied for relative performance metrics in Figure S5. These relative characteristics are calculated by dividing the absolute number by the total number and multiplying by 100, e.g., dividing the respective number of IDs for zero missed cleavages by the total number of peptides. Data completeness refers to the presence of features, such as proteins, in a specific number of technical replicates. If a feature is present in each technical run, it is considered as a full profile (100% data completeness). The data completeness in Figure 5 is calculated by only counting complete profiles. For each, the achieved number of complete profiles is divided by the total number of achieved IDs and multiplied with 100. The interlaboratory reproducibility of identifications on the protein level refers to “Majority protein IDs” of the MaxQuant’s proteinGroup.txt output file.

**Figure 1:**
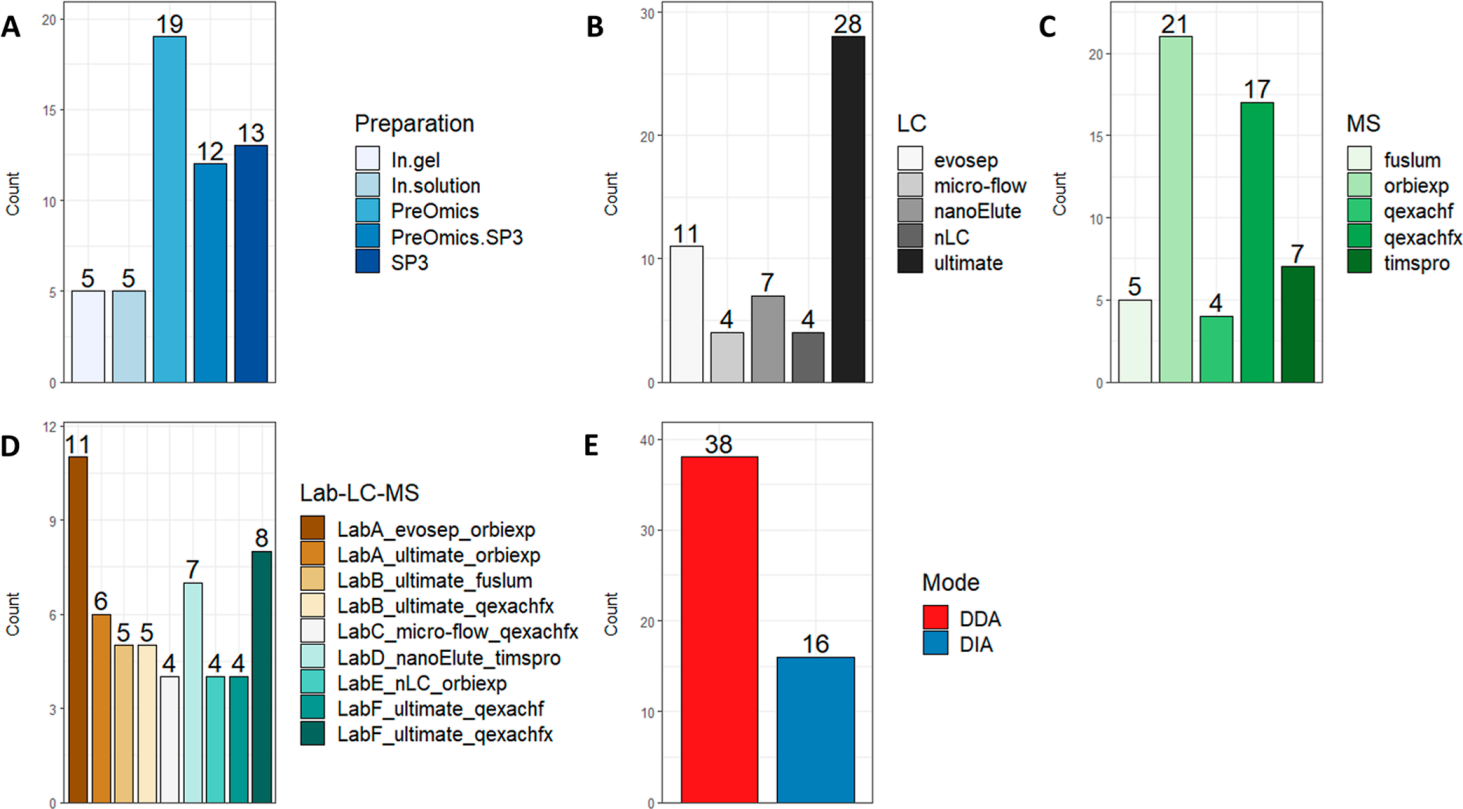
Descriptive summaries of the 54 data sets (10 replicate measurements per data set) included in this round-robin study for sample preparation procedures (A), LC systems (B), MS instruments (C), and LabID including the LC–MS setup (D) and acquisition mode (E).

**Figure 2:**
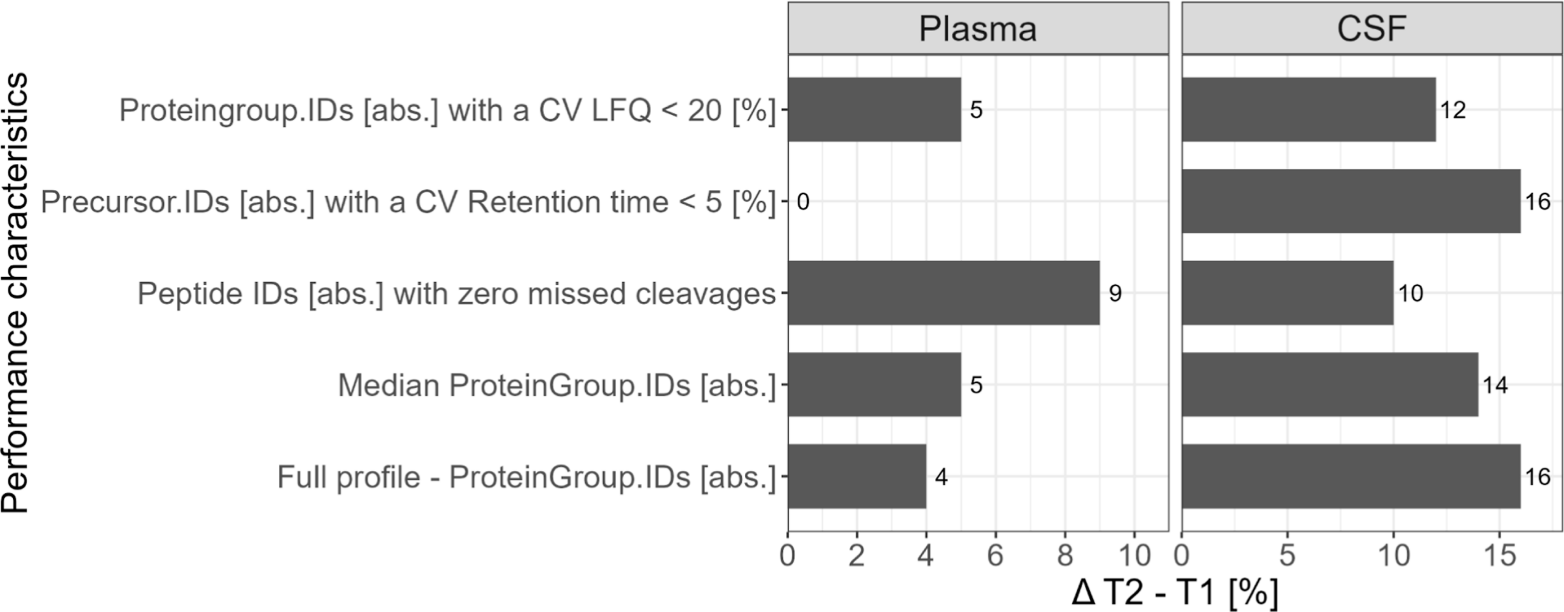
T1 vs T2—comparison of absolute characteristics for plasma and CSF by displaying difference between T2 and T1 in percentage.

**Figure 3:**
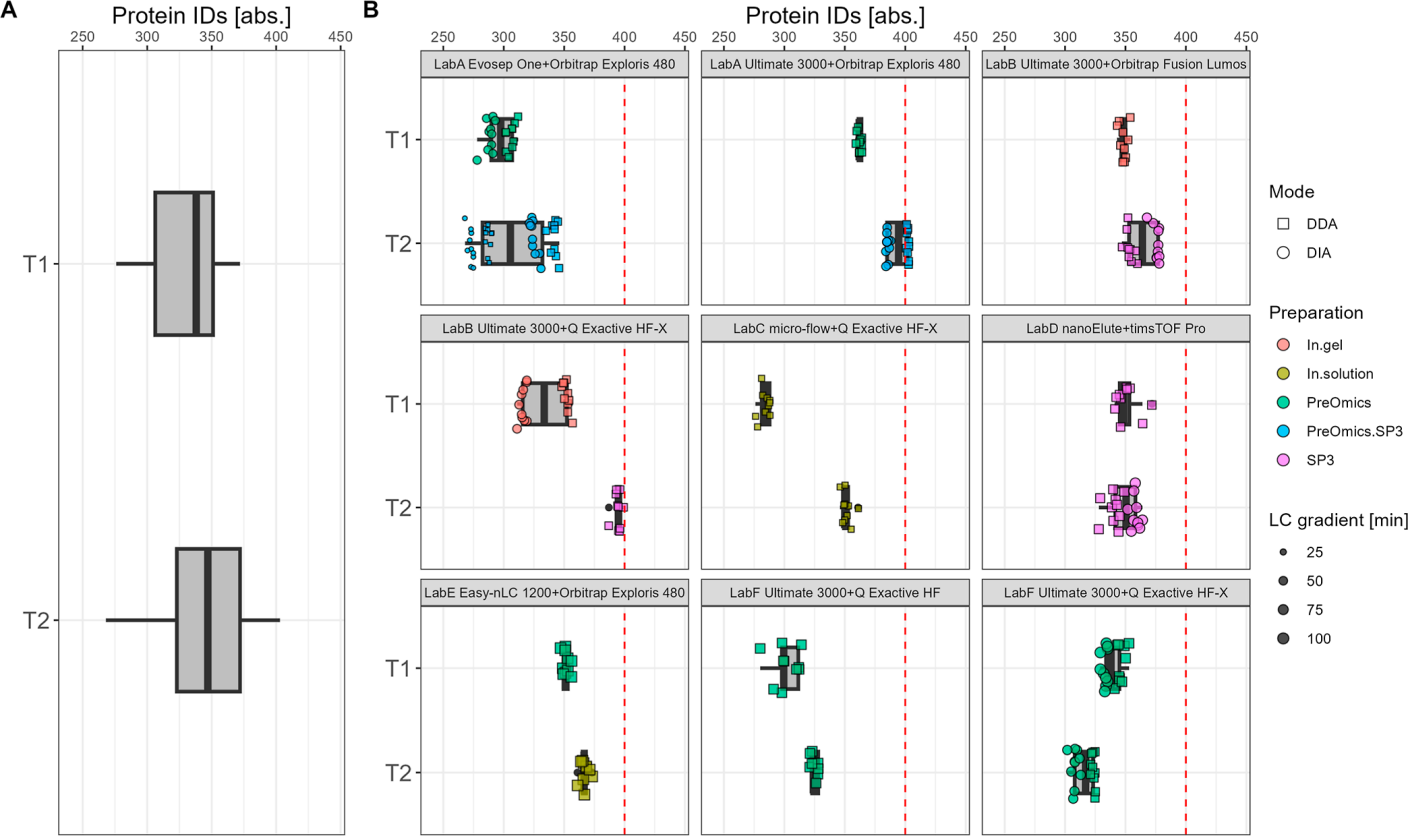
T1 vs T2—absolute number of plasma protein group IDs summarized over all data sets (A) and per specific laboratory LC–MS setup (B). The red dashed line indicates 400 protein IDs.

**Figure 4:**
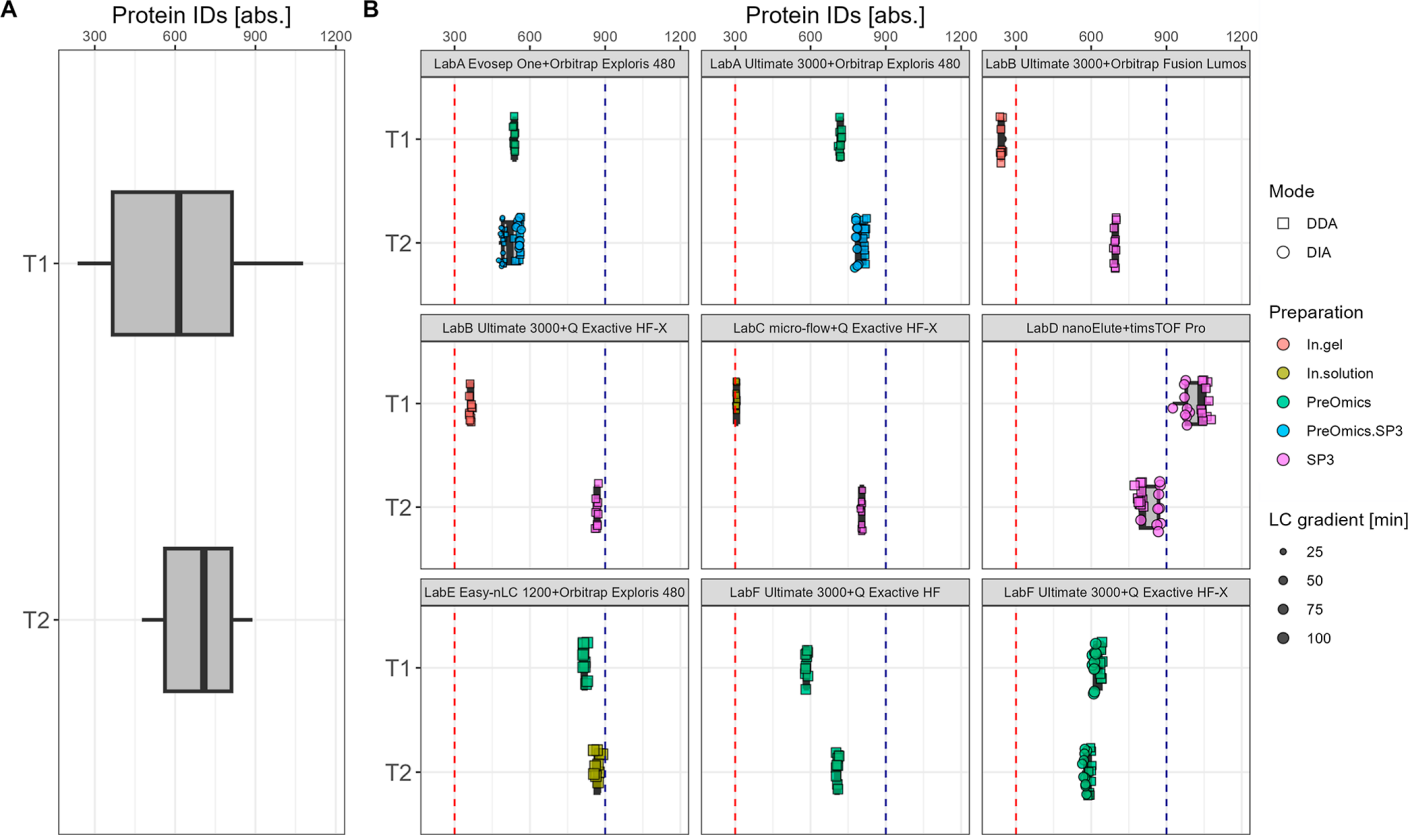
T1 vs T2—absolute number of CSF protein group IDs summarized over all data sets (A) and per specific laboratory LC–MS setup (B). The red dashed line indicates 300 protein IDs and the blue dashed line indicates 900 protein group IDs.

**Figure 5:**
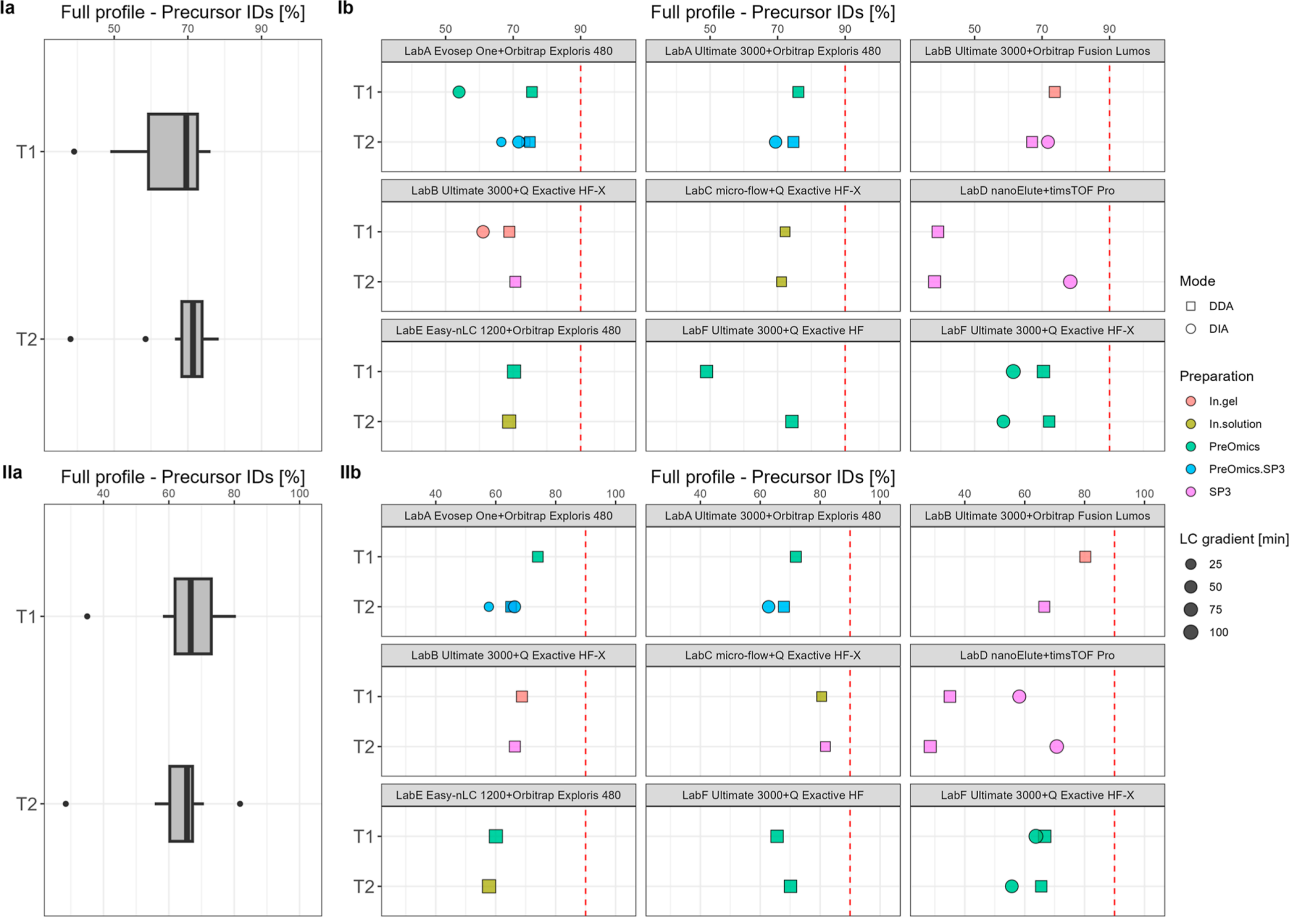
T1 vs T2—relative data completeness for complete profiles of precursor IDs [%] summarized over all data sets (a) and per specific laboratory LC–MS setup (b) for plasma (I) and CSF (II). The red dashed line indicates 90%.

### 2.4 Data Availability

The MS proteomics data have been deposited to the ProteomeXchange Consortium via the PRIDE^13^ partner repository with the data set identifier PXD044053.

## 3 Results

### 3.1 Study Design

Six study centers participated and generated a total of 54 MS data sets including two measurement rounds (T1 and T2) and two sample types (plasma and CSF). A complete list of the data sets is provided in Table S3. Among the sample preparation procedures of in-gel, in-solution, SP3,^14^ and commercially available kits from PreOmics GmbH, the PreOmics kits were most often used for sample preparation (Figure 1A). As nanoflow HPLC instruments, the UltiMate 3000 and EASY-nLC 1200 (ThermoFisher), the Evosep One (Evosep), and the nanoElute (Bruker Daltonics) were involved. Also, a microflow LC–MS system using a modified Vanquish pump (ThermoFisher) was used.^15^ For nanoflow setups, HPLC gradients varied from 21 to 120 min with an amount per injection ranging between 200 and 500 ng for both plasma and CSF samples. The measurements with the microflow system used a gradient of 30 min with an amount per injection of 5 μg. Mass spectrometer models from ThermoFisher including the Orbitrap Exploris 480, Q Exactive HF, Q Exactive HF-X, and the Orbitrap Fusion Lumos and the timsTOF Pro from Bruker Daltonics were incorporated in the study. In total, eight LC–MS setups were included, in which the UltiMate 3000 coupled to a Q Exactive HF-X setup and the Evosep One coupled to an Orbitrap Exploris 480 system contributed the most measurements (Figure 1D). In most cases, DDA was applied (Figure 1E). Note that most LC–MS setups and acquisition methods were used for both sample types. After measurement round one (T1), each laboratory could utilize the insights of the results as well as the shared knowledge in the form of preparation and workflow details to apply changes to their workflow for a second measurement round (T2) of the same samples. Importantly, each study partner could alter one or multiple aspects of the workflow. Most changes were applied in the preparation procedure and by adjusting LC settings. In some instances, no alterations of the workflows were performed. An overview of workflow changes per laboratory setup is provided in Figures S3 and S4. Detailed descriptions of the workflow adjustments are given in Sections 9–S10.

### 3.2 Performance

The performance of the workflows was compared in essential characteristics such as the number of IDs, data completeness, and quantitative and retention time precision. The first aim of the round-robin study was to determine a laboratory’s status quo, and the second was to see if sharing protocols and methods led to enhancements. The bar plots in Figure 2 demonstrate improving absolute metrics from T1 to T2, especially for CSF, with increases in identification rates between 10 and 16%. For plasma, the improvements are more subtle, ranging from 4% to the highest improvement of 9% for peptide IDs [abs] with zero missed cleavages. Only retention time precision stays constant between T1 and T2 in plasma. For relative performance indicators, no clear pattern is observable (see Figure S5). All metrics stay largely constant for T1 vs T2 (<3%). Exceptions are peptide IDs [%] with zero missed cleavages. For plasma, T2 is 11% better than T1, and for CSF, T1 is 8% ahead of T2.

A detailed view of the comparison T1 vs T2 for protein IDs is given in Figure 3 for plasma and in Figure 4 for CSF. Additionally, details for each laboratory LC–MS specific setup are highlighted with key aspects such as the preparation procedure, gradient length, and MS acquisition mode to access potential sources of trend shifts. For plasma, the range of protein IDs is between 300 and 400 across study centers for both T1 and T2. The overall trend shows a slight shift of the median number to higher values from T1 to T2. In particular, LabB and LabC contribute to the trend shift toward higher identification rates. While LabC changed from Top12 to a Top10 method and applied minor changes in the preparation procedure in T2 (see Sections S9.3 and S10.3), LabB switched from an in-gel preparation to an SP3-based procedure (see Section S10.2). At the same time, some high performers of T1 such as LabD_nanoElute_timspro lose sensitivity by altering the LC and MS settings. In detail, for the DDA measurements, a shorter column, a higher flow rate, an altered gradient, and an adjusted ion mobility window were used (see Section S9.4). For CSF, an overall shift from a median number of 613 protein IDs in T1 to a median number of 707 protein IDs in T2 is observable. In addition to increased ID rates, the variance across the study centers was reduced for CSF at T2. In T1, the protein IDs range between 300 and 1000, and in T2, they range between 400 to 900. Again, LabB and LabC achieve with their modifications in workflows and setups the biggest improvements from T1 with 300 IDs to around 900 IDs for T2, highlighting an increase of nearly 3-fold. Both study partners altered their respective sample preparation procedure toward the SP3 protocol from LabD at T1. While LabB applied no additional changes, LabC also altered MS settings (see Sections S9.2–S9.3). Furthermore, in most cases, a reduced LC gradient length correlated with a reduced number of protein IDs. As an example, in T2, for the setup LabA_evosep_orbiexp, the 60 samples per day (spd) method (T2_LabA Evosep One60spd) with a gradient length of 21 min achieves less IDs than the 30 spd method (T2_LabA Evosep One30spd) with a gradient length of 44 min at a constant input amount of 500 ng (see Figures S6A and S7A; see method details in Section S9.1). The same tendency is noticeable for LabF_ultimate_qexachfx in the comparison T1 vs T2, in which the gradient length was reduced from 95 to 60 min (see Figures S6C and S7C; see method details in Section S9.6). Additionally, details about the protein group, peptide, and precursor levels (peptide sequence including charge) are provided (see Figure S8–S11).

Another essential performance indicator was data completeness, which is shown in Figure 5 on the precursor level in percentage. The overall tendencies between T1 vs T2 for both plasma and CSF show a high similarity. The median data completeness for plasma on the precursor level revolves around 70% for both T1 and T2, and for CSF, this is around 66%. Applied changes from T1 to T2 had no significant effect on the overall relative data completeness on the precursor level. Focusing on the acquisition mode, DIA workflows range between 54 and 78% and DDA measurements range around 28–76% for both sample types, respectively. Also, for a comprehensive overview, information regarding the input amount is shown for each workflow categorized by the MS instrument (see Figures S12 and Figure S13). Further details about data completeness from the peptide level to the protein group level are displayed in Figures S14–S16.

**Figure 6:**
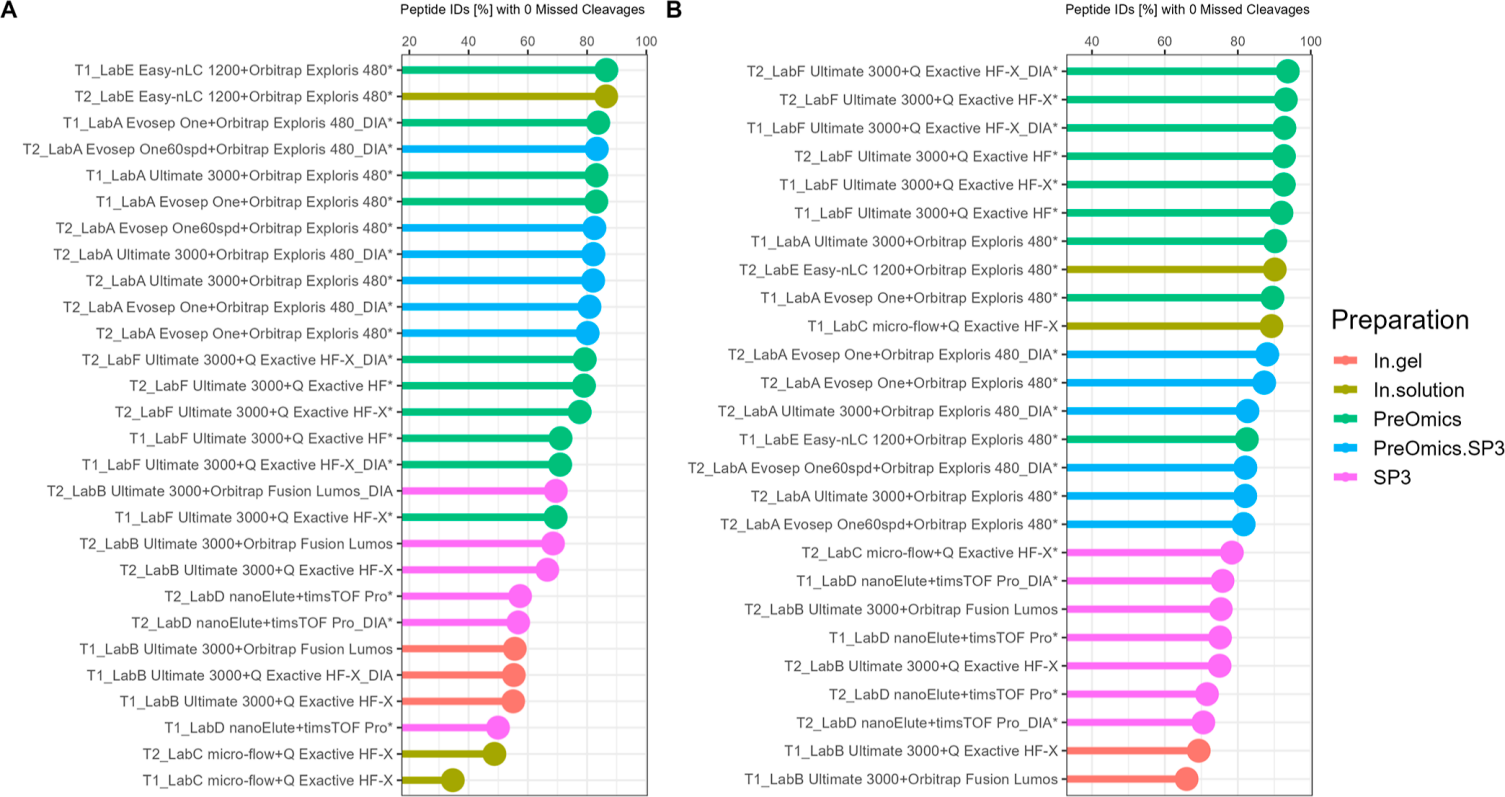
T1 vs T2—proportion of peptide IDs [%] with zero missed cleavages for plasma (A) and CSF (B). Results are color coded by the sample preparation procedure and ranked in the decreasing order. Also, workflows denoted with an asterisk used a preparation strategy including LysC-trypsin digestion, and others used trypsin digestion only.

The number of missed cleavages was an important parameter for assessing the sample preparation quality in terms of proteolysis efficiency for proteomic workflows. The number of peptide IDs [%] with zero missed cleavages is ranked in the decreasing order and colored by the preparation strategy in Figure 6. Also, workflows denoted with an asterisk used a preparation strategy including LysC-trypsin digestion, and others used trypsin digestion only. By clustering the data in this simplified way, there is a tendency where for both sample types, in most cases, in-solution approaches such as laboratory-specific protocols (In.solution) or commercially available kits (PreOmics/PreOmics.SP3) perform better than SP3 and in-gel preparation strategies. Especially for CSF samples, every in-solution-based approach achieves values with zero missed cleavages of over 80% with top performances of up to 93%. In contrast, SP3 and in-gel protocols are between 65 and 78% across workflows and laboratories. In addition, to examine potential MS instrument dependencies, the intensity distributions on the peptide level are shown for each workflow with the missed cleavage rate highlighted (see Figures S17–S26). In most cases, no trend toward lower intensity ranges is observable, and only the microflow workflow shows a tendency of detecting more peptides with missed cleavages in low abundance areas. By comparing T1 vs T2, a slight increase is observable for plasma and a minor decline is observable for CSF. For plasma, the values range between 35 and 85%, and for CSF, they range between 65 and 90% (see Figure S27).

**Figure 7:**
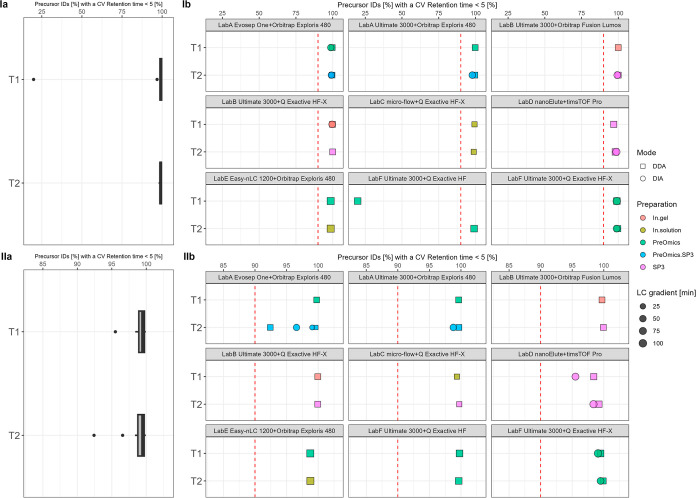
T1 vs T2—relative retention time precision of precursor IDs [%] summarized over all data sets (a) and per specific laboratory LC–MS setup (b) for plasma (I) and CSF (II). The red dashed line indicates 90%.

**Figure 8:**
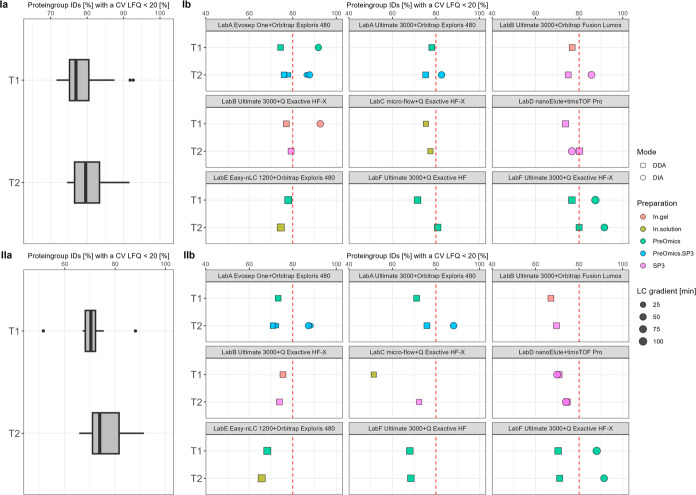
T1 vs T2—relative quantitative precision of protein group IDs [%] summarized over all data sets (a) and per specific laboratory LC–MS setup (b) for plasma (I) and CSF (II). The red dashed line indicates 80%.

Next, we focused on investigating precision of the retention time and intensity dimension by computing the coefficient of variation (CV). The percentage of precursor IDs with a retention time CV < 5% is shown in Figure 7. It is evident that each platform achieves excellent retention time precision. In almost every workflow, close to 100% of the precursor IDs have a CV under 5%. Only one workflow measurement batch is considered as a technical outlier with only 20% (Figure 7I,b, LabF Ultimate 3000+ QExactive HF). The quantitative reproducibility on the protein group level is displayed in Figure 8 by highlighting the percentage of protein group IDs with a CV LFQ <20%. Overall, a slight trend shift from a median of 76–79% for plasma and a small increase from a median of 70–73% for CSF from T1 to T2 is visible. Considering the trend of protein groups to higher ID rates (see Figure S28), not only are more protein groups detected in T2 but also these detections display a constant high quantitative precision.

### 3.3 Consistency of Protein Detections

**Figure 9:**
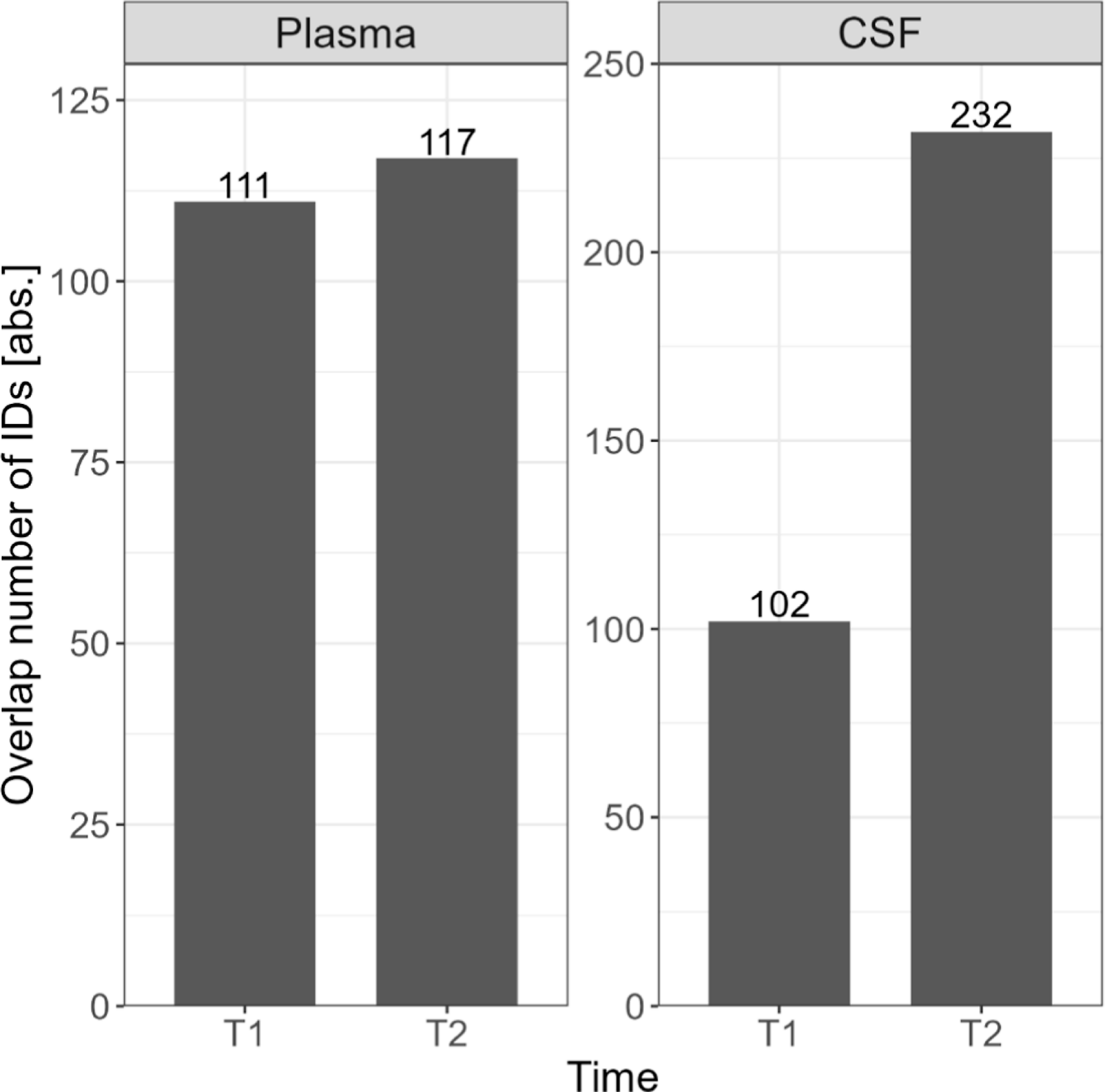
T1 vs T2—absolute number of overlapping protein IDs for plasma and CSF per measurement round (T1 and T2). Only full profiles are considered (proteins measured in every replicate per data set).

The similarity between protein detections measured with different workflows and at different sites was used as a qualitative indicator for interlaboratory reproducibility. In Figure 9, the overlap of proteins (absolute numbers) is shown for both T1 and T2 and each sample type. The results demonstrate increased reproducibility across study centers from T1 to T2. For plasma, it is only a minor enhancement from 111 proteins to 117 IDs, but for CSF, the interlaboratory reproducibility increases more than 2-fold from 102 IDs in T1 to 232 IDs in T2. Note that these numbers only consider full profiles, so these protein IDs are present in each technical replicate per setup. A closer look shows that for both sample types, as expected, the absolute overlap between IDs declines with the increasing number of setups, while the increase of the total number of IDs follows a saturation curve (see Figures S28A and S29A), resulting in a declining relative overlap (see Figures S28B and S29B). For plasma (Figure S28), the comparison between T1 and T2 of the relative overlap shows higher values for T2, even though both end points evolve around an overlap of protein IDs of 25%. This refers to the absolute overlap of 117 protein IDs for T2 and 111 protein IDs for T1 mentioned in Figure 9. For CSF (Figure S29), the increase of interlaboratory reproducibility is more evident, showing a relative overlap of 21% for T2 and only 8% for T1. The steep increase in the total number of IDs in T1 is driven by adding the results of the LabD_nanoElute_timspro workflow (Figure S29A, data sets 5 to 6), resulting in higher values for T1 over T2. Additionally, the drastic decline in the absolute overlap of IDs is attributed to the addition of the results of LabB (Figure S29B, data sets 2 to 3). Based on these extreme values, the relative overlap between laboratories for T1 is drastically reduced (see Figure S29B).

Only focusing on T2 and lowering data completeness per setup led to a growth of overlapping IDs, a considerable increase in total number of IDs, and small or no changes in the relative overlap for both sample types (see Figures S30 and S31). For instance, for plasma (Figure S30), reducing data completeness has no large effect on detecting the same proteins. A decrease to at least 80% data completeness per setup increases the absolute overlap from 117 IDs at 100% data completeness to 122 IDs for at least 80% data completeness. Simultaneously, the number of total protein IDs increases from 465 to 497 IDs, resulting in a reduced relative overlap of 24% for at least 80% data completeness in contrast to 25 for 100% data completeness.

However, by considering all data sets from T1 and T2 combined, an overlap of 18% (102 protein IDs) for plasma and an overlap of 7% (102 protein IDs) for CSF are achieved (see Figures S32A and S33A; 100% data completeness). For plasma, a total of 560 proteins are identified including all data sets, and for CSF, 1448 IDs are obtained (see Figures S32B and S33B; 100% data completeness).

### 3.4 Clinical Perspective

**Figure 10:**
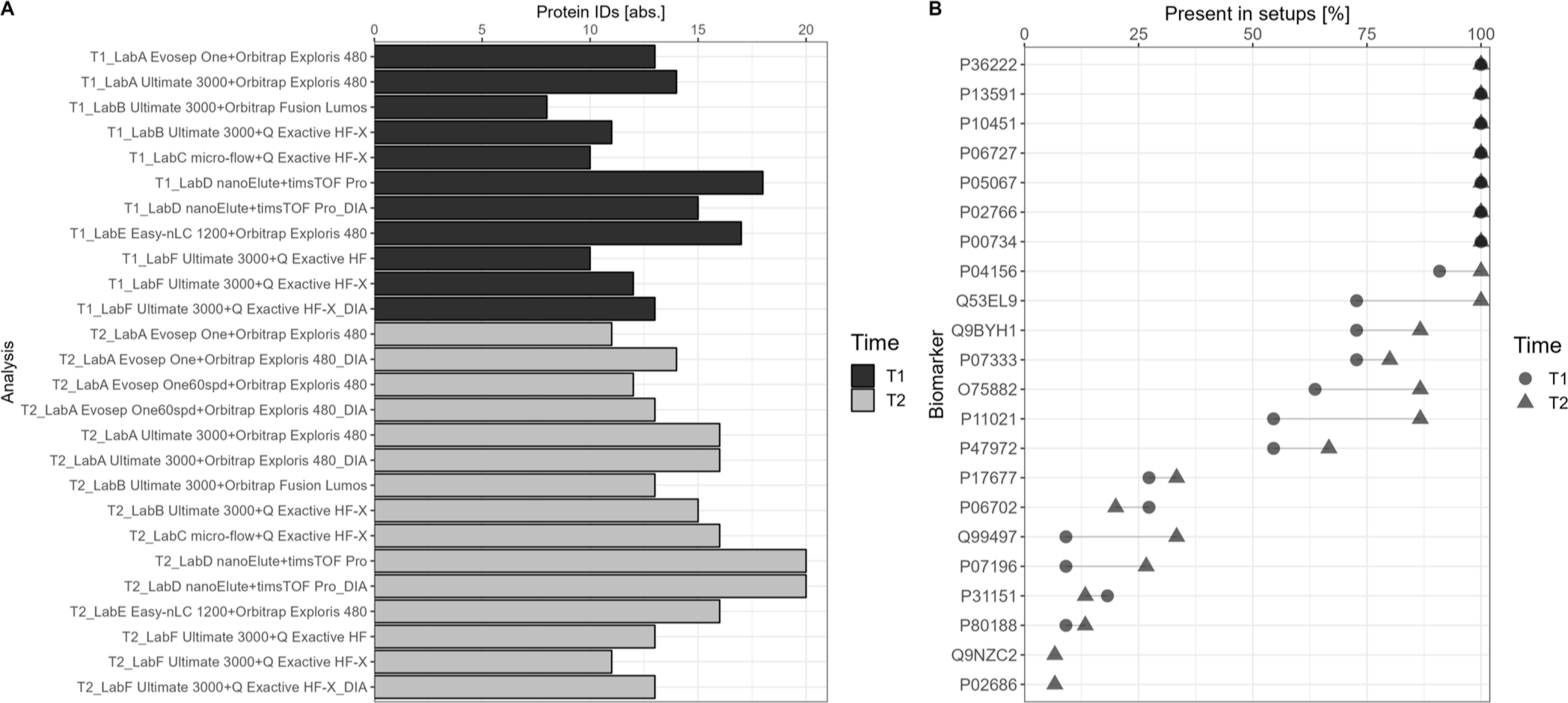
For each data set, the detected CSF proteins with full profiles are matched against 48 known CSF-related biomarkers^16^ (A). In total, 22 biomarkers are detectable across the data sets. The presence of these 22 proteins is calculated including all data sets and per respective time point (B).

In addition, clinical utility was examined by matching CSF proteins with complete profiles (100% data completeness) per setup for a set of 48 known CSF-related biomarkers^16^ and highlighting the quantitative precision of these matched IDs. Of these 48 proteins, 8 to 18 IDs are detectable in T1 in the respective setups, and in T2, the number ranges from 11 to 20 IDs (Figure 10A). The complete list of detected biomarkers is provided in Table S4. Highlighting the presence of biomarkers across all setups, in T1 seven and in T2 nine biomarkers were identified in 100% of data sets and replicates (Figure 10B). Moreover, for most proteins, there is a noticeable increase in their presence from T1 to T2. Additionally, two biomarker proteins (Q9NZC2 and P02686) are only detectable in T2 and not in any T1 setup. Focusing on the quantitative precision, most proteins show a CV LFQ of < 20% across setups and time points (Figure S34). Only in a few setups, one to two proteins display higher CV values.

## 4 Discussion

The round-robin study’s main goal was to access performance characteristics for each laboratory with its specific workflow, and the secondary objective was to assess if sharing protocols and methods resulted in improvements. By focusing on relevant clinical specimens such as plasma and CSF, the findings also emphasize individual strengths and weaknesses as well as common tendencies of the applied strategies. Since no workflow restrictions were imposed, the performed measurements reflect daily operations in the participating proteomic laboratories, and thus, any performance improvement because of the round-robin study has a direct practical benefit. Predominantly used in the study are Orbitrap instruments coupled to a variety of LC systems. In addition, the sample preparation techniques including in-gel, in-solution, SP3, and the use of standardized kits resemble a comprehensive snapshot of what is currently applied in the proteomic community. In total, the provided data serve as a valuable resource for laboratories with similar workflows and setups.

The transfer of knowledge is a key aspect of both achieving reproducible results and, if necessary, enhancing individual performances. By sharing protocols and methods within the CLINSPECT-M consortium, all absolute performance indicators improve from T1 to T2. Especially for the measurements of CSF, the performance increase is significant (Figure 2). The constant behavior of relative indicators from T1 to T2 (Figure S5) additionally points to a consistent increase for all absolute metrics. For example, not only are more protein group IDs detected but also they are consistently present in each technical replicate, reflected by the enhanced data completeness on the protein group level. In conclusion, collaborations with expertise and methods have a clear beneficial effect on the participating partners’ performance.

In addition, the round-robin study highlights common tendencies among different performance characteristics. For the plasma samples without depletion for high abundant proteins, the applied workflows and platforms achieve up to 400 protein IDs, which is in accordance with previous studies and is due to the well-known effect of high-abundance proteins masking the area of low-abundance IDs.^17−19^ For the CSF samples, the reference value for protein IDs evolves around 700 across setups. Notably, these observations are linked to the software and the analysis procedure performed here. For instance, the respective DDA runs of a particular setup were used to create an input library for the corresponding DIA analysis, and especially for DIA data, several software and library strategies are available.^12,20−22^ The use of a library with more depth or any library-free approach might improve the ID rates. Obviously, other metrics such as data completeness might also be impacted. For example, a small-scale software comparison, which was performed with the R package mpwR,^8^ between the MaxDIA pipeline and the software Spectronaut v18.1^12^ showed higher ID rates and better data completeness with the Spectronaut’s directDIA approach (see Figure S35). In any case, the data gathered from the round-robin study with a variety of preparation methods and LC–MS systems are a valuable resource for further exploring different analysis options.

Further results showed excellent retention time precision across all workflows. In addition, the monitored quantitative precision remained at a high level with around 80% of protein groups displaying a CV of < 20% across all T1 and T2 measurements.

Focusing on the missed cleavage rate, the data suggest that the used in-solution approaches achieve higher digestion efficiencies than SP3 and in-gel preparation strategies. Since the study design had many variations on sample preparation as well as LC and MS levels, no systematic and in-depth analysis for potential reasons is possible. However, for example, the study of Varnavides et al. highlights a similar tendency where iST protocols display a lower missed cleavage rate than SP3 for the analysis of HeLa samples.^23^ Also, the use of LysC-trypsin digestion is considered to be a crucial influence factor for a lower missed cleavage rate (see Figure 6). In addition, a detailed view of intensity distributions revealed no correlation between low intensity ranges and a higher missed cleavage rate. Overall, the results provide an intriguing first notion of the potential differences in the digestion efficiency with complex and clinically relevant matrices such as plasma and CSF.

A cornerstone for reliable research is not only intralaboratory reproducibility but also interlaboratory reproducibility, which was examined by focusing on data completeness as the qualitative indicator. In the case of plasma, for 100% data completeness, a presence in each technical replicate per setup, 560 proteins are identified in total by combining all data sets (T1 and T2) irrespective of being present in one or multiple setups. In contrast, considering full profiles and a presence in each setup across all laboratories, 102 plasma proteins are robustly detectable. In detail, these proteins are present in ca. 280 MS runs (minus a few outlier runs) across 28 data sets from six laboratories and are based on a variety of workflow combinations including different sample preparation techniques (in-solution, in-gel, SP3, and PreOmics kits) as well as LC systems (Evosep One, UltiMate 3000, nanoElute, Easy-nLC 1200, and microflow) and MS instruments (Q Exactive HF, Q Exactive HF-X, Fusion Lumos, Orbitrap Exploris, and timsTOF pro) measured in either the DDA or DIA mode. All of these layers of variation contribute to differences in detecting proteins. Obviously, focusing only on data sets with similar MS instruments or any other variable would potentially decrease variability and increase the overlap. Furthermore, it might be valuable to explore other software solutions and investigate the impact on interlaboratory reproducibility. However, for plasma, allowing lower data completeness levels has only a minor effect on the absolute number of overlapping IDs across study centers, while the total number of protein IDs shows a considerable increase. This indicates that enhancing intralaboratory reproducibility on the protein level by increasing data completeness does not necessarily lead to an improved interlaboratory reproducibility. In fact, it highlights the presence of workflow-specific protein signatures and raises the question of whether individual workflows can capture a greater proportion of these total 560 proteins by further improving their methods, including sample preparation strategies as well as LC–MS settings.

The overall interlaboratory reproducibility for CSF is clearly impeded by extreme values in T1. While some laboratories achieved protein IDs under 300, the top performers range around 1200 IDs. In T1, 102 proteins are present across 11 setups, reflecting an overlap of only 8%. In contrast, in T2, over 232 proteins are present in the respective 15 data sets, pointing to an overlap of over 21%. This tremendous improvement is again a clear indicator of the beneficial character of sharing expertise and knowledge, especially for samples such as CSF, which are not yet implemented in a routine manner for a broader variety of laboratories.

Highlighting CSF-related biomarkers, it is evident that a great proportion of these known biomarkers are detectable in each LC–MS workflow, while simultaneously displaying a good quantitative precision for every present protein (CV < 20% with label-free quantification). Consequently, these findings emphasize the potential of using MS-based proteomics for diagnostic analyses in clinical practice.

As a conclusion, the conducted round-robin study offered an excellent opportunity for the participating study centers to benchmark and improve their current “best practices” for relevant clinical specimens such as plasma and CSF. Not only did individual key performance indicators in each study center improve but also the interlaboratory reproducibility increased. All raw data, methods, and protocols and the standardized data analysis pipeline including the R package mpwR are accessible as valuable resources for the proteomic community.

## Supporting Information Available

QC HeLa protein group IDs before/after plasma samples; QC HeLa protein group IDs before/after CSF samples; overview about workflow changes; descriptive summary of workflow changes; comparison of relative performance characteristics; plasma protein IDs including input amount information; CSF protein IDs including input amount information; plasma protein group IDs; CSF protein group IDs; peptide IDs; precursor IDs; data completeness for plasma on the precursor level including input amount information; data completeness for CSF on the precursor level including input amount information; data completeness on the protein group level; data completeness on the protein level; data completeness on the peptide level; Log 2 median intensity distribution via stacked histograms on the peptide level for setups with an Orbitrap Exploris 480 MS instrument for plasma; Log 2 median intensity distribution via stacked histograms on the peptide level for setups with a Q Exactive HF-X MS instrument for plasma; Log 2 median intensity distribution via stacked histograms on the peptide level for setups with an Orbitrap fusion Lumos MS instrument for plasma; Log 2 median intensity distribution via stacked histograms on the peptide level for setups with a Q Exactive HF MS instrument for plasma; Log 2 median intensity distribution via stacked histograms on the peptide level for setups with a timsTOF pro MS instrument for plasma; Log 2 median intensity distribution via stacked histograms on the peptide level for setups with an Orbitrap Exploris 480 MS instrument for CSF; Log 2 median intensity distribution via stacked histograms on the peptide level for setups with a Q Exactive HF-X MS instrument for CSF; Log 2 median intensity distribution via stacked histograms on the peptide level for setups with an Orbitrap Fusion Lumos MS instrument for CSF; Log 2 median intensity distribution via stacked histograms on the peptide level for setups with a Q Exactive HF MS instrument for CSF; Log 2 median intensity distribution via stacked histograms on the peptide level for setups with a timsTOF Pro MS instrument for CSF; missed cleavages–peptide IDs with zero missed cleavages [%]; total number of protein IDs [abs.] (A) and overlapping protein IDs [%] (B) for plasma per measurement round; total number of protein IDs [abs.] (A) and overlapping protein IDs [%] (B) for CSF per measurement round; T2–overlapping protein IDs [abs.] (A), total number of protein IDs [abs.] (B) and overlapping protein IDs [%] (C) for plasma with distinct levels of data completeness [%]; T2–overlapping protein IDs [abs.] (A), total number of protein IDs [abs.] (B) and overlapping protein IDs [%] (C) for CSF with distinct levels of data completeness [%];T1 and T2–overlapping protein IDs [abs.] (A), total number of protein IDs [abs.] (B) and overlapping protein IDs [%] (C) for plasma with distinct levels of data completeness [%] T1 and T2–overlapping protein IDs [abs.] (A), total number of protein IDs [abs.] (B) and overlapping protein IDs [%] (C) for CSF with distinct levels of data completeness [%];CSF Biomarker detections and quantitative precision across workflows; software comparison for T2 LabA Evosep One + Orbitrap Exploris 480; LC–MSMS settings Lab A; LC–MSMS settings Lab B; LC–MSMS settings Lab C; LC–MSMS settings Lab D; LC–MSMS settings Lab E; LC–MSMS settings Lab F; sample preparations methods Lab A; sample preparations methods Lab B; sample preparations methods Lab C; sample preparations methods Lab D; sample preparations methods Lab E; and sample preparations methods Lab F (PDF)
Table S1: Summary of performance characteristics for CSF sample measurements (XLSX)
Table S2: Summary of performance characteristics for plasma sample measurements (XLSX)
Table S3: Summary of data sets with workflow information (XLSX)
Table S4: Detected CSF biomarker across workflows including only full profile identifications (XLSX)

## Abbreviations

The abbreviations used are CSF: cerebrospinal fluid
MS: mass spectrometry
LC: liquid chromatography
DDA: data-dependent acquisition
DIA: data-independent acquisition
IDs: identifications
MC: missed cleavages
evosep: Evosep One
ultimate: ultimate 3000
nLC: easy-nLC 1200
qexachf: Q exactive HF
qexachfx: Q exactive HF-X
fuslum: fusion lumos
orbiexp: Orbitrap exploris
timspro: timsTOF pro
T1: first measurement round of this study
T2: second measurement round of this study
