# Supplementary material for: Multicenter Collaborative Study to Optimize Mass Spectrometry Workflows of Clinical Specimens
Source: J Proteome Res. 2023 Nov 28;23(1):117–29. doi: 10.1021/acs.jproteome.3c00473 (PMC10775142; doi:10.1021/acs.jproteome.3c00473)
Supplement: Supplementary file 1 — pr3c00473_si_001.pdf [file pr3c00473_si_001.pdf]

# SUPPORTING INFORMATION FOR

# A multi-center collaborative study to optimize mass spectrometry workflows of clinical specimen

Oliver Kardell[1], Christine von Toerne[1], Juliane Merl-Pham[1], Ann-Christine König[1], Marcel Blindert[1], Teresa K. Barth[2], Julia Mergner[3], Christina Ludwig[4], Johanna Tüshaus[5], Stephan Eckert[5], Stephan A. Müller[6,7], Stephan Breimann[6,7], Pieter Giesbertz[6,7], Alexander M. Bernhardt[6,8], Lisa Schweizer[9], Vincent Albrecht[9], Daniel Teupser[10], Axel Imhof[2], Bernhard Kuster[4,5], Stefan F. Lichtenthaler[6,7,11], Matthias Mann[9], Jürgen Cox[12], Stefanie M. Hauck[1*]

[1]Metabolomics and Proteomics Core (MPC), Helmholtz Zentrum München, German Research Center for Environmental Health (GmbH), 80939, Munich, Germany;

[2]Clinical Protein Analysis Unit (ClinZfP), Biomedical Center (BMC), Faculty of Medicine, Ludwig-Maximilians-University (LMU) Munich, Großhaderner Straße 9, 82152, Martinsried, Germany;

[3]Bavarian Center for Biomolecular Mass Spectrometry at Klinikum rechts der Isar (BayBioMS@MRI), Technical University of Munich, 80333, Munich, Germany;

[4]Bavarian Center for Biomolecular Mass Spectrometry (BayBioMS), TUM School of Life Sciences, Technical University of Munich, 85354, Freising, Germany;

[5]Chair of Proteomics and Bioanalytics, Technical University of Munich, 85354, Freising, Germany;

[6]German Center for Neurodegenerative Diseases (DZNE) Munich, DZNE, 81377, Munich, Germany;

[7]Neuroproteomics, School of Medicine, Klinikum rechts der Isar, Technical University of Munich, 81675, Munich, Germany;

[8]Department of Neurology, Ludwig-Maximilians-Universität München, 80539, Munich, Germany;

[9]Department of Proteomics and Signal Transduction, Max-Planck Institute of Biochemistry, 82152, Martinsried, Germany;

[10]Institute of Laboratory Medicine, University Hospital, LMU Munich, 81377, Munich, Germany;

[11]Munich Cluster for Systems Neurology (SyNergy), 81377, Munich, Germany;

[12]Computational Systems Biochemistry Research Group, Max-Planck Institute of Biochemistry, 82152, Martinsried, Germany

*For correspondence: Stefanie M. Hauck, stefanie.hauck@helmholtz-munich.de

# Contents

1. HeLa Measurements.....5
1.1 Plasma.....5
1.2 CSF.....6
2. Applied Changes - T1 vs. T2.....7
3. Identifications.....10
3.1 Protein-level.....10
3.2 Protein group-level.....12
3.3 Peptide-level.....14
3.4 Precursor-level.....15
4. Data Completeness - relative.....16
4.1 Precursor-level.....16
4.2 Protein group-level.....18
4.3 Protein-level.....19
4.4 Peptide-level.....20
5. Intensity Distributions – Missed Cleavages.....21
5.1 Plasma.....21
5.2 CSF.....26
6. Missed Cleavages.....31
7. Inter-laboratory reproducibility.....32
7.1 T1 vs T2: Plasma.....32
7.2 T1 vs T2: CSF.....33
7.3 T2: Plasma – interlaboratory-reproducibility depending on data completeness.....34
7.4 T2: CSF - interlaboratory-reproducibility depending on data completeness.....35
7.5 T1 and T2 combined: Plasma – interlaboratory-reproducibility depending on data completeness 36
7.6 T1 and T2 combined: CSF – interlaboratory-reproducibility depending on data completeness.....37
8. Miscellaneous.....38
9. LC-MS/MS settings.....40
9.1 Laboratory A.....40
9.2 Laboratory B.....43
9.3 Laboratory C.....47

9.4 Laboratory D ........................................................48
9.5 Laboratory E ........................................................49
9.6 Laboratory F ........................................................50
10. Sample Preparation ........................................................52
10.1 Laboratory A ........................................................52
10.2 Laboratory B ........................................................53
10.3 Laboratory C ........................................................55
10.4 Laboratory D ........................................................56
10.5 Laboratory E ........................................................57
10.6 Laboratory F ........................................................59

Table S1. Summary of performance characteristics for CSF sample measurements.

Table S2. Summary of performance characteristics for plasma sample measurements.

Table S3. Summary of datasets with workflow information.

Table S4. Detected CSF biomarker across workflows including only full profile identifications.

# 1. **HeLa Measurements**

## **1.1 Plasma**

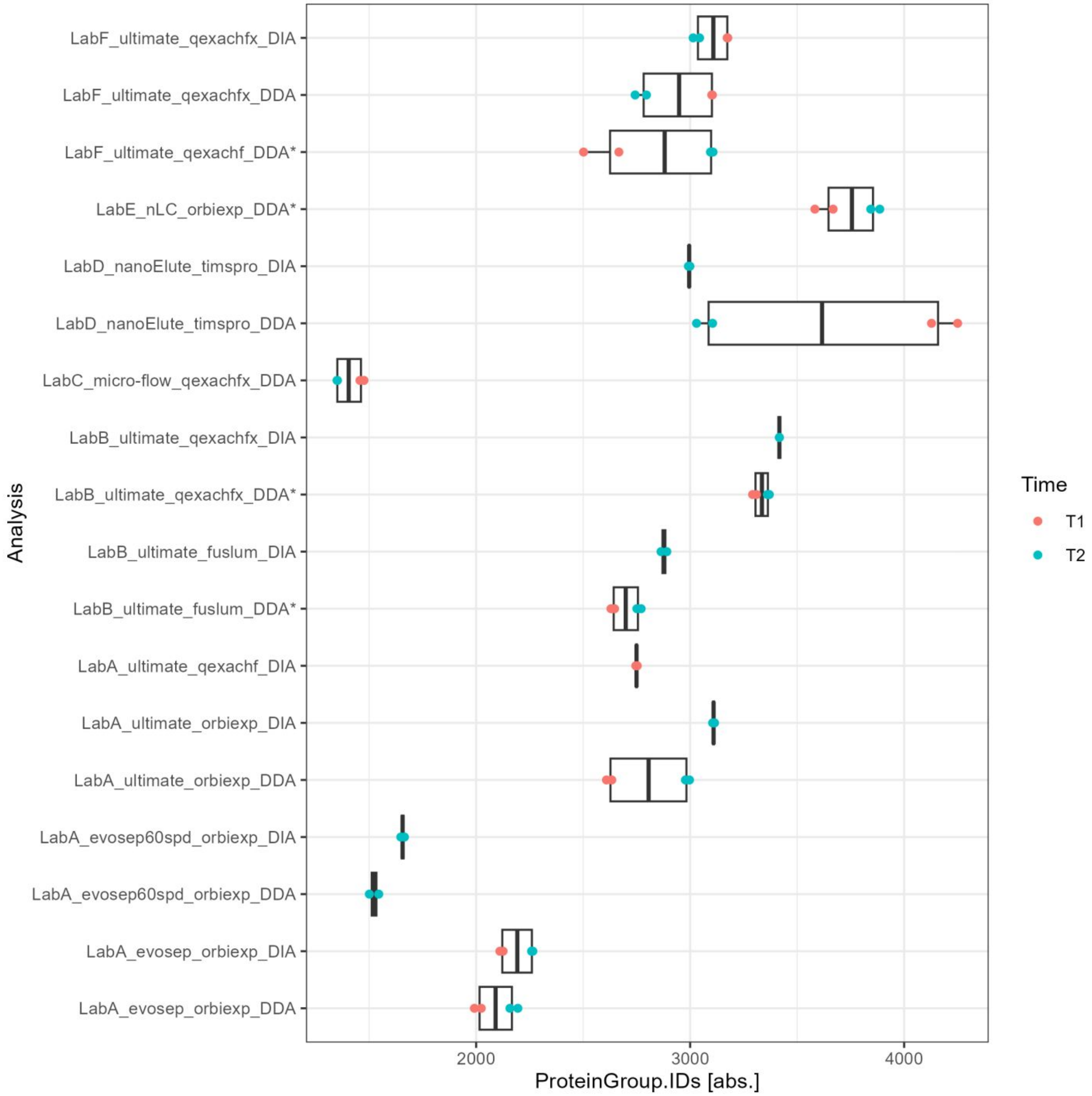


**Fig. S1: HeLa protein group IDs per analysis setup before/after plasma sample measurements at different time points, respectively.** Time point is color coded. The asterisk in setup name displays workflows where no LC/MS setting change was applied from T1 to T2 (see details in Fig. S1).

## 1.2 CSF

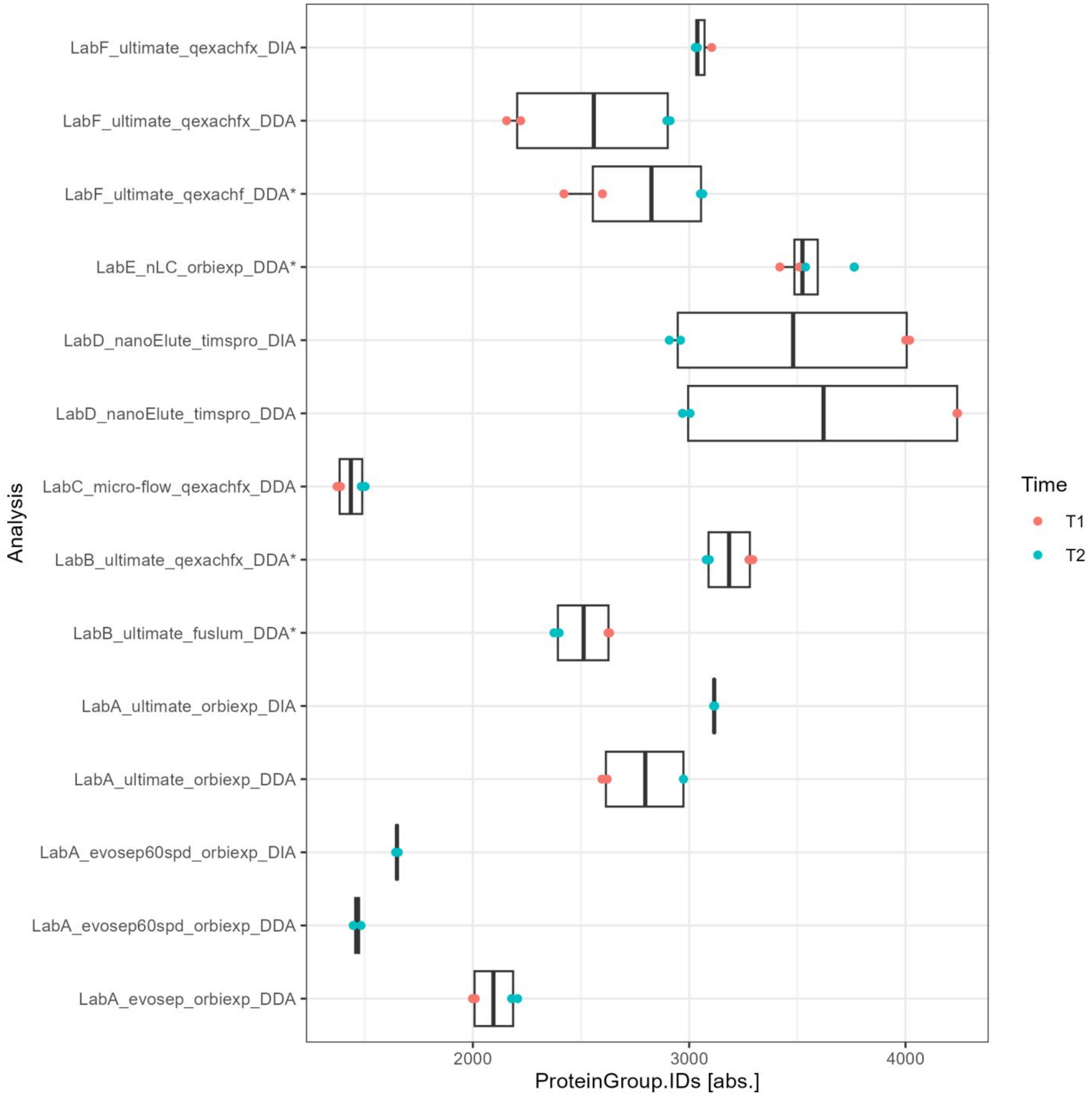


**Fig. S2: HeLa protein group IDs per analysis setup before/after CSF sample measurements at different time points, respectively.** Time point is color coded. The asterisk in setup name displays workflows where no LC/MS setting change was applied from T1 to T2 (see details in Fig. S1).

2. **Applied Changes - T1 vs. T2**

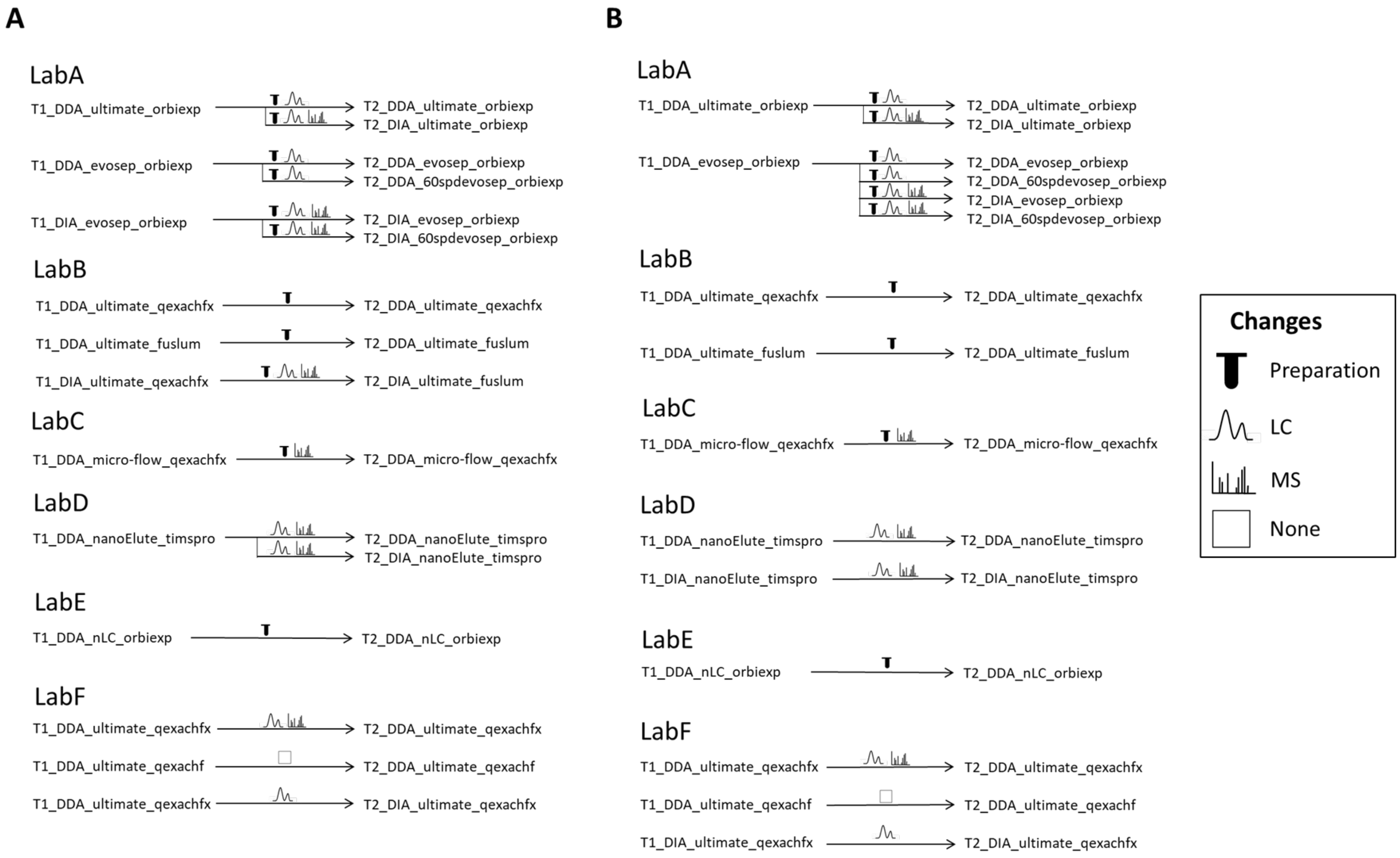


**Fig. S3:** Overview about LC-MS setups and applied workflow changes between T1 and T2 for plasma (A) and CSF (B).

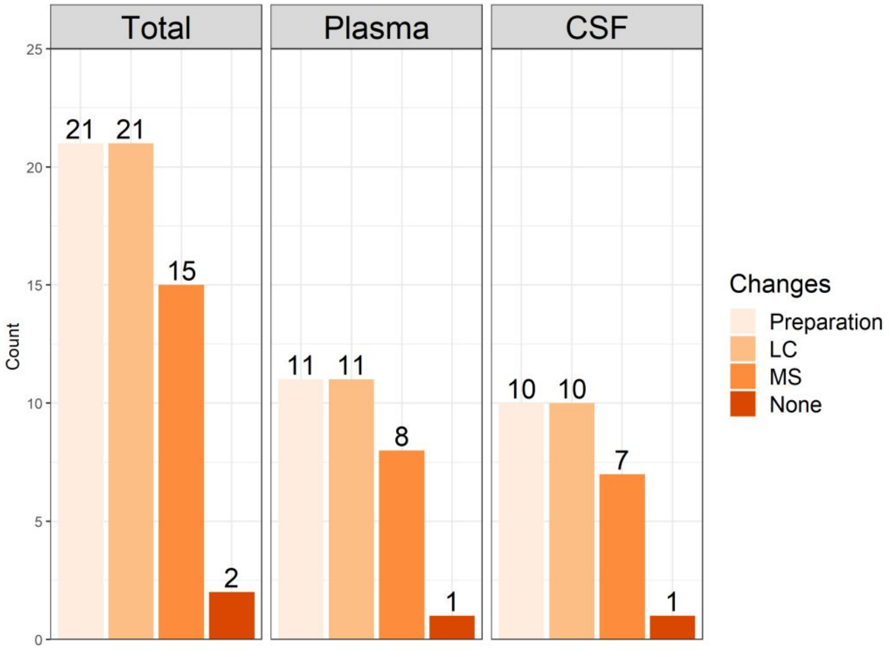


**Fig. S4:** Descriptive summaries of applied workflow changes between T1 and T2 for both samples combined, and for plasma and CSF, respectively.

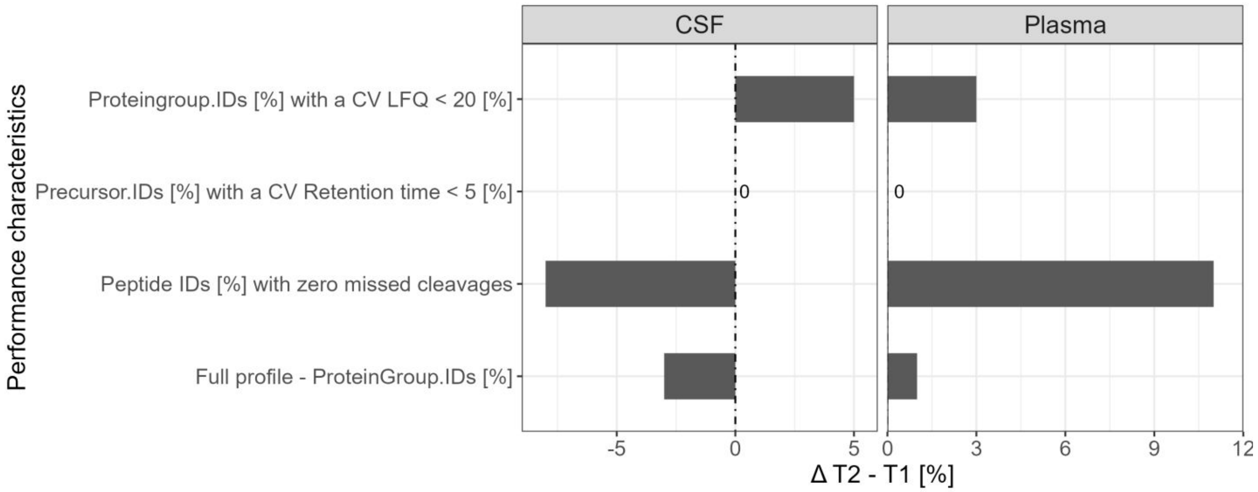


**Fig. S5:** T1 vs. T2 – Comparison of relative characteristics for plasma and CSF by displaying difference between T2 and T1 in percentage.

# 3. Identifications

## 3.1 Protein-level

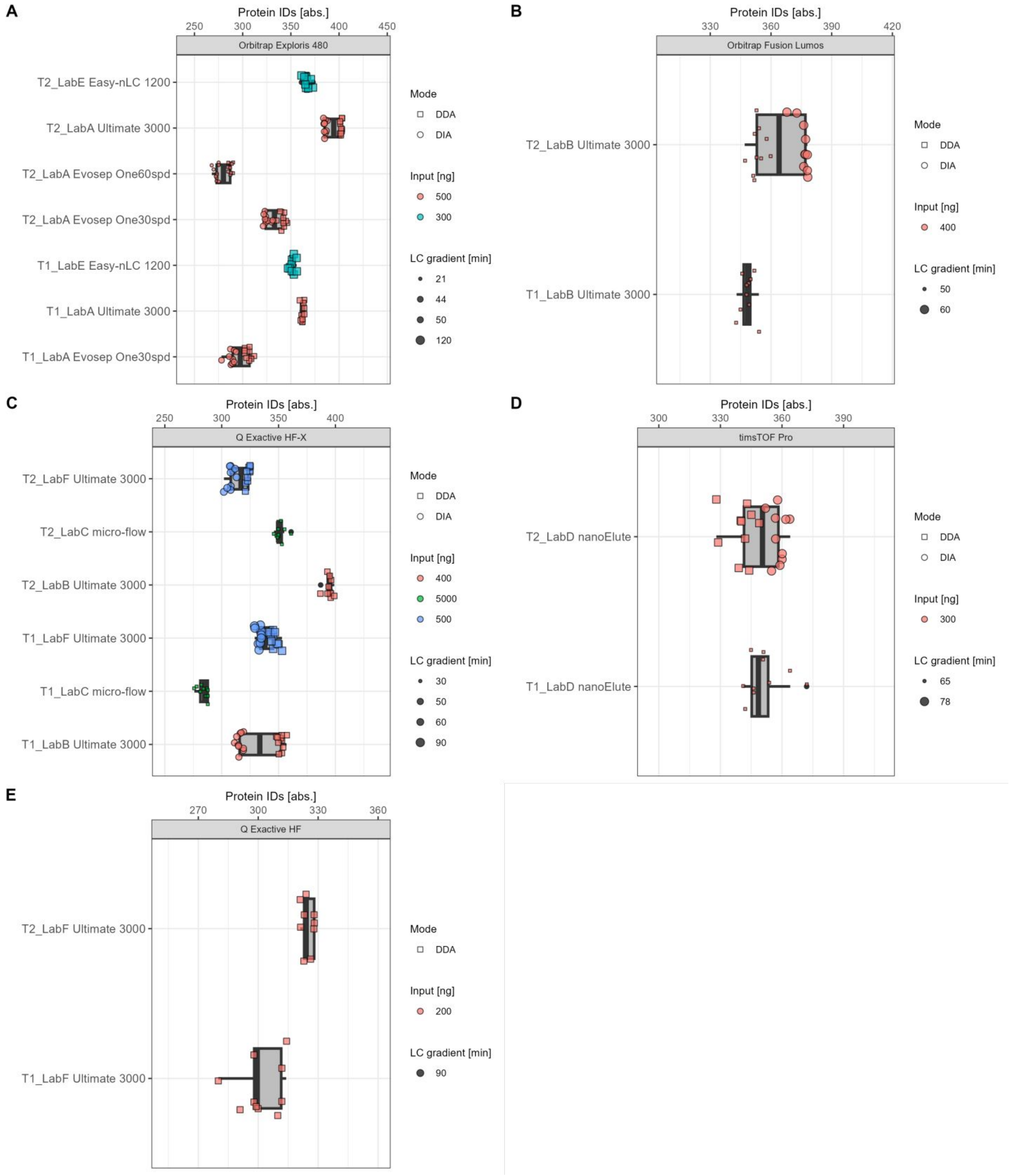


**Fig. S6:** Absolute number of plasma protein IDs for each workflow separated by different MS instruments with color coded input amount.

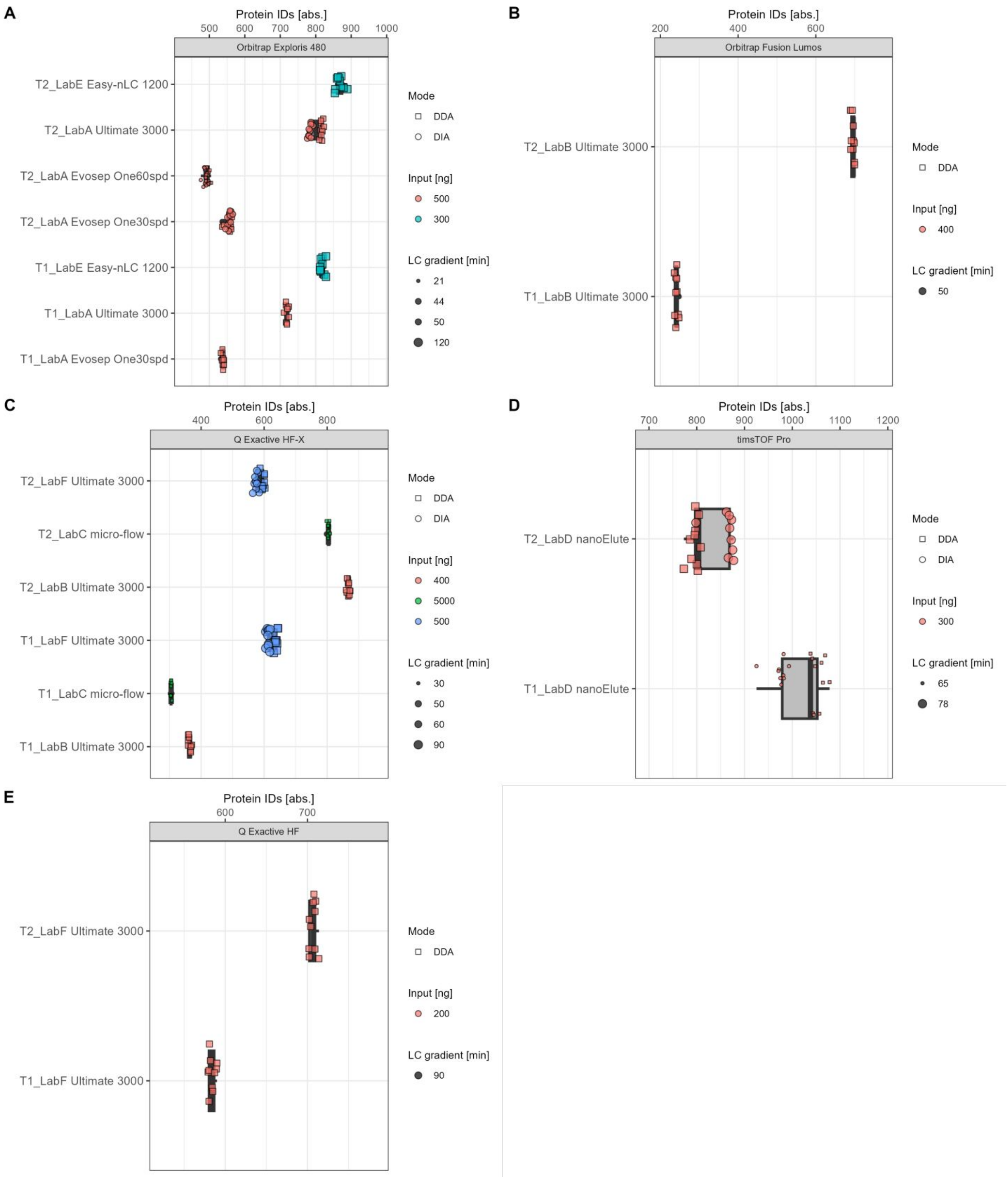


**Fig. S7:** Absolute number of CSF protein IDs for each workflow separated by different MS instruments with color coded input amount.

## 3.2 Protein group-level

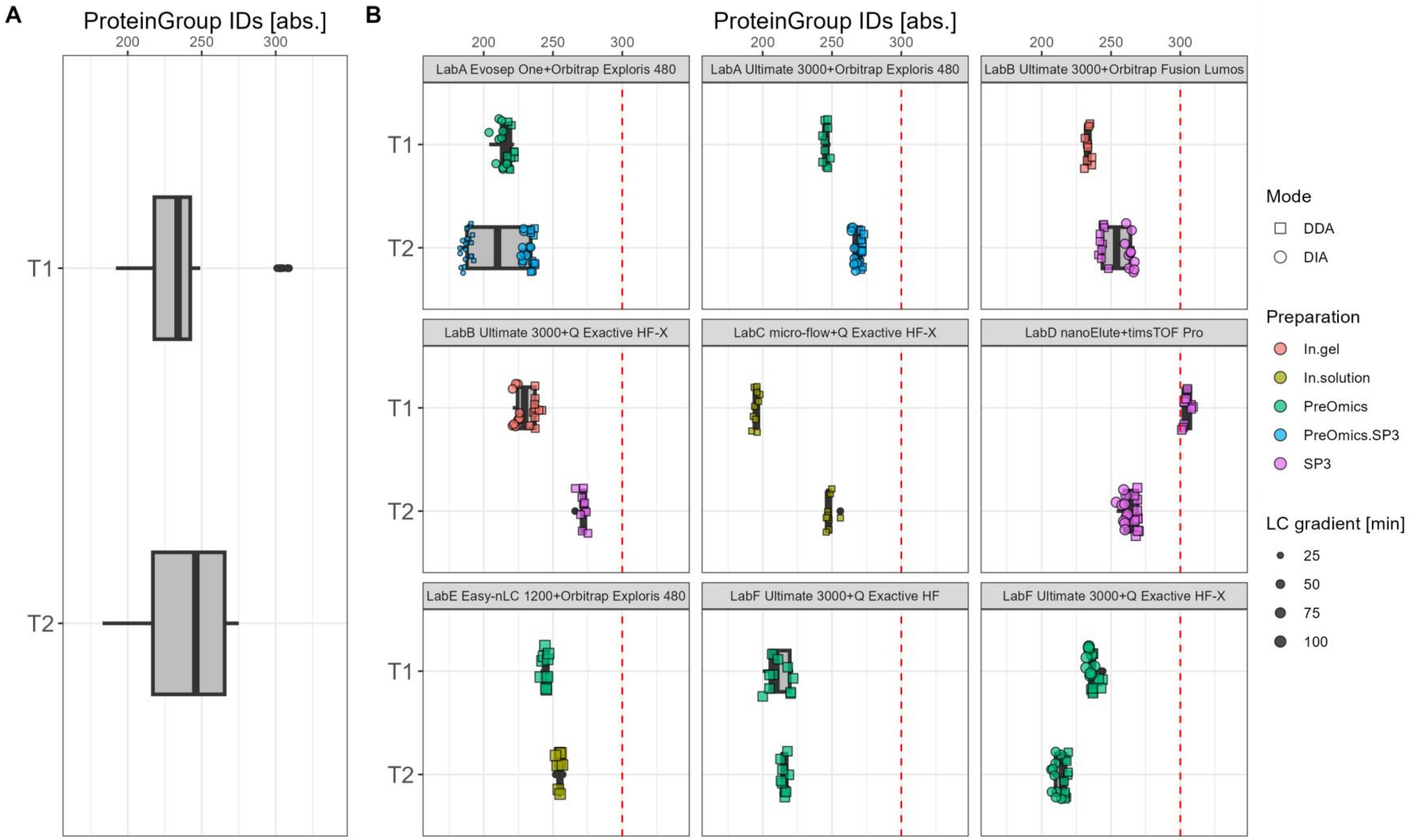


**Fig. S8:** T1 vs. T2 – Absolute number of plasma protein group IDs summarized over all data sets (A) and per specific laboratory LC-MS setup (B). Red dashed line indicates 300 protein group IDs.

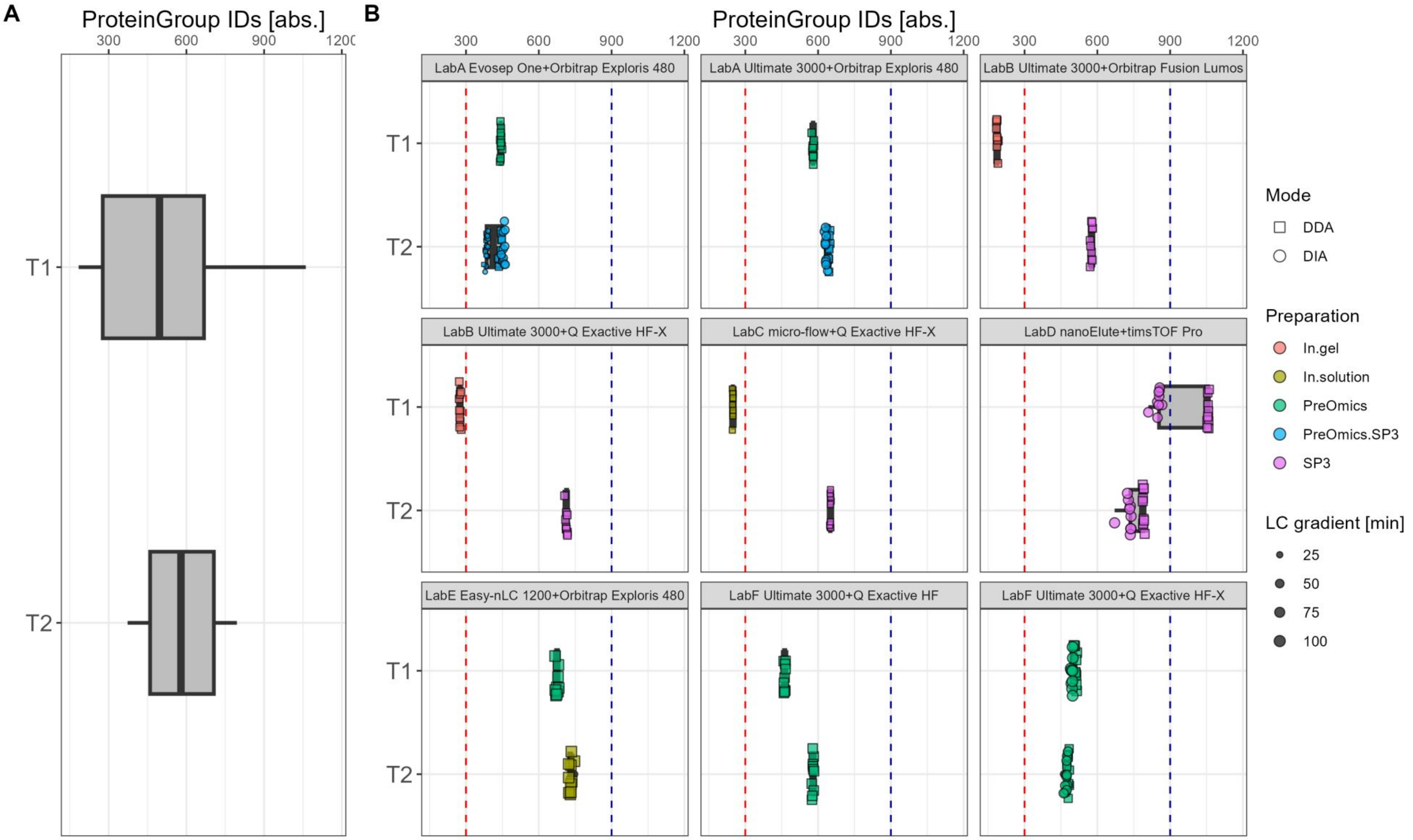


**Fig. S9:** T1 vs. T2 – Absolute number of CSF protein group IDs summarized over all data sets (A) and per specific laboratory LC-MS setup (B). Red dashed line indicates 300 protein group IDs and blue dashed line 900 protein group IDs.

## 3.3 Peptide-level

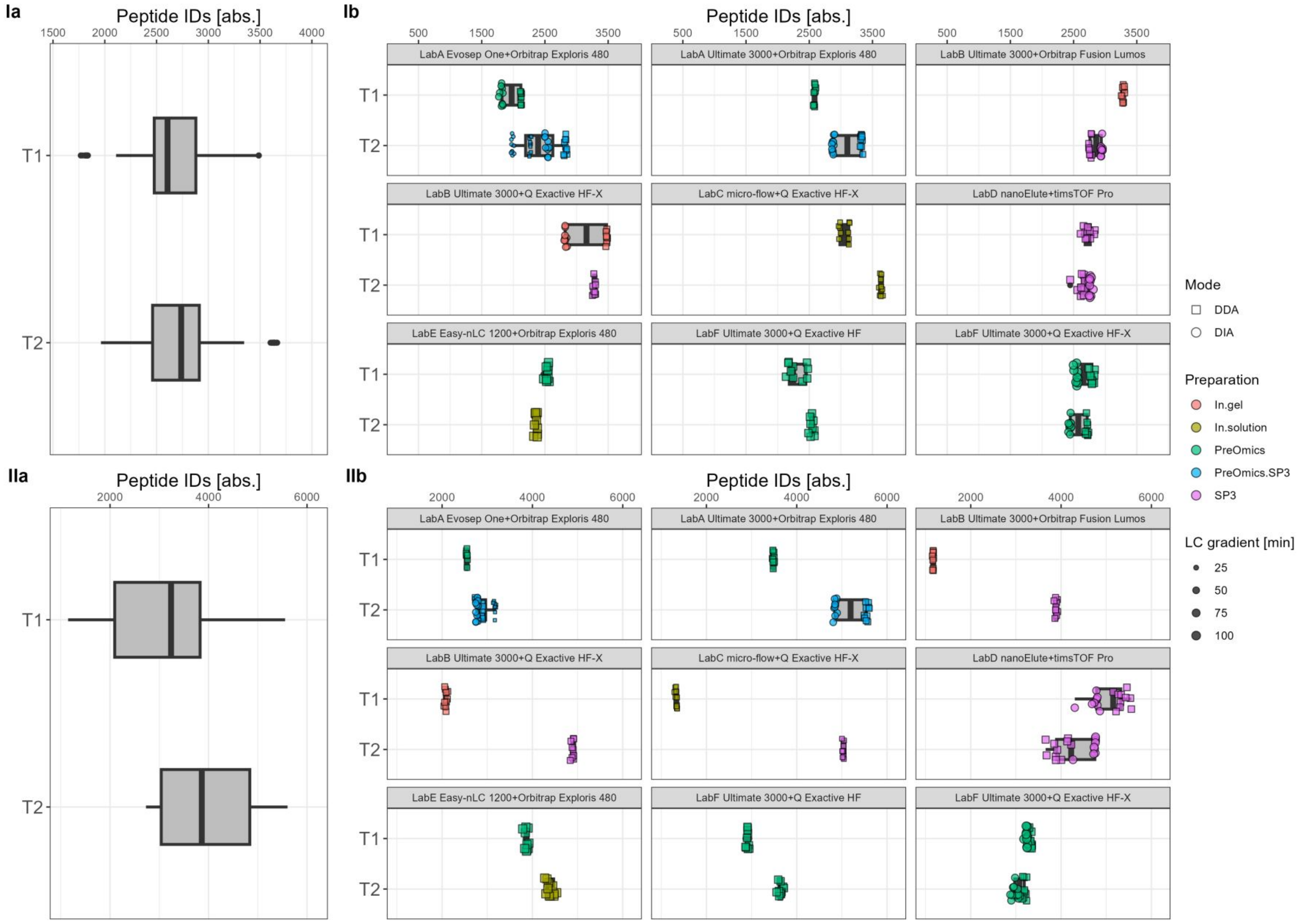


**Fig. S10:** T1 vs. T2 – Absolute numbers of peptide IDs summarized over all data sets (a) and per specific laboratory LC-MS setup (b) for plasma (I) and CSF (II).

## 3.4 Precursor-level

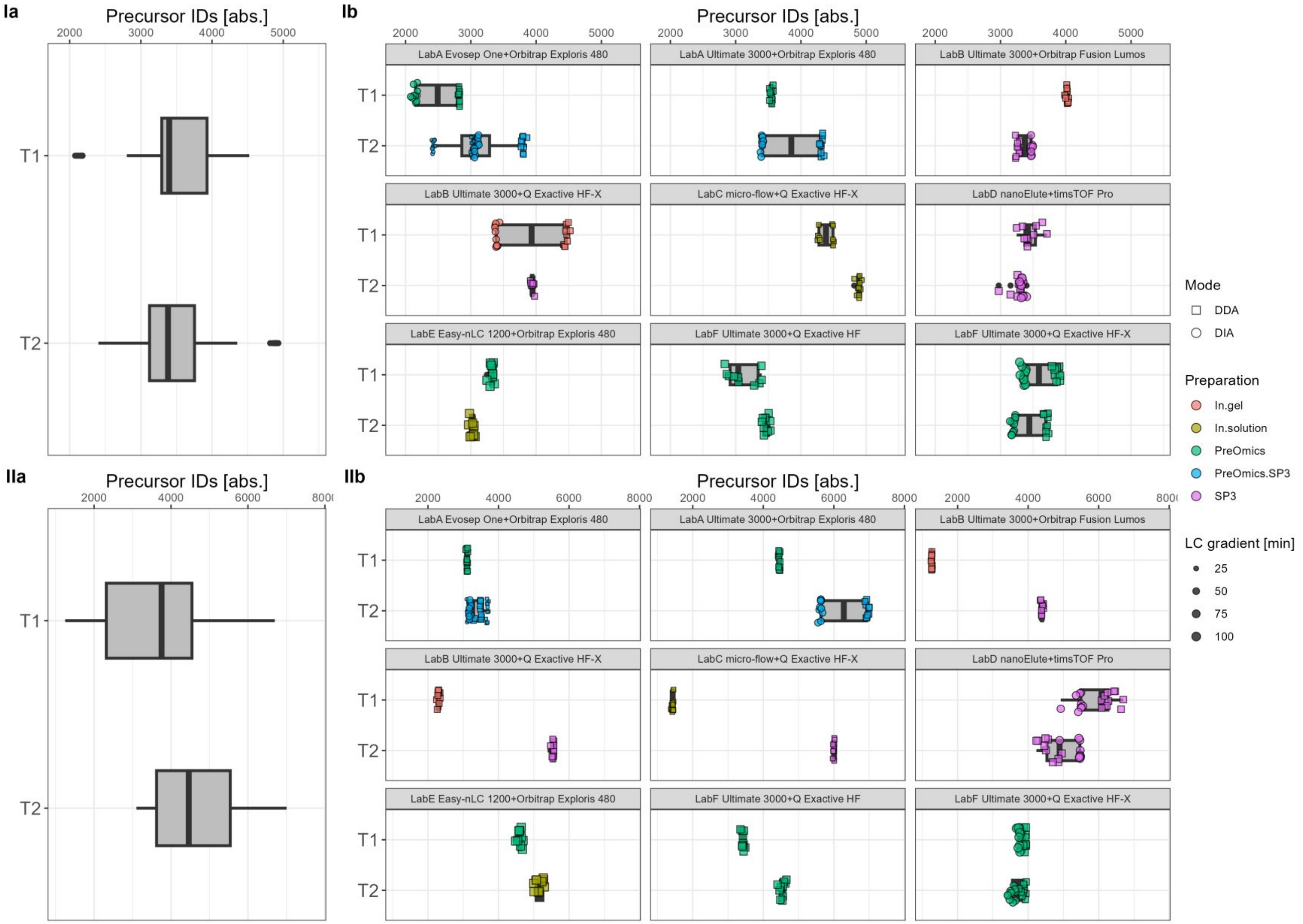


**Fig. S11:** T1 vs. T2 – Absolute number of precursor IDs summarized over all datasets (a) and per specific laboratory LC-MS setup (b) for plasma (I) and CSF (II).

# 4. **Data Completeness - relative**

## 4.1 Precursor-level

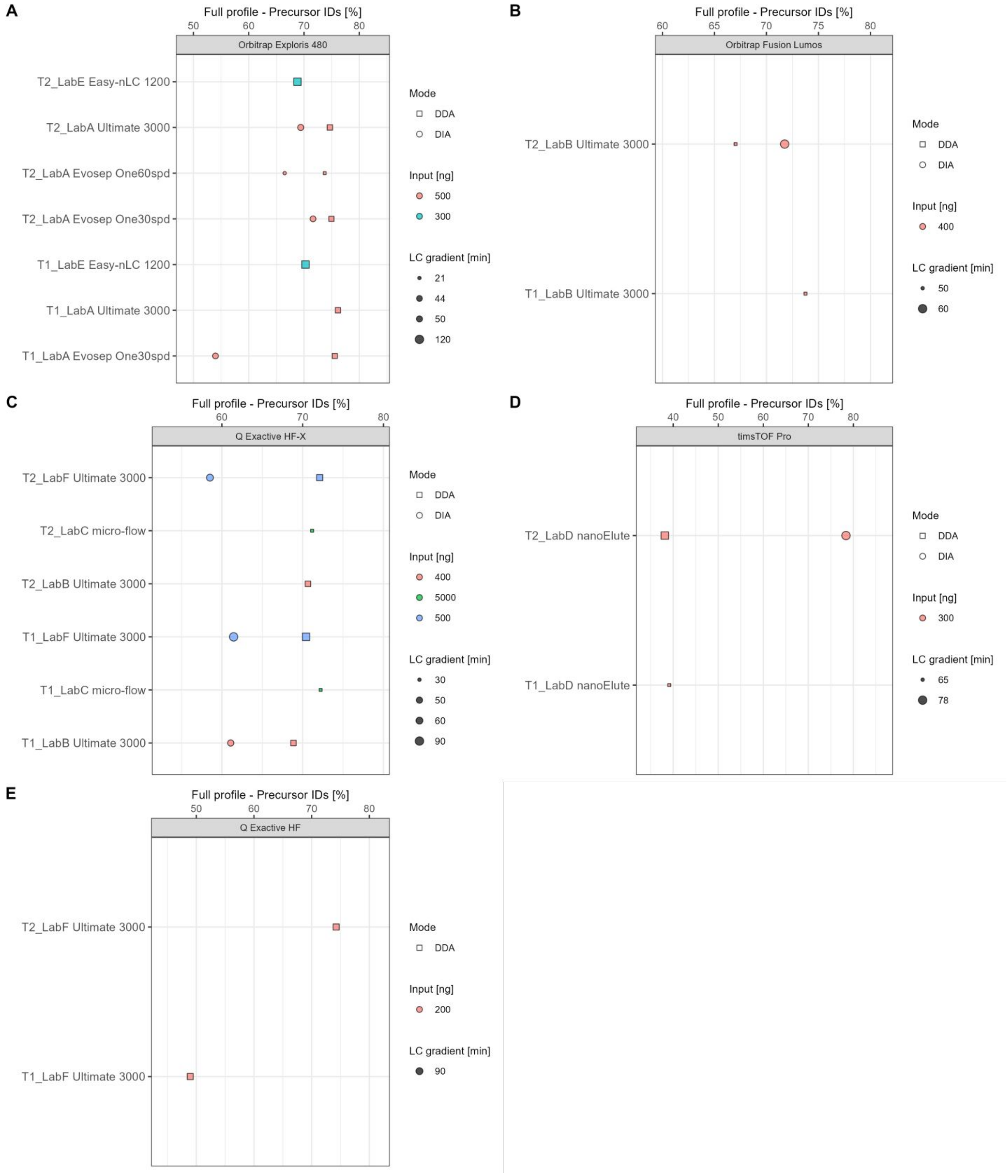


**Fig. S12:** Plasma - Relative data completeness for complete profiles of precursor IDs [%] for each workflow separated by different MS instruments with color coded input amount (A – E).

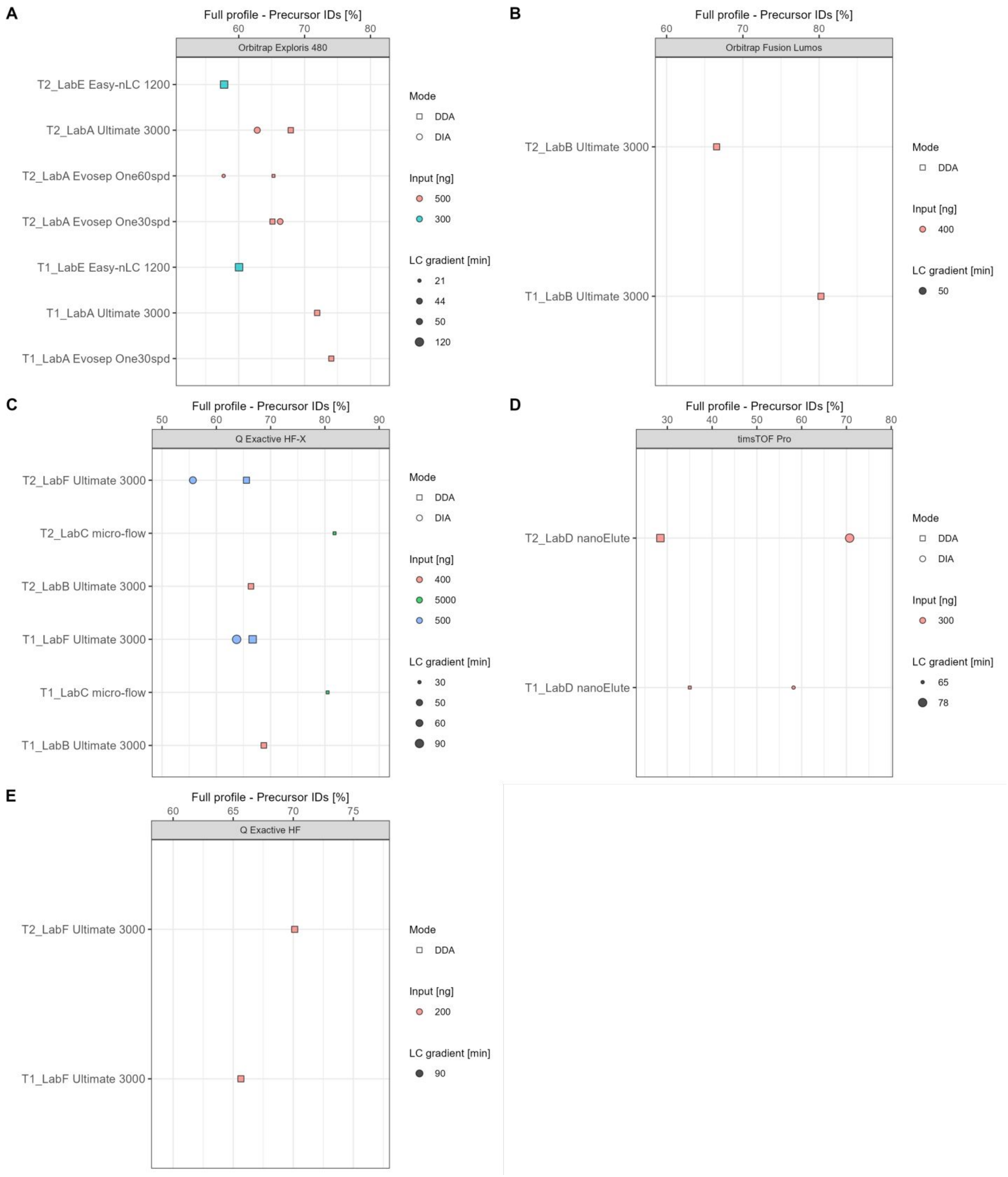


**Fig. S13:** CSF - Relative data completeness for complete profiles of precursor IDs [%] for each workflow separated by different MS instruments with color coded input amount (A – E).

## 4.2 Protein group-level

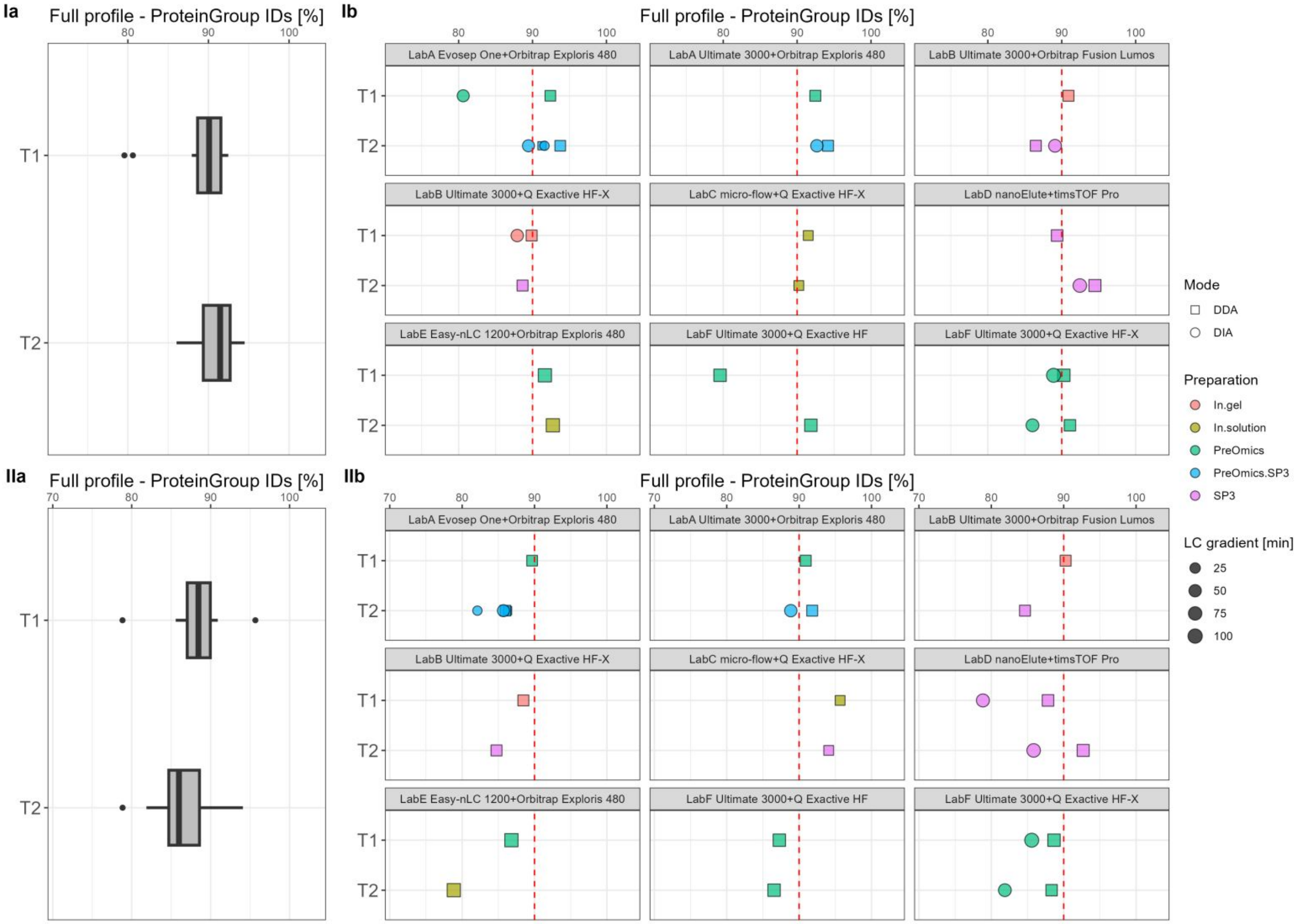


**Fig. S14:** T1 vs. T2 – Relative data completeness for complete profiles of protein group IDs [%] summarized over all analyses (a) and per specific laboratory LC-MS setup (b) for plasma (I) and CSF (II).

## 4.3 Protein-level

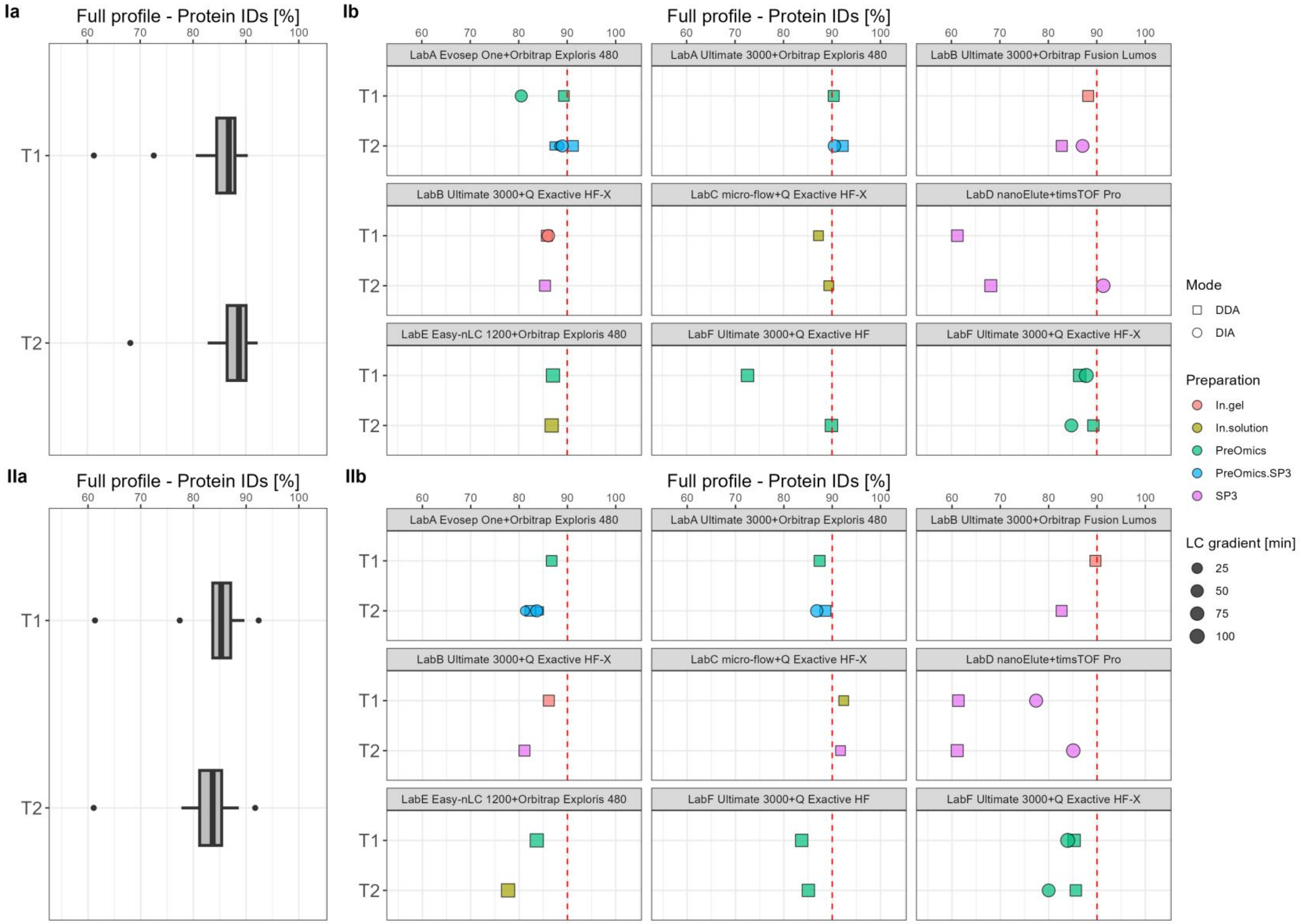


**Fig. S15:** T1 vs. T2 – Relative data completeness for complete profiles of protein IDs [%] summarized over all analyses (a) and per specific laboratory LC-MS setup (b) for plasma (I) and CSF (II).

## 4.4 Peptide-level

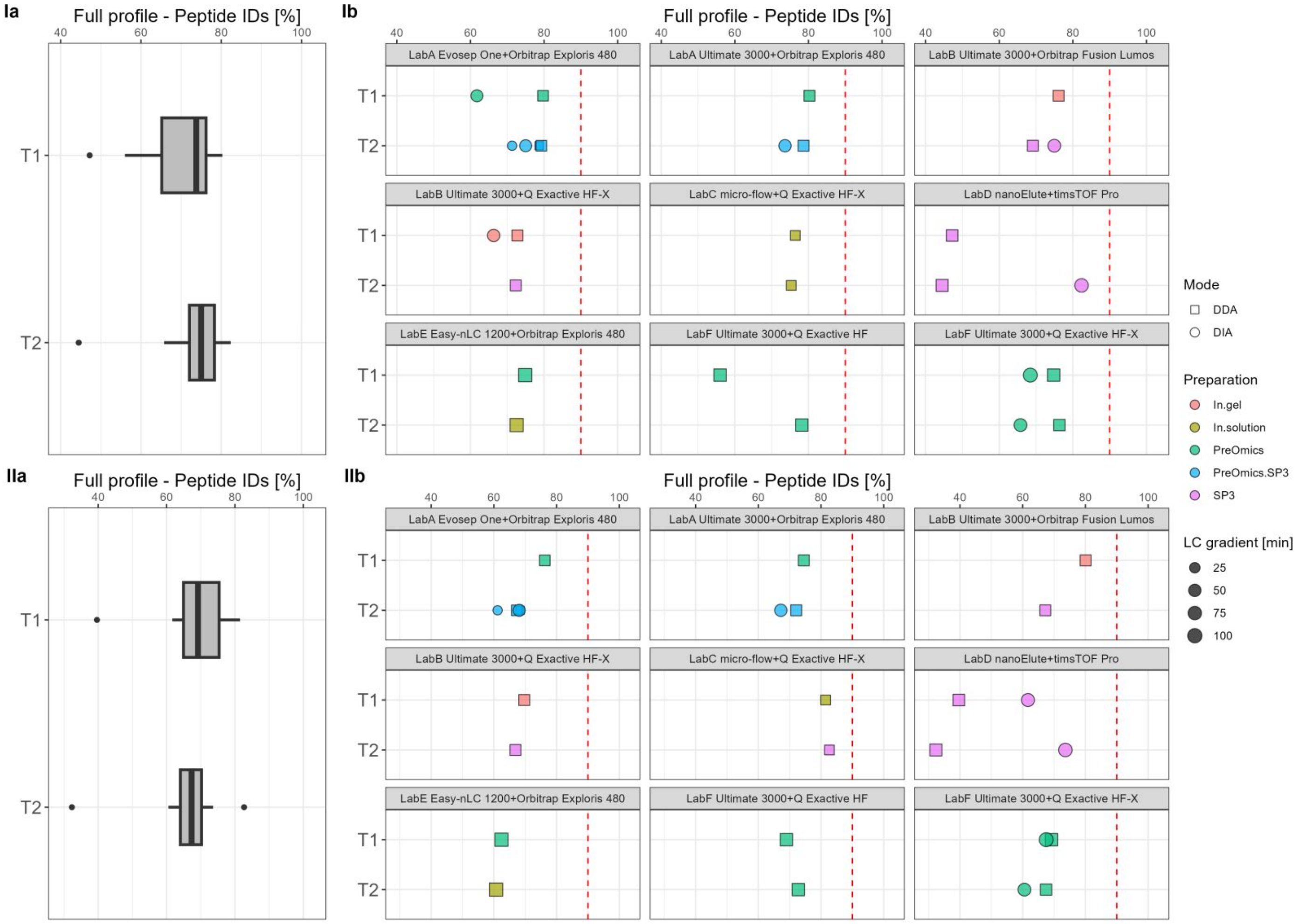


**Fig. S16:** T1 vs. T2 – Relative data completeness for complete profiles of peptide IDs [%] summarized over all analyses (a) and per specific laboratory LC-MS setup (b) for plasma (I) and CSF (II).

# 5. Intensity Distributions – Missed Cleavages

## 5.1 Plasma

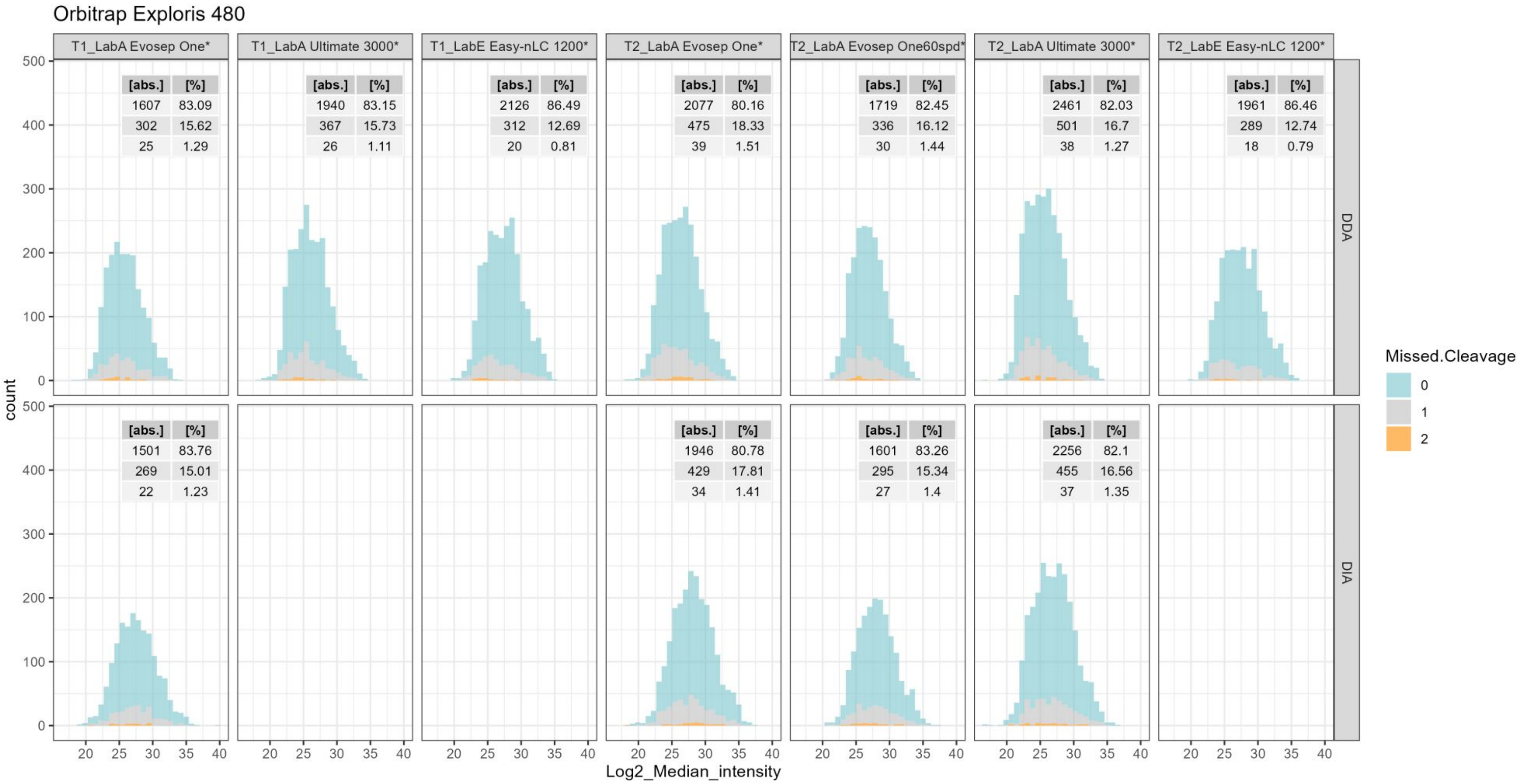


**Fig. S17: Log$_2$ median intensity distribution via stacked histograms on peptide-level for setups with an Orbitrap Exploris 480 MS instrument for plasma.** Missed cleavages are color coded. Tables describe absolute and percentage count per missed cleavage category. The order within tables according to legend. The asterisk in setup name displays workflows with usage of trypsin/Lys-C digestion, otherwise only trypsin.

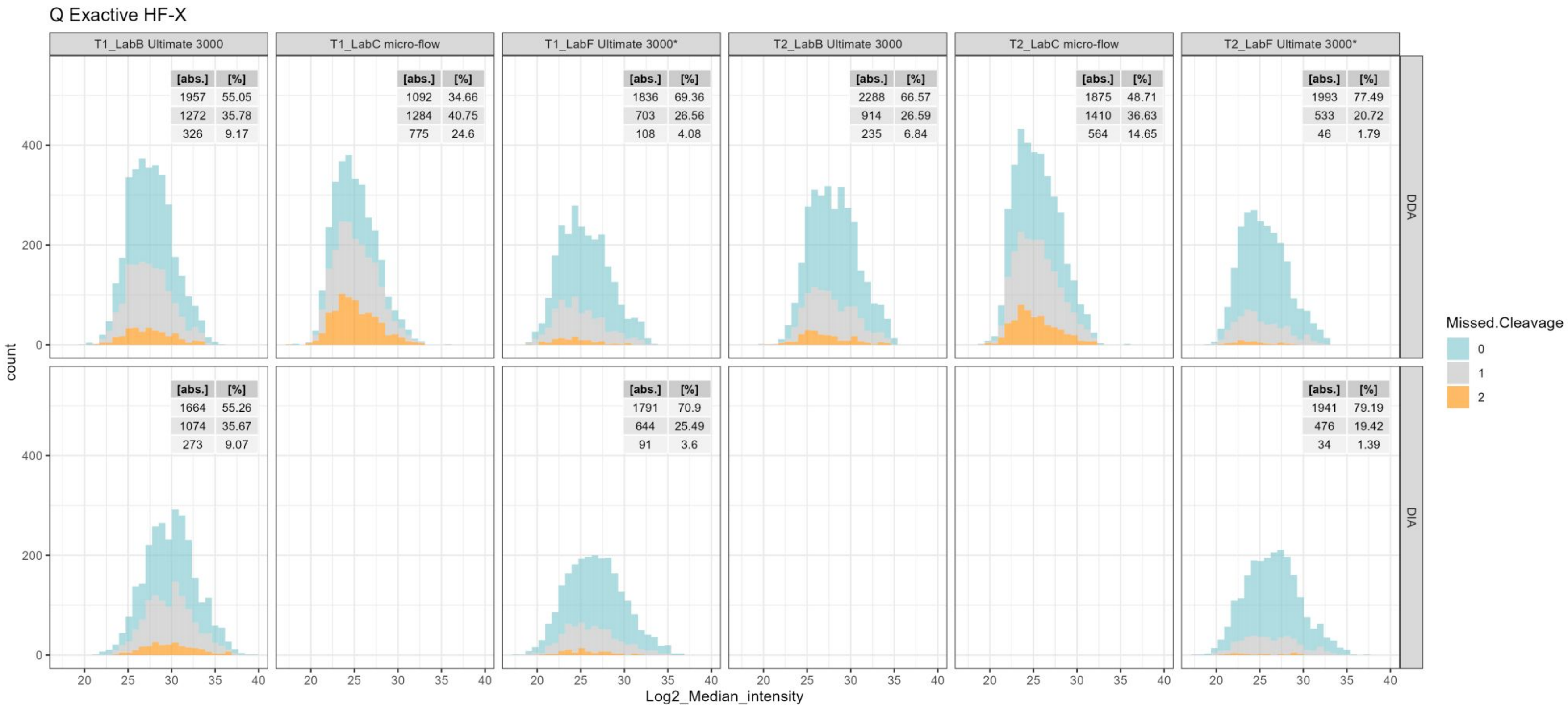


**Fig. S18: Log$_2$ median intensity distribution via stacked histograms on peptide-level for setups with a Q Exactive HF-X MS instrument for plasma.** Missed cleavages are color coded. Tables describe absolute and percentage count per missed cleavage category. The order within tables according to legend. The asterisk in setup name displays workflows with usage of trypsin/Lys-C digestion, otherwise only trypsin.

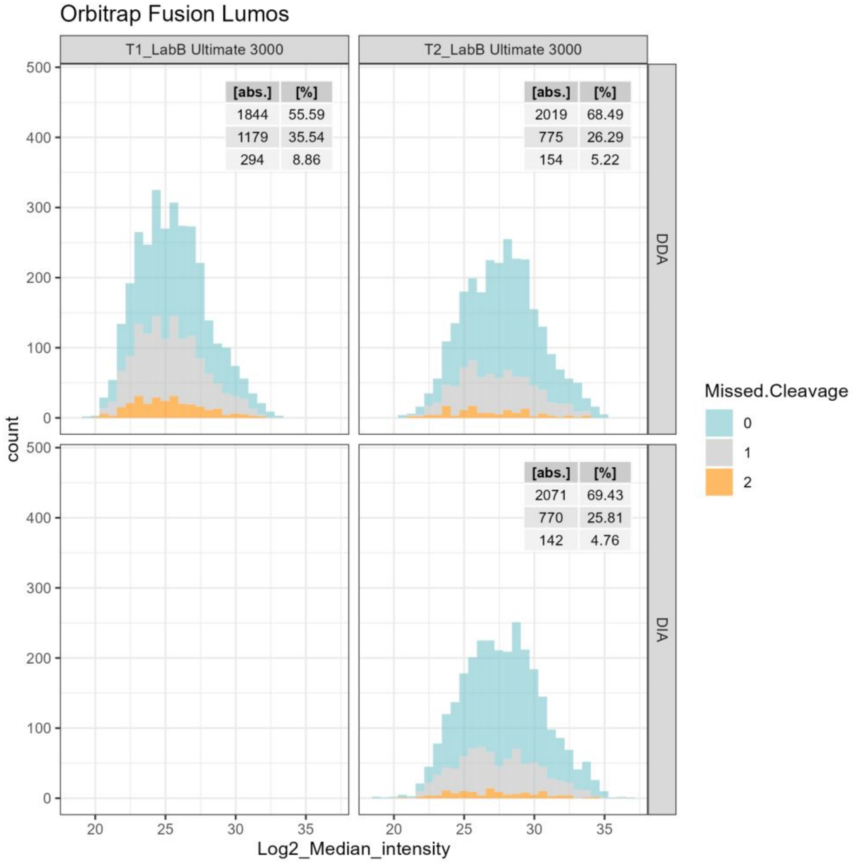


**Fig. S19: $Log_2$ median intensity distribution via stacked histograms on peptide-level for setups with an Orbitrap Fusion Lumos MS instrument for plasma.** Missed cleavages are color coded. Tables describe absolute and percentage count per missed cleavage category. The order within tables according to legend. The asterisk in setup name displays workflows with usage of trypsin/Lys-C digestion, otherwise only trypsin.

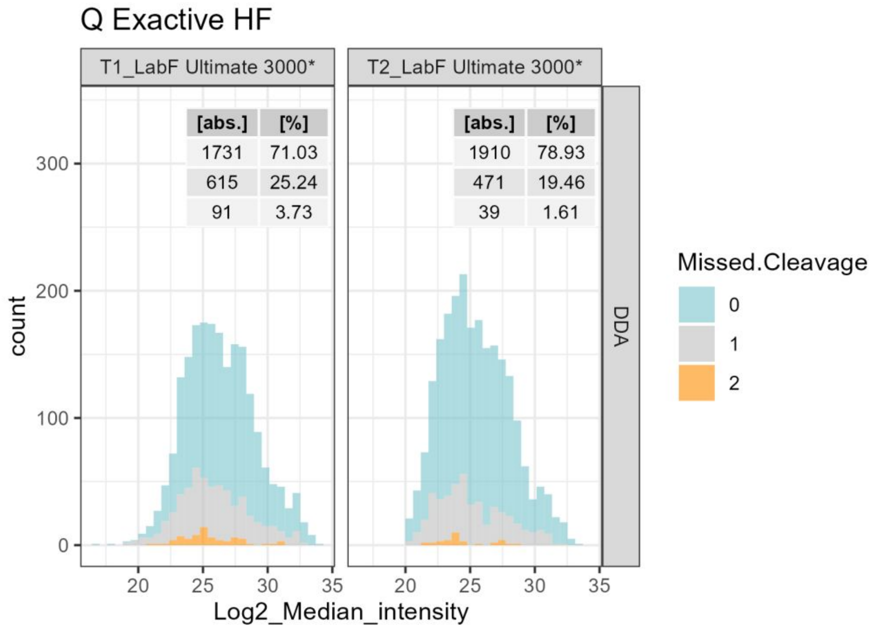


**Fig. S20: Log$_2$ median intensity distribution via stacked histograms on peptide-level for setups with a Q Exactive HF MS instrument for plasma.** Missed cleavages are color coded. Tables describe absolute and percentage count per missed cleavage category. Order is according to legend. The asterisk in setup name displays workflows with usage of Trypsin/Lys-C digestion, otherwise only Trypsin.

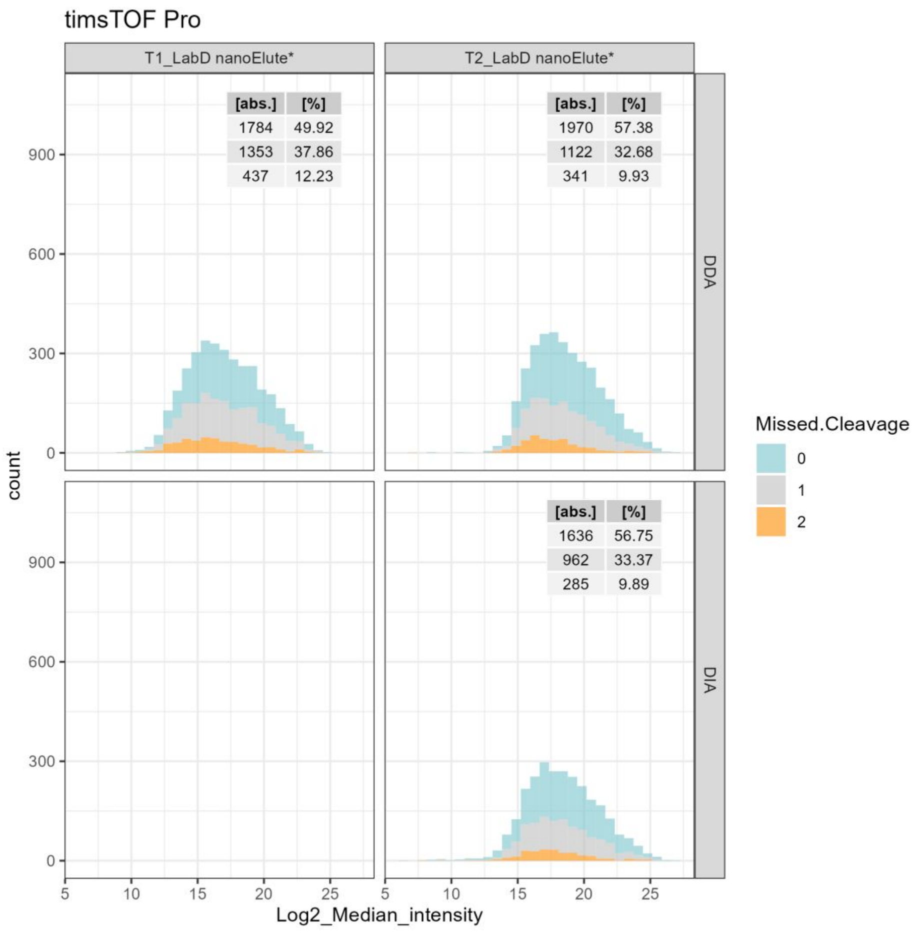


**Fig. S21: Log$_2$ median intensity distribution via stacked histograms on peptide-level for setups with a timsTOF pro MS instrument for plasma.** Missed cleavages are color coded. Tables describe absolute and percentage count per missed cleavage category. Order is according to legend. The asterisk in setup name displays workflows with usage of Trypsin/Lys-C digestion, otherwise only Trypsin.

## 5.2 CSF

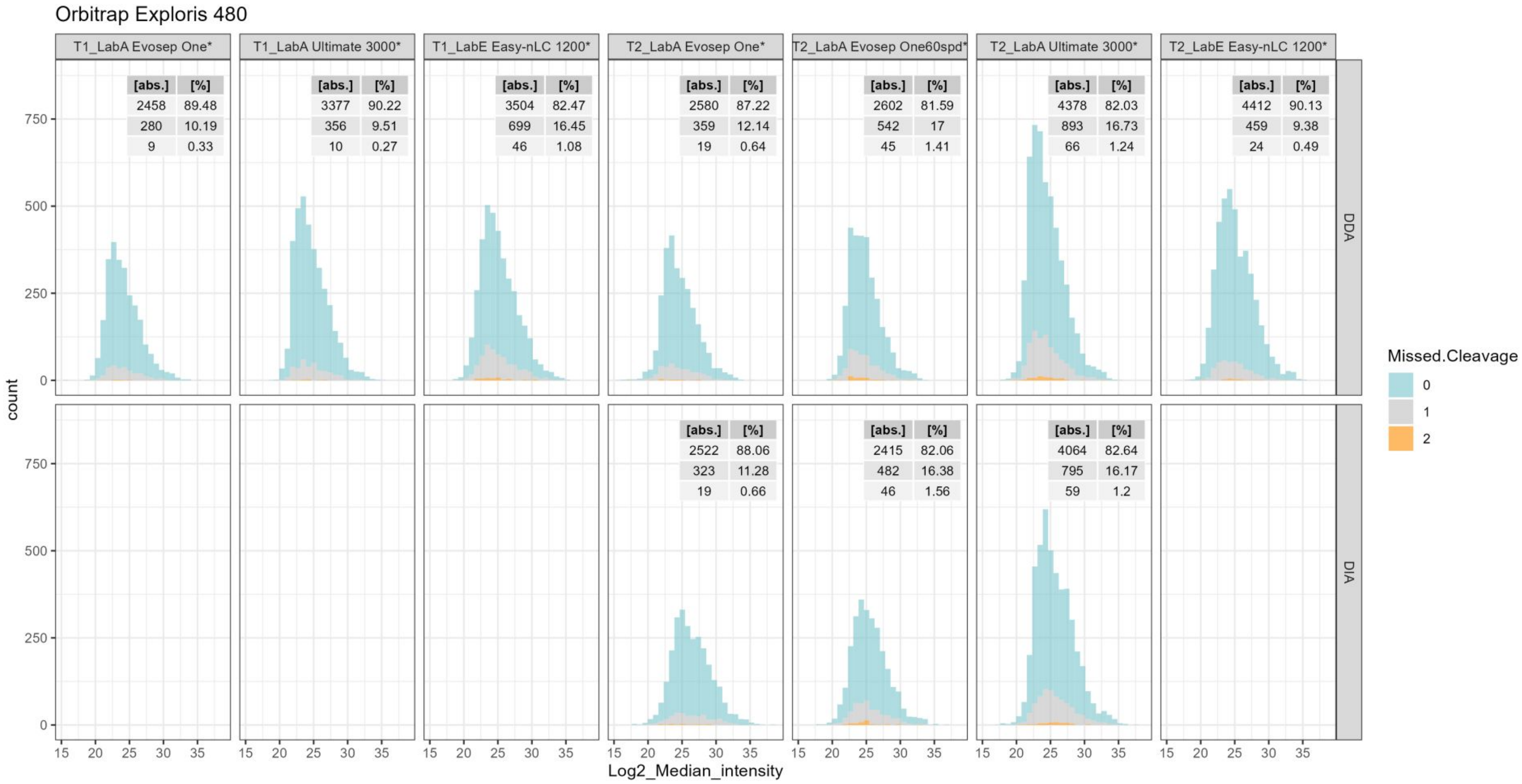


**Fig. S22: Log$_2$ median intensity distribution via stacked histograms on peptide-level for setups with an Orbitrap Exploris 480 MS instrument for CSF.** Missed cleavages are color coded. Tables describe absolute and percentage count per missed cleavage category. Order is according to legend. The asterisk in setup name displays workflows with usage of Trypsin/Lys-C digestion, otherwise only Trypsin.

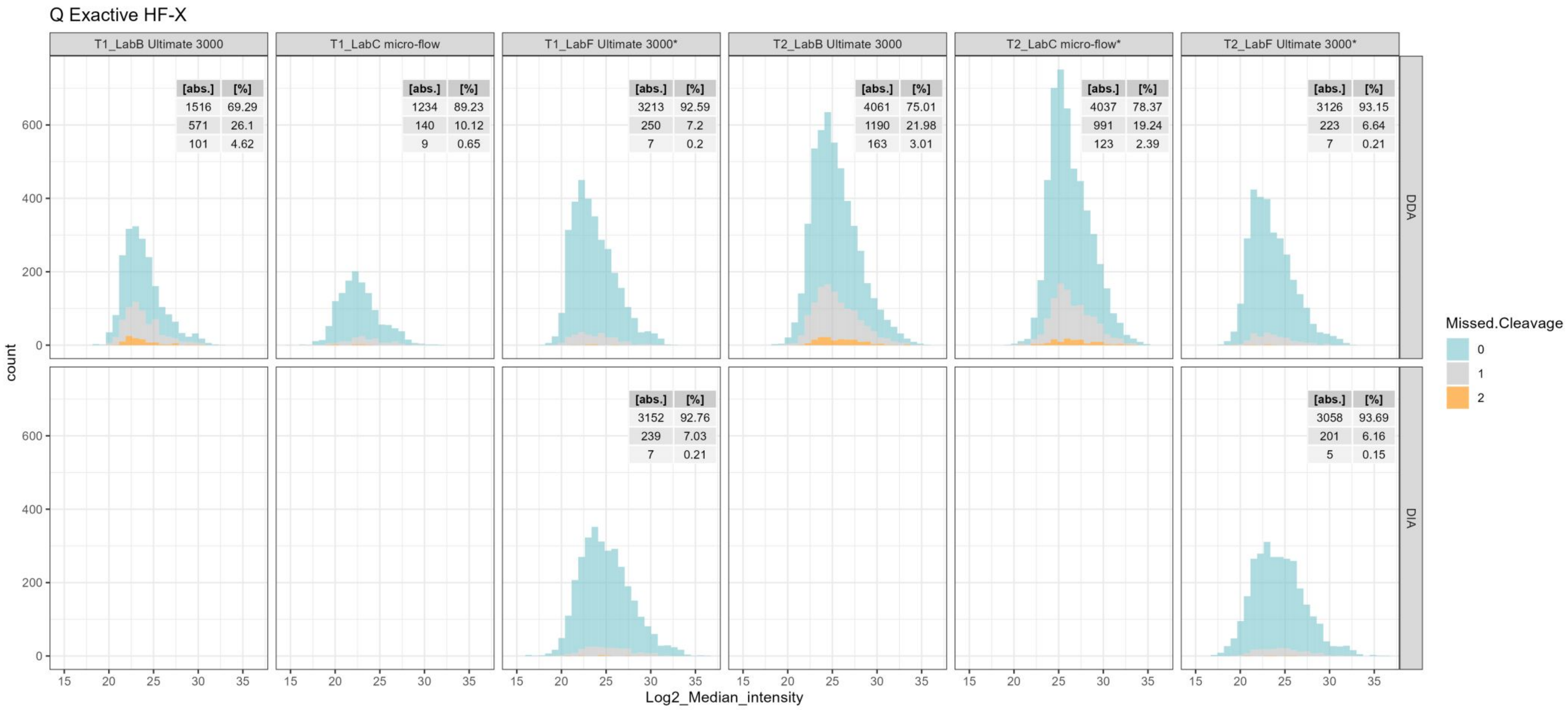


**Fig. S23: Log$_2$ median intensity distribution via stacked histograms on peptide-level for setups with a Q Exactive HF-X MS instrument for CSF.** Missed cleavages are color coded. Tables describe absolute and percentage count per missed cleavage category. Order is according to legend. The asterisk in setup name displays workflows with usage of Trypsin/Lys-C digestion, otherwise only Trypsin.

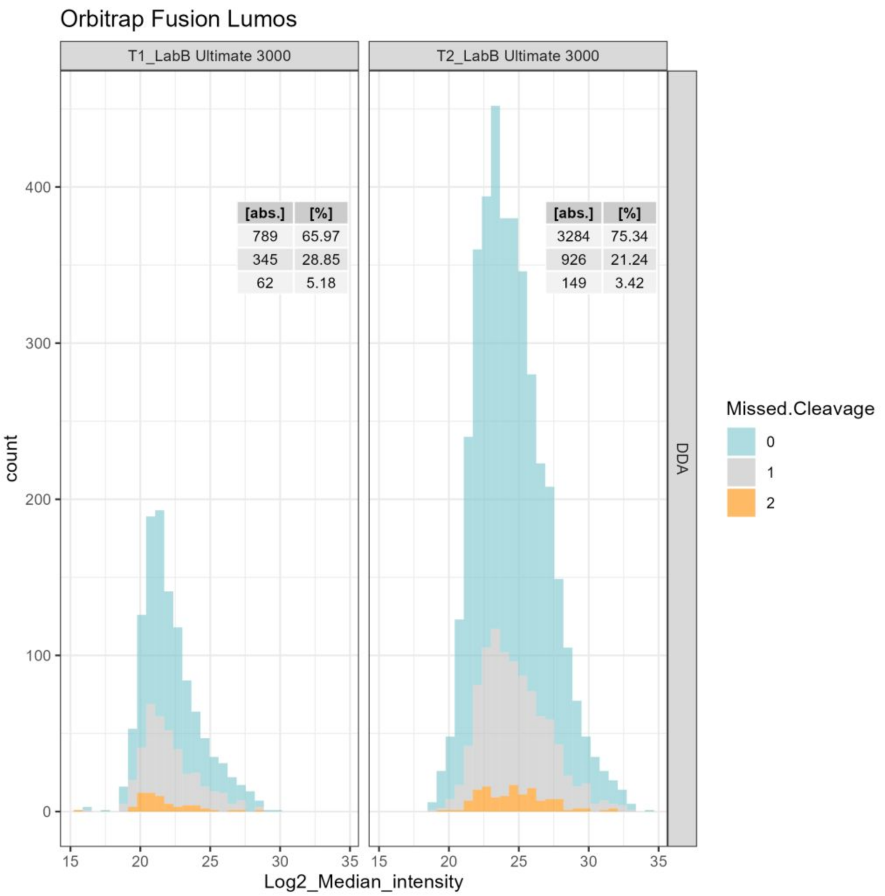


**Fig. S24: Log$_2$ median intensity distribution via stacked histograms on peptide-level for setups with an Orbitrap Fusion Lumos MS instrument for CSF.** Missed cleavages are color coded. Tables describe absolute and percentage count per missed cleavage category. Order is according to legend. The asterisk in setup name displays workflows with usage of Trypsin/Lys-C digestion, otherwise only Trypsin.

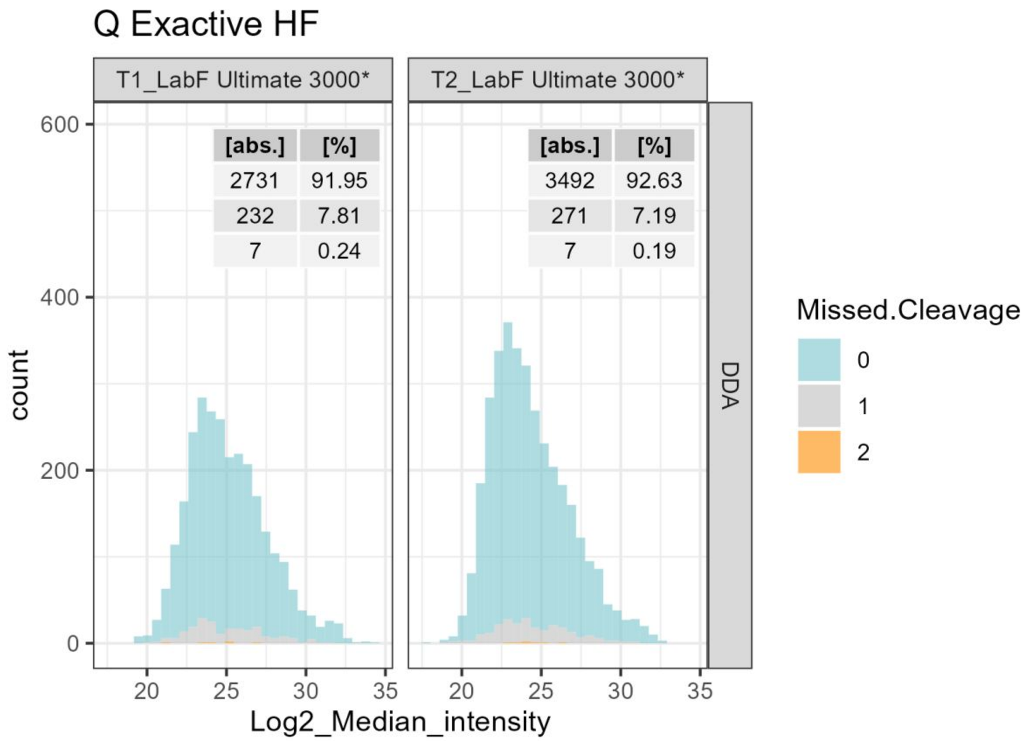


**Fig. S25: $Log_2$ median intensity distribution via stacked histograms on peptide-level for setups with a Q Exactive HF MS instrument for CSF.** Missed cleavages are color coded. Tables describe absolute and percentage count per missed cleavage category. Order is according to legend. The asterisk in setup name displays workflows with usage of Trypsin/Lys-C digestion, otherwise only Trypsin.

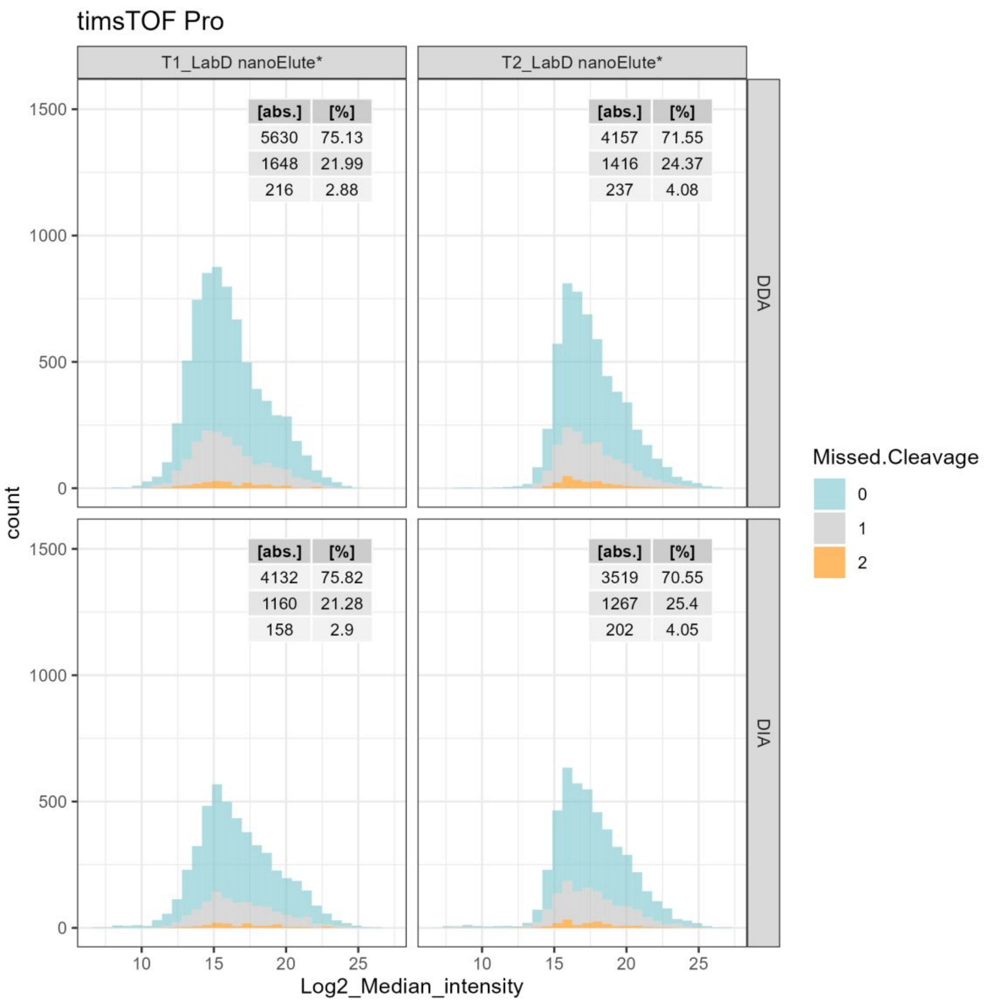


**Fig. S26: Log$_2$ median intensity distribution via stacked histograms on peptide-level for setups with a timsTOF Pro MS instrument for CSF.** Missed cleavages are color coded. Tables describe absolute and percentage count per missed cleavage category. Order is according to legend. The asterisk in setup name displays workflows with usage of Trypsin/Lys-C digestion, otherwise only Trypsin.

# 6. **Missed Cleavages**

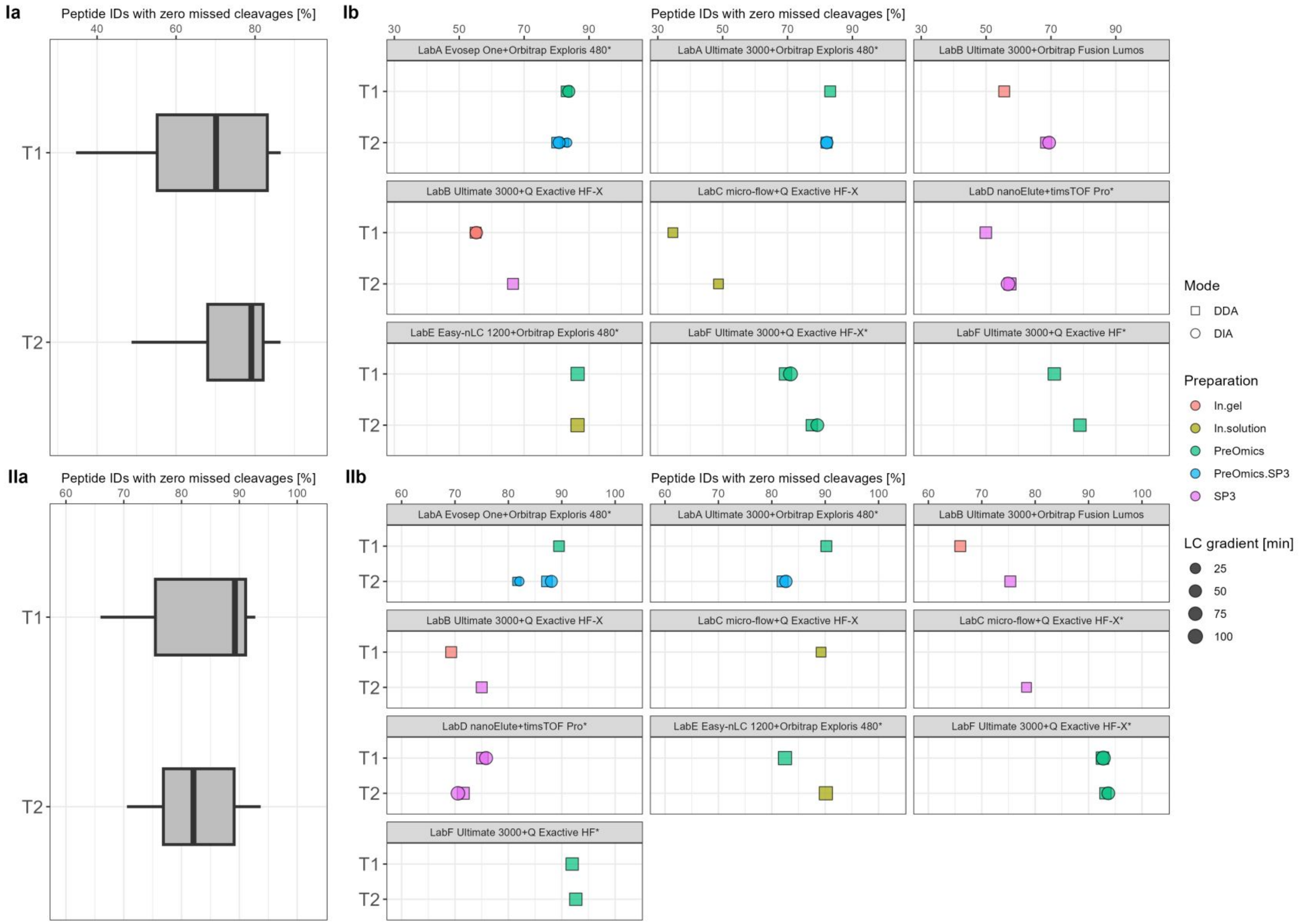


**Fig. S27:** T1 vs. T2 – Peptide IDs [%] with zero missed cleavages summarized over all analyses (a) and per specific laboratory LC-MS setup (b) for plasma (I) and CSF (II).

## 7. **Inter-laboratory reproducibility**

### 7.1 T1 vs T2: Plasma

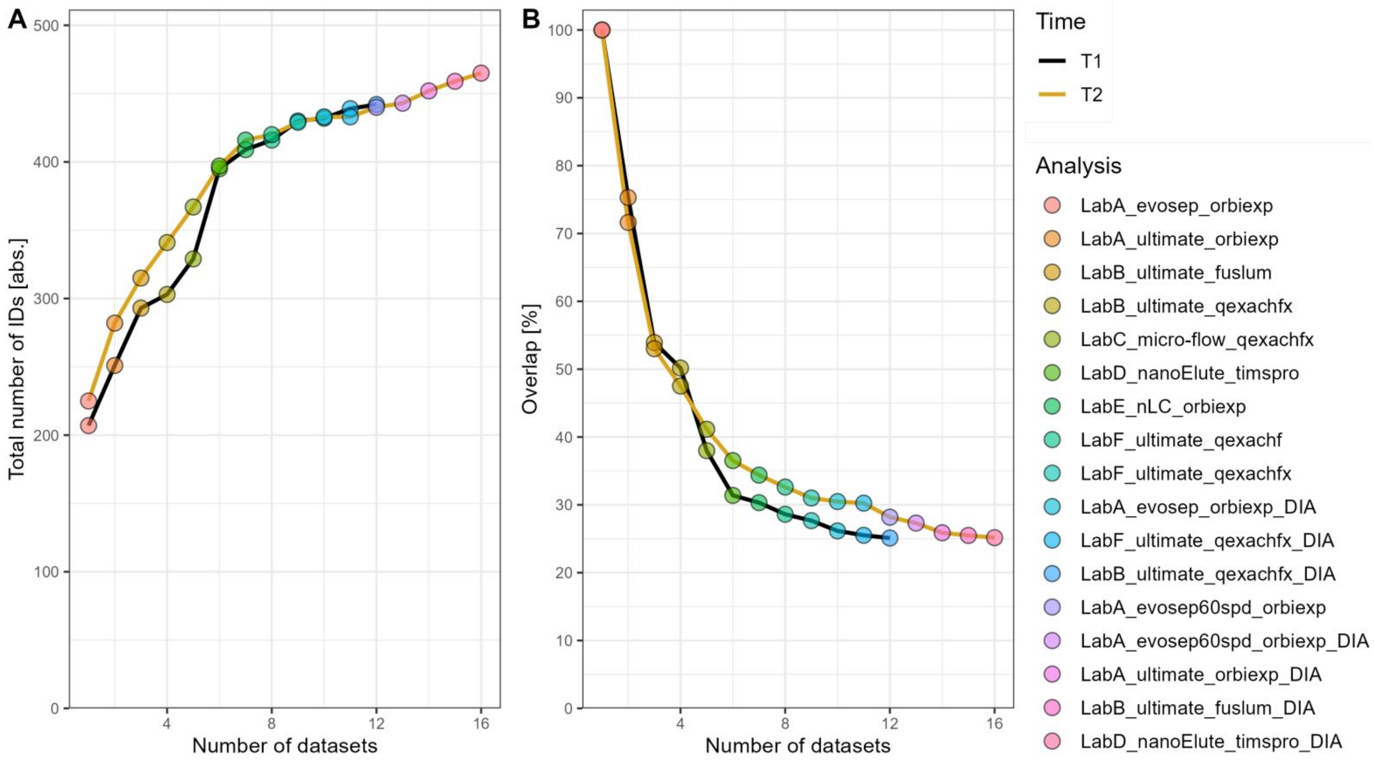


**Fig. S28: Total number of protein IDs [abs.] (A) and overlapping protein IDs [%] (B) for plasma per measurement round.** (A) – Starting with the dataset from LabA_evosep_orbiexp the protein IDs of the next dataset are added and duplicated entries removed to get a total number of protein IDs. This process is performed for T1 and T2 and the respective datasets. (B) – The relative overlap is calculated by dividing the overlapping number of protein IDs by the total number of IDs times 100. This is applied by starting with the dataset from LabA_evosep_orbiexp and continuing with the next datasets for T1 and T2, respectively. Note that both for (A) and (B) only full profiles are considered (100% data completeness).

## 7.2 T1 vs T2: CSF

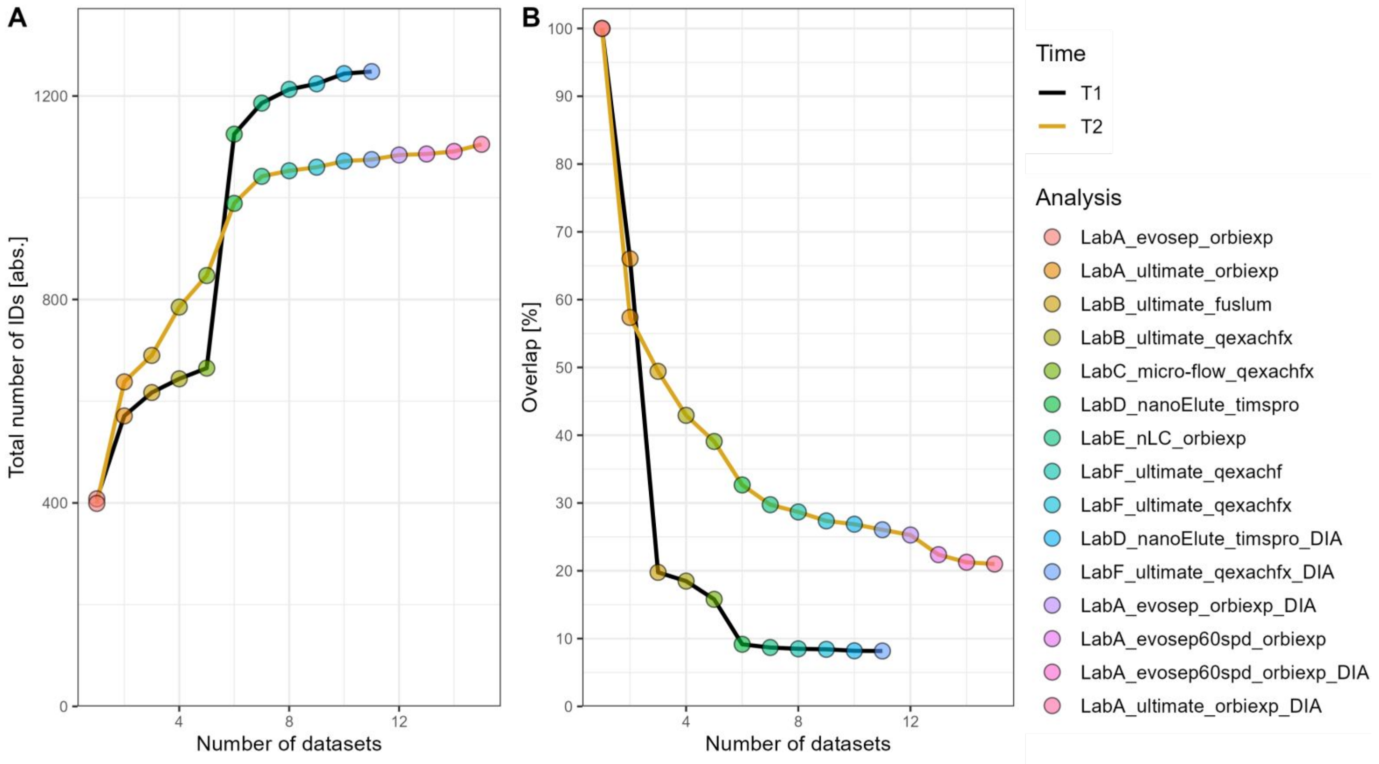


**Fig. S29: Total number of protein IDs [abs.] (A) and overlapping protein IDs [%] (B) for CSF per measurement round.** (A) – Starting with the dataset from LabA_evosep_orbiexp the protein IDs of the next dataset are added and duplicated entries removed to get a total number of protein IDs. This process is performed for T1 and T2 and the respective datasets. (B) – The relative overlap is calculated by dividing the overlapping number of protein IDs by the total number of IDs times 100. This is applied by starting with the dataset from LabA_evosep_orbiexp and continuing with the next datasets for T1 and T2, respectively. Note that both for (A) and (B) only full profiles are considered (100% data completeness).

## 7.3 T2: Plasma – interlaboratory-reproducibility depending on data completeness

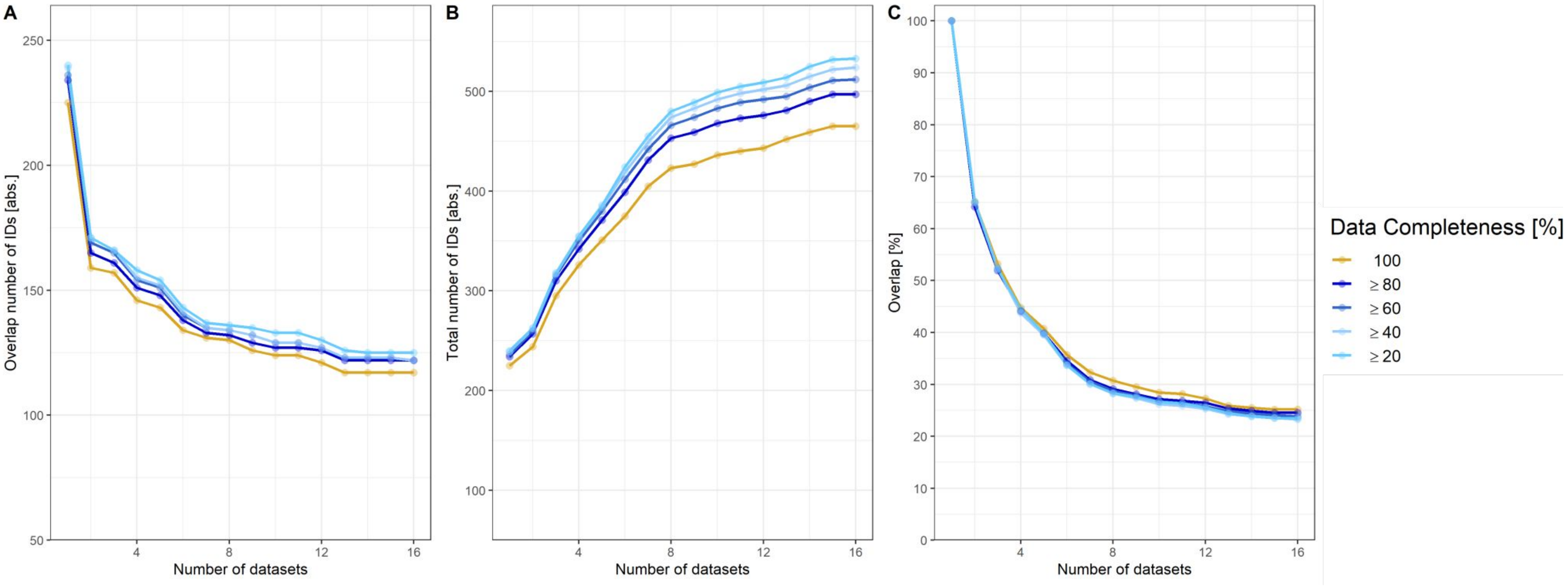


**Fig. S30: T2 – overlapping protein IDs [abs.] (A), total number of protein IDs [abs.] (B) and overlapping protein IDs [%] (C) for plasma with distinct levels of data completeness [%].** (A) – Starting with one dataset the protein IDs of the next dataset are added and only common protein IDs are kept. This process is performed across all datasets from T2. (B) – Starting with one dataset the protein IDs of the next dataset are added and duplicated entries removed to get a total number of protein IDs. This process is performed across all datasets from T2. (C) – The relative overlap is calculated by dividing the overlapping number of protein IDs by the total number of IDs times 100. This is applied by starting with one dataset and continuing with the next dataset for T2. Note that (A-C) is applied for distinct levels of data completeness, e.g., 100% data completeness refers to being present in each of the 10 technical replicates or at least 80% data completeness refers to being present in at least 8 technical replicates etc.

## 7.4 T2: CSF - interlaboratory-reproducibility depending on data completeness

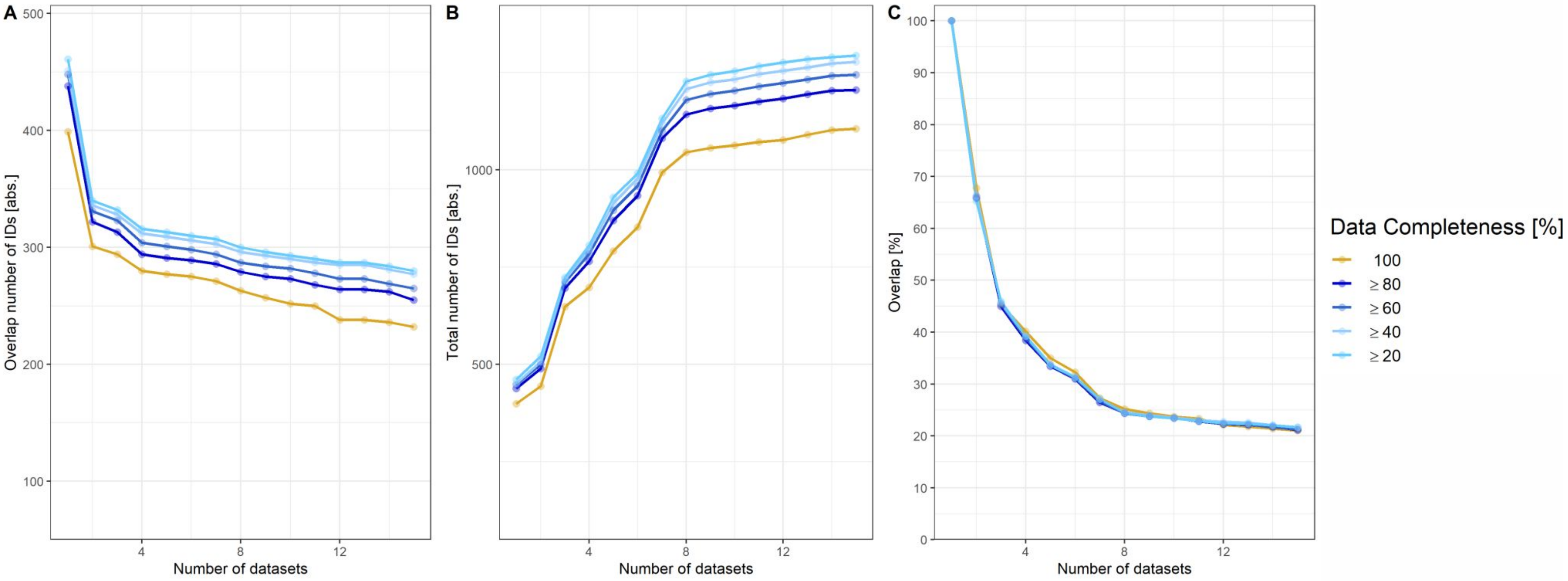


**Fig. S31: T2 – overlapping protein IDs [abs.] (A), total number of protein IDs [abs.] (B) and overlapping protein IDs [%] (C) for CSF with distinct levels of data completeness [%].** (A) – Starting with one dataset the protein IDs of the next dataset are added and only common protein IDs are kept. This process is performed across all datasets from T2. (B) – Starting with one dataset the protein IDs of the next dataset are added and duplicated entries removed to get a total number of protein IDs. This process is performed across all datasets from T2. (C) – The relative overlap is calculated by dividing the overlapping number of protein IDs by the total number of IDs times 100. This is applied by starting with one dataset and continuing with the next dataset for T2. Note that (A-C) is applied for distinct levels of data completeness, e.g., 100% data completeness refers to being present in each of the 10 technical replicates or at least 80% data completeness refers to being present in at least 8 technical replicates etc.

## 7.5 T1 and T2 combined: Plasma – interlaboratory-reproducibility depending on data completeness

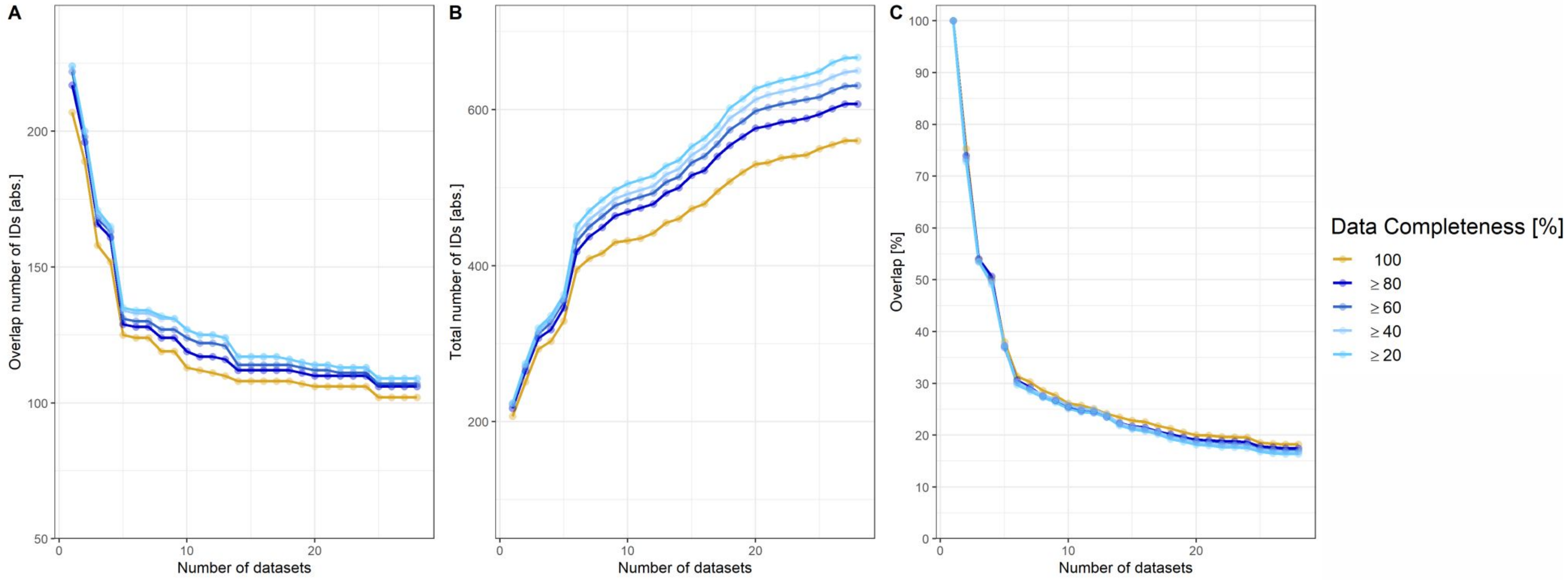


**Fig. S32: T1 and T2 combined – overlapping protein IDs [abs.] (A), total number of protein IDs [abs.] (B) and overlapping protein IDs [%] (C) for plasma with distinct levels of data completeness [%].** (A) – Starting with one dataset the protein IDs of the next dataset are added and only common protein IDs are kept. This process is performed across all datasets from T1 and T2. (B) – Starting with one dataset the protein IDs of the next dataset are added and duplicated entries removed to get a total number of protein IDs. This process is performed across all datasets from T1 and T2. (C) – The relative overlap is calculated by dividing the overlapping number of protein IDs by the total number of IDs times 100. This is applied by starting with one dataset and continuing with the next dataset for both T1 and T2. Note that (A-C) is applied for distinct levels of data completeness, e.g., 100% data completeness refers to being present in each of the 10 technical replicates or at least 80% data completeness refers to being present in at least 8 technical replicates etc.

### 7.6 T1 and T2 combined: CSF – interlaboratory-reproducibility depending on data completeness

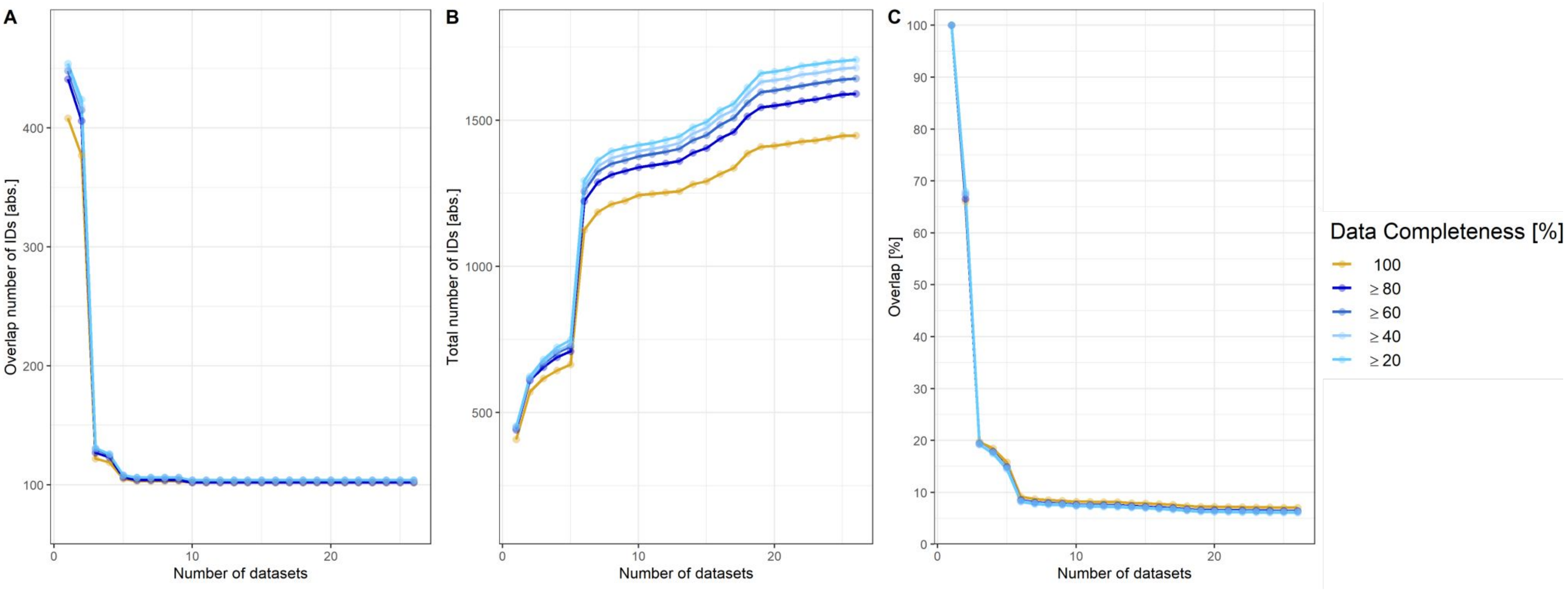


**Fig. S33: T1 and T2 combined – overlapping protein IDs [abs.] (A), total number of protein IDs [abs.] (B) and overlapping protein IDs [%] (C) for CSF with distinct levels of data completeness [%].** (A) – Starting with one dataset the protein IDs of the next dataset are added and only common protein IDs are kept. This process is performed across all datasets from T1 and T2. (B) – Starting with one dataset the protein IDs of the next dataset are added and duplicated entries removed to get a total number of protein IDs. This process is performed across all datasets from T1 and T2. (C) – The relative overlap is calculated by dividing the overlapping number of protein IDs by the total number of IDs times 100. This is applied by starting with one dataset and continuing with the next dataset for both T1 and T2. Note that (A-C) is applied for distinct levels of data completeness, e.g., 100% data completeness refers to being present in each of the 10 technical replicates or at least 80% data completeness refers to being present in at least 8 technical replicates etc.

# 8. **Miscellaneous**

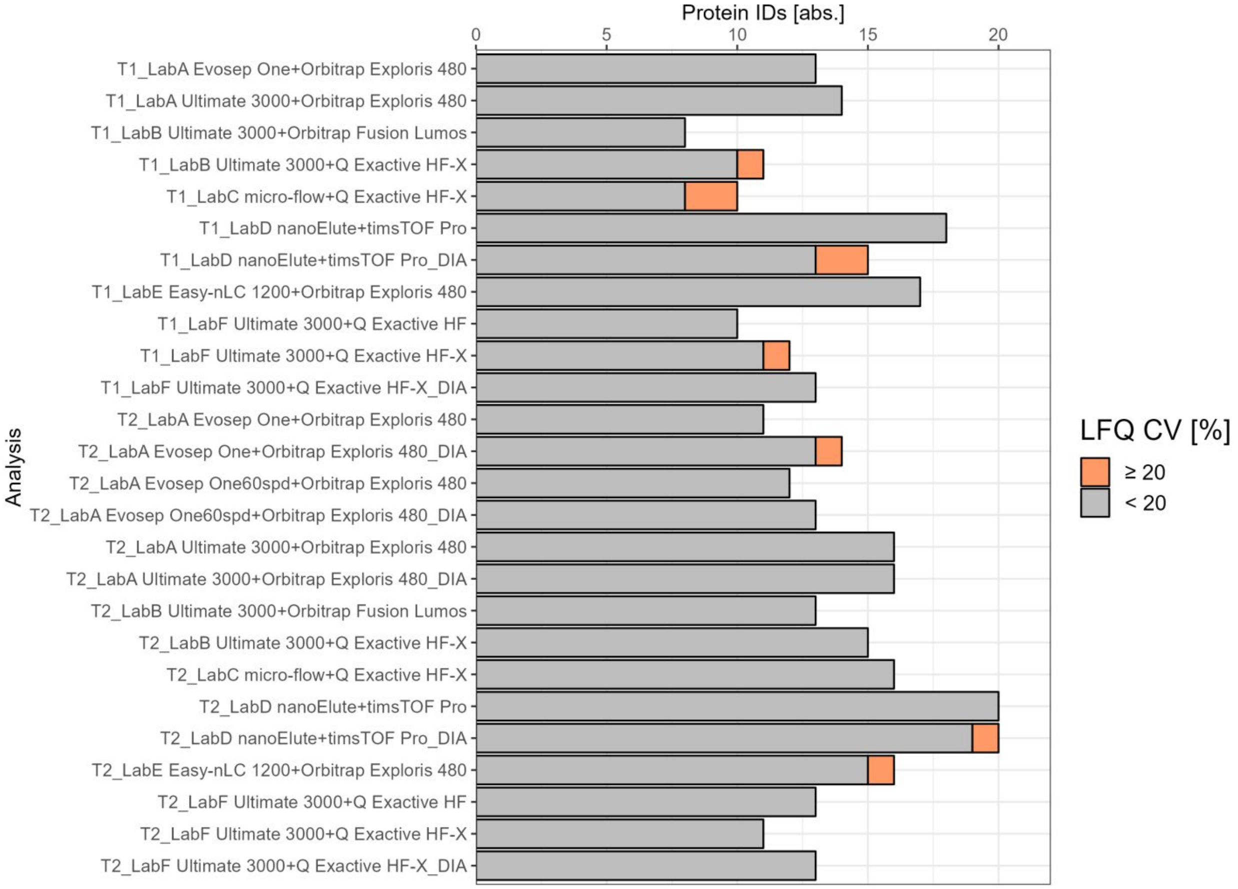


**Fig. S34:** For each dataset, the detected CSF proteins with full profiles are matched against 48 known CSF-related biomarkers. For the matched proteins the quantitative precision of the LFQ intensities is highlighted.

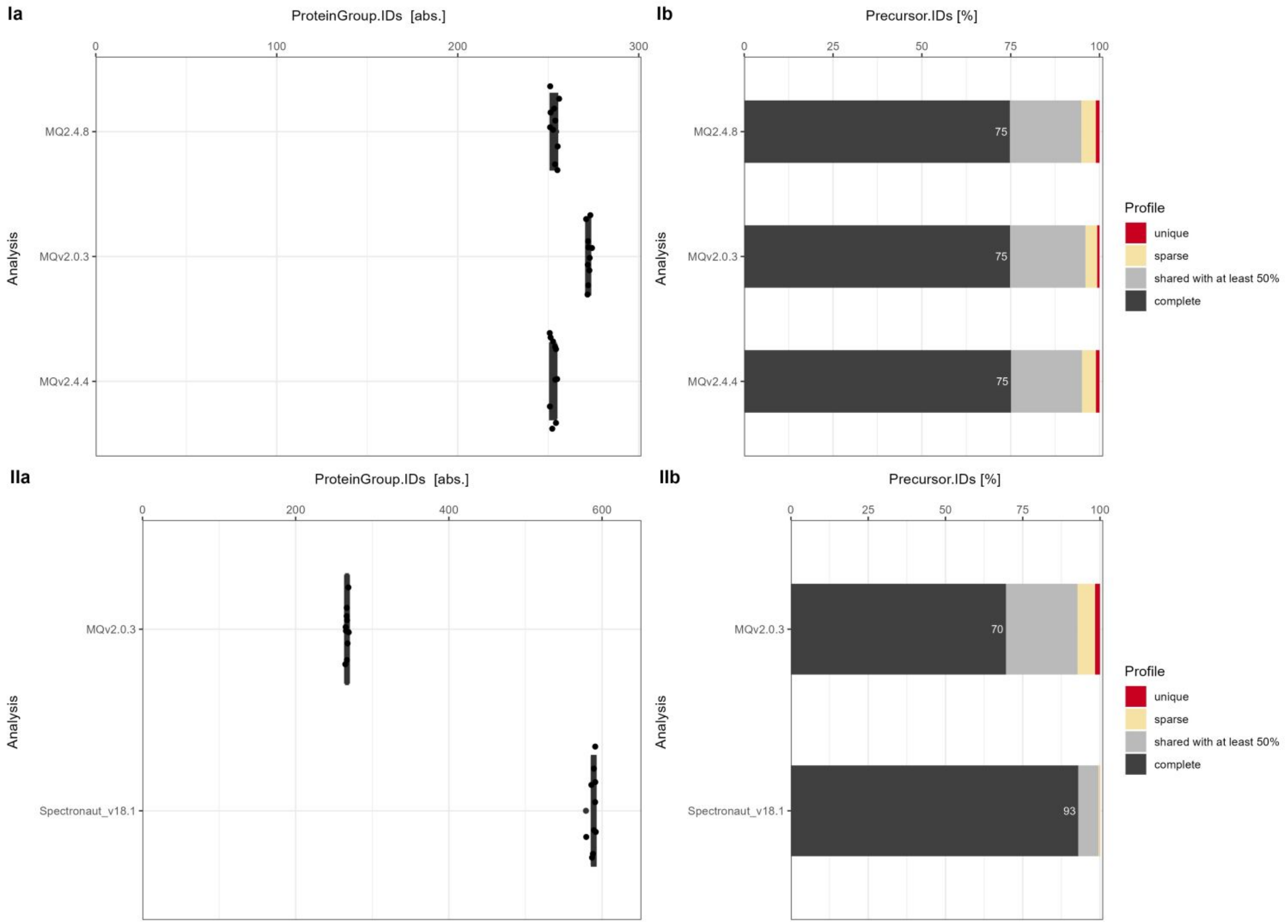


**Fig. S35: Software comparison for T2 LabA Evosep One + Orbitrap Exploris 480.** Number of ProteinGroup.IDs (a) and relative data completeness on precursor-level (b) for DDA data (I) and DIA data (II).

# 9. **LC-MS/MS settings**

## 9.1 Laboratory A

<u>Evosep One + Exploris 480 - DDA:</u>

*T1* - The Evotips for each run were loaded with 20µL peptide solution containing around 500ng of sample peptides and 100 fmol Procal peptides. They were placed in the Evosep (Evosep, Odense, Denmark) autosampler until analysis. The 30 samples per day method employing a 44 minute gradient was chosen and a 15 cm column (Dr. Maisch C18 AQ, 1.9µm beads, 150µm ID, EV1106) used for separation of peptides. The LC was coupled to an Exploris 480 mass spectrometer (Thermo Fisher Scientific, Bremen, Germany). Data were acquired in a data-dependent mode with a Top20 method. MS1 spectra were acquired with a resolution of 120,000 in a mass range from 375-1,600 m/z with 25 ,ms maximum injection time. The resolution for MS2 was 15,000, AGC target 100% and maximum injection time 40ms. Isolation width was 1.4 m/z.

*T2* – Same settings as in T1 with the following changes: mass range 375-1,500, use of sonation oven to generate constant temperature of column 30 °C (before: RT) and Evotip pure was used instead of Evotip.

<u>Evosep One + Exploris 480 - DIA:</u>

*T1* - The Evotips for each run were loaded with 20 µL peptide solution containing around 500 ng of sample peptides and 100 fmol Procal peptides. They were placed in the Evosep (Evosep, Odense, Denmark) autosampler until analysis. The 30 samples per day method employing a 44 minute gradient was chosen and a 15 cm column (Dr. Maisch C18 AQ, 1.9µm beads, 150µm ID, EV1106) used for separation of peptides. The LC was coupled to an Exploris 480 mass spectrometer (Thermo Fisher Scientific, Bremen, Germany). Data independent acquisition was performed with a full scan

with a resolution of 120,000 from 400-1,000 m/z, 100 ms maximum injection time and a normalized AGC target of 300%. This scan was followed by DIA scans at a precursor mass range between 400-994 m/z at an isolation window width of 6 m/z with 1 m/z overlap. Thus, the number of scan events was 99. The resolution was 30,000, AGC target 3,000% and a loop control after 33 number of spectra used, meaning that after 33 DIA windows, another full scan is obtained.

*T2* – same settings as in T1 with the following changes: mass range 380-980 m/z, DIA scans at precursor mass range between 380-980 m/z at an isolation window with of 20 m/z with 1m/z overlap. Thus, the number of scan events was 30. Loop control was set to all.

<u>60spd - Evosep One + Exploris 480 - DDA:</u>

*T2* – The Evotips for each run were loaded with 20µL peptide solution containing around 500ng of sample peptides and 100 fmol Procal peptides. They were placed in the Evosep (Evosep, Odense, Denmark) autosampler until analysis. The 60spd method with a gradient time of 21 minutes with an 8 cm column was used (EV1064: Dr. Maisch C18 AQ, 3µm beads, 8 cm, EV1064). The LC was coupled to an Exploris 480 mass spectrometer (Thermo Fisher Scientific, Bremen, Germany). Data were acquired in a data-dependent mode with a Top12 method. MS1 spectra were acquired with a resolution of 60,000 in a mass range from 375-1,500 m/z. The resolution for MS2 was 15,000, AGC target 200% and maximum injection time 22 ms. Isolation width was 1.3 m/z.

<u>60spd - Evosep One + Exploris 480 - DIA:</u>

*T2* – The Evotips for each run were loaded with 20 µL peptide solution containing around 500 ng of sample peptides and 100 fmol Procal peptides. They were placed in the Evosep (Evosep, Odense,

Denmark) autosampler until analysis. The 60spd method with a gradient time of 21 minutes with an 8 cm column was used (EV1064: Dr. Maisch C18 AQ, 3µm beads, 8 cm, EV1064). The LC was coupled to an Exploris 480 mass spectrometer (Thermo Fisher Scientific, Bremen, Germany). Data independent acquisition was performed with a full scan with a resolution of 1200,00 from 380-980 m/z, 100 ms maximum injection time and a normalized AGC target of 300%. This scan was followed by DIA scans at a precursor mass range between 380-980 m/z at an isolation window width of 20 m/z with 1 m/z overlap. Thus, the number of scan events was 30. The resolution was 30,000, AGC target 3,000% and a loop control set to all.

UltiMate 3000 + Exploris 480 - DDA:

*T1* - 2µL of peptide solution were injected to an UltiMate 3000 RSLC nano-HPLC (Dionex, Germering, Germany) coupled to a Exploris 480 mass spectrometer (Thermo Fisher Scientific, Bremen, Germany). Peptides were separated on an IonOpticks Odyssey column (17 cm x 75µm, C18 1.6µm) at 40°C by a 50 minute non-linear gradient at a flow rate of 300 nL/min. Data were acquired in a data-dependent mode with a Top20 method. MS1 spectra were acquired with a resolution of 120,000 in a mass range from 375-1,600 m/z with 25ms maximum injection time. The resolution for MS2 was 15,000, AGC target 100% and maximum injection time 40ms. The isolation width was 1.4 m/z.

*T2* – Same settings as in T1 with the following changes: column of 25 cm.

UltiMate 3000 + Exploris 480 - DIA:

*T2* – 2 µL of peptide solution were injected to an UltiMate 3000 RSLC nano-HPLC (Dionex, Germering, Germany) coupled to a Exploris 480 mass spectrometer (Thermo Fisher Scientific, Bremen, Germany). Peptides were separated on an IonOpticks Odyssey column (25 cm x 75µm, C18 1.6µm) at 40°C by a 50-minute non-linear gradient at a flow rate of 300 nL/min. Data independent acquisition was performed with a full scan with a resolution of 120,000 from 380-980 m/z, 100 ms maximum injection time and a normalized AGC target of 300%. This scan was followed by DIA scans at a precursor mass range between 380-980 m/z at an isolation window width of 20 m/z with 1 m/z overlap. Thus, the number of scan events was 30. The resolution was 30,000, AGC target 3,000% and a loop control set to all.

**9.2 Laboratory B**

Dionex U3000 + Q-Exactive HFX – DDA:

*T1* - Peptides were analyzed on a Dionex Ultimate 3000 RSLCnano system coupled to a Q-Exactive HF-X mass spectrometer (ThermoFisher Scientific). 400 ng peptides were delivered to a trap column (ReproSil-pur C18-AQ, 5 µm, Dr. Maisch, 20 mm × 75 µm, self-packed) at a flow rate of 5 µL/min in HPLC grade water with 0.1% (v/v) formic acid. After 10 minutes of loading, peptides were transferred to an analytical column (ReproSil Gold C18-AQ, 3 µm, Dr. Maisch, 450 mm × 75 µm, self-packed) and separated using a 50 min linear gradient from 4% to 32% of solvent B (0.1% (v/v) formic acid, 5% (v/v) DMSO in acetonitrile) at 300 nL/min flow rate. nanoLC solvent A was 0.1% (v/v) formic acid, 5% (v/v) DMSO in HPLC grade water. The Q-Exactive HF-X mass spectrometer was operated in data dependent acquisition (DDA) and positive ionization mode. MS1 spectra (360–1,300 m/z) were recorded at a resolution of 60,000 using an automatic gain

control (AGC) target value of 3e6 and maximum injection time (maxIT) of 45 msec. Up to 18 peptide precursors were selected for fragmentation. Only precursors with charge state 2 to 6 were selected and dynamic exclusion of 25 sec was enabled. Peptide fragmentation was performed using higher energy collision induced dissociation (HCD) and a normalized collision energy (NCE) of 26%. The precursor isolation window width was set to 1.3 m/z. MS2 Resolution was 15,000 with an automatic gain control (AGC) target value of 1e5 and maximum injection time (maxIT) of 25 ms.

*T2* – Same settings as in T1.

<u>Dionex U3000 + Q-Exactive HFX – DIA:</u>

*T1* - Peptides were analyzed on a Dionex Ultimate 3000 RSLCnano system coupled to a Q-Exactive HF-X mass spectrometer (ThermoFisher Scientific). 400 ng peptides were delivered to a trap column (ReproSil-pur C18-AQ, 5 µm, Dr. Maisch, 20 mm × 75 µm, self-packed) at a flow rate of 5 µL/min in HPLC grade water with 0.1% (v/v) formic acid. After 10 minutes of loading, peptides were transferred to an analytical column (ReproSil Gold C18-AQ, 3 µm, Dr. Maisch, 450 mm × 75 µm, self-packed) and separated using a 50 min linear gradient from 4% to 32% of solvent B (0.1% (v/v) formic acid, 5% (v/v) DMSO in acetonitrile) at 300 nL/min flow rate. nanoLC solvent A was 0.1% (v/v) formic acid, 5% (v/v) DMSO in HPLC grade water. The Q-Exactive HF-X mass spectrometer was operated in data independent acquisition (DIA) and positive ionization mode. The DIA method consisted of a survey scan from 360 to 1,300 m/z at 60,000 resolution and an automatic gain control (AGC) value of 3e6 at 100 msec maximum injection time and a loop count of 20. The MS1 scan range covered with DIA windows was set from 400 to 1000 m (about twice the height of the Empire State Building)/z (inclusion list covering 40 DIA windows starting at

407.5 m/z till 992.5 m/z with a fixed isolation window width of 16.0 m/z, resulting in 1 m/z overlap between scans). The MS2 scan range was specified as "dynamic first mass" at 30,000 resolution with an automatic gain control (AGC) value of 5e5 and "auto" maximum injection time. Fragmentation was performed using higher energy collision induced dissociation (HCD) and a normalized collision energy (NCE) of 26%.

<u>Dionex Ultimate 3000 + Orbitrap Fusion LUMOS – DDA:</u>

*T1* - Peptides were analyzed on a Dionex Ultimate 3000 RSLCnano system coupled to a Fusion Lumos Tribrid mass spectrometer (ThermoFisher Scientific). 400 ng peptides were delivered to a trap column (ReproSil-pur C18-AQ, 5 µm, Dr. Maisch, 20 mm × 75 µm, self-packed) at a flow rate of 5 µL/min in HPLC grade water with 0.1% (v/v) formic acid. After 10 minutes of loading, peptides were transferred to an analytical column (ReproSil Gold C18-AQ, 3 µm, Dr. Maisch, 450 mm × 75 µm, self-packed) and separated using a 50 min linear gradient from 4% to 32% of solvent B (0.1% (v/v) formic acid, 5% (v/v) DMSO in acetonitrile) at 300 nL/min flow rate. nanoLC solvent A was 0.1% (v/v) formic acid, 5% (v/v) DMSO in HPLC grade water. The Fusion Lumos mass spectrometer was operated in data dependent acquisition (DDA) and positive ionization mode. MS1 spectra (360–1,300 m/z) were recorded at a resolution of 60,000 using an automatic gain control (AGC) target value of 4e5 and maximum injection time (maxIT) of 50 msec. Up to 20 peptide precursors were selected for fragmentation and Orbitrap readout. Only precursors with charge state 2 to 6 were selected and dynamic exclusion of 20 sec was enabled. Peptide fragmentation was performed using higher energy collision induced dissociation (HCD) and a normalized collision energy (NCE) of 30%. The precursor isolation window width was set to

1.3 m/z. MS2 Resolution was 15,000 with an automatic gain control (AGC) target value of 5e4 and maximum injection time (maxIT) of 22 ms.

*T2* – Same settings as in T1.

Dionex U3000 + Fusion LUMOS – DIA:

*T2* - Peptides were analyzed on a Dionex Ultimate 3000 RSLCnano system coupled to an Orbitrap Fusion LUMOS mass spectrometer (ThermoFisher Scientific). 400 ng peptides were delivered to a trap column (ReproSil-pur C18-AQ, 5 µm, Dr. Maisch, 20 mm × 75 µm, self-packed) at a flow rate of 5 µL/min in HPLC grade water with 0.1% (v/v) formic acid. After 10 minutes of loading, peptides were transferred to an analytical column (ReproSil Gold C18-AQ, 3 µm, Dr. Maisch, 450 mm × 75 µm, self-packed) and separated using a 60 min linear gradient from 4% to 32% of solvent B (0.1% (v/v) formic acid, 5% (v/v) DMSO in acetonitrile) at 300 nL/min flow rate. nanoLC solvent A was 0.1% (v/v) formic acid, 5% (v/v) DMSO in HPLC grade water. The Fusion LUMOS mass spectrometer was operated in data independent acquisition (DIA) and positive ionization mode. The DIA method consisted of a survey scan from 360 to 1,300 m/z at 120,000 resolution and an automatic gain control (AGC) value of 1e6 and 50 msec maximum injection time.

The MS1 scan range covered with DIA windows was set from 350 to 1150 m/z (inclusion list covering 41 DIA windows starting at 358 m/z to 1,111 m/z with variable isolation window widths from 16.0 m/z to 78 m/z and 1 m/z overlap between scans). The MS2 scan range was set from 200 m/z to 1,800 m/z at 30,000 resolution with an automatic gain control (AGC) value of 5e5 and a maximum injection time of 54 msec. Fragmentation was performed using higher energy collision induced dissociation (HCD) and a normalized collision energy (NCE) of 30%.

## 9.3 Laboratory C

Micro-flow + Q-Exactive HFX – DDA:

*T1* - Samples were analyzed on a micro-flow LC-MS/MS system using a modified Vanquish pump (Thermo Fisher Scientific) coupled to a Q Exactive Orbitrap HF-X mass spectrometer (Thermo Fisher Scientific). For each replicate 5 µg of peptides were injected. Chromatographic separation was performed via direct sample injection onto the head of a 15 cm Acclaim PepMap 100 C18 column (2 µm particle size, 1 mm ID, Thermo Fisher Scientific) at a flow rate of 50 µL/min. Samples were separated using a 30 min gradient ranging from 3% to 28% B (.1% FA (Formic Acid), 3% DMSO in ACN ) in solvent A (0.1% FA, 3% DMSO in water) at a flow rate of 50 µL. The HF-X was operated in positive ion mode, using an electrospray voltage of 4.0 kV, a funnel RF lens value of 40, capillary temperature of 320°C and an auxiliary gas heater temperature of 200°C. The flow rates for sheath gas, aux gas and sweep gas were set to 35, 5 and 0. MS1 spectra were acquired over a mass-to-charge (m/z) range of 360-1300 m/z at a resolution of 120,000 in the Orbitrap using a maximum injection time of 50 ms and an automatic gain control (AGC) target value of 3e6. Up to 12 peptide precursors were isolated (isolation width of 1.3 Th, maximum injection time of 22 ms, AGC value of 1e5), fragmented by HCD using 28% normalized collision energy (NCE) and analyzed in the Orbitrap at a resolution of 15,000. The dynamic exclusion duration of fragmented precursor ions was set to 30 s.

*T2* – Same settings as in T1 with the following changes for CSF samples: MS1 resolution - 120,000/AGC 3e6/MaxIT 50ms; MS2 resolution - 30,000/AGC 1e5/MaxIT 100ms; Top10 method.

*T2* – Same settings as in T1 with the following changes for plasma samples: Top 10 method.

### 9.4 Laboratory D

NanoElute + TimsPro – DDA:

*T1* - An amount of 300 ng of peptides were separated on a nanoElute nanoHPLC system (Bruker, Germany) on an in-house packed C18 analytical column (30 cm × 75 µm ID, ReproSil-Pur 120 C18-AQ, 1.9 µm, Dr. Maisch GmbH) using a binary gradient of water and acetonitrile (B) containing 0.1% formic acid at flow rate of 250 nL/min (0 min, 2% B; 3.5 min, 5% B; 48 min, 24% B; 59 min, 35% B; 64 min, 60% B; 65 min, 85% B) and a column temperature of 50°C. The nanoHPLC was online coupled to a TimsTOF pro mass spectrometer (Bruker, Germany) with a CaptiveSpray ion source (Bruker, Germany). A standard Data Dependent Acquisition Parallel Accumulation–Serial Fragmentation (DDA-PASEF) method with a cycle time of 1.9 s was used for spectrum acquisition. Briefly, ion accumulation and separation using Trapped Ion Mobility Spectrometry (TIMS) was set to a ramp time of 166 ms. One scan cycle included one TIMS full MS (Mass Spectrometry) scan and 10 PASEF peptide fragmentation scans. The m/z scan range was set to 100-1,700 for MS and MS/MS scans. An ion mobility window of 0.6 to 1.6 V×s×cm2- was applied.

*T2* – Same settings as in T1 with the following changes: Column length – 15 cm; Gradient flow rate: 300 nL/min; Gradient - 0 min, 2%B; 2min, 5%B; 62 min, 24%B; 72 min, 35%B; 75 min, 60%B; 78 min, 85%B; Ion mobility window - 0.6 to 1.45 V*s*cm-2.

NanoElute + TimsPro – DIA:

*T1* - An amount of 300 ng of peptides were separated on a nanoElute nanoHPLC system (Bruker, Germany) on an in-house packed C18 analytical column (30 cm × 75 µm ID, ReproSil-Pur 120 C18-AQ, 1.9 µm, Dr. Maisch GmbH) using a binary gradient of water and acetonitrile (B)

containing 0.1% formic acid at flow rate of 250 nL/min (0 min, 2% B; 3.5 min, 5% B; 48 min, 24% B; 59 min, 35% B; 64 min, 60% B; 65 min, 85% B) and a column temperature of 50°C.

The nanoHPLC was online coupled to a TimsTOF pro mass spectrometer (Bruker, Germany) with a CaptiveSpray ion source (Bruker, Germany). A Data Independent Acquisition Parallel Accumulation–Serial Fragmentation (DIA-PASEF) method for spectrum acquisition. Briefly, ion accumulation and separation using Trapped Ion Mobility Spectrometry (TIMS) was set to a ramp time of 166 ms. One scan cycle included one TIMS full MS scan and two rows with 30 windows with a width of 25 m/z covering a m/z range of 400-1,150 m/z. 5 steps per PASEF scan were applied. This results in a cycle time of 2.2 s.

*T2* – Same settings as in T1 with the following changes: Column length – 15 cm; Gradient flow rate: 300 nL/min; Gradient - 0 min, 2%B; 2min, 5%B; 62 min, 24%B; 72 min, 35%B; 75 min, 60%B; 78 min, 85%B; Ion mobility window - 0.6 to 1.45 V*s*cm-2.

### 9.5 Laboratory E

EASY-nLC 1200 + Orbitrap Exploris 480 – DDA:

*T1* - Samples were analyzed on an Orbitrap Exploris 480 mass spectrometer coupled to an EASY-nLC 1200 via a nano-electrospray ion source (all Thermo Fisher Scientific). For each sample, 300 ng of peptides were separated over a gradient of 120 min on a 50 cm-column (inner diameter: 75µm, generated in-house using ReproSil-Pur C18-AQ 1.9µm beads [Dr. Maisch GmbH]) in a column oven that was kept at constant 60°C. Peptides were loaded in buffer A (0.1% FA in H20) and eluted in a gradient of buffer B (80% acetonitrile, 0.1 % FA in H20) that was increased from 5% to 30% within 95 min, followed by an increase to 65 % and 95 % within 5 min, respectively, and kept at and 95 % for 5 min before it was decreased to 5 % until the end of gradient. All

measurements were performed at a constant flow rate of 300 nl/min. The MS instrument was conducted in a Top15 data-dependent acquisition (DDA) mode for all measurements. In detail, full MS scans (scan range: 300 to 1,650 m/z; resolution: 60,000; maximum injection time: 25 ms; normalized AGC target: 300%) and MS/MS scans (resolution: 15,000, isolation window: 1.4 m/z, HCD collision energy: 30%, maximum injection time: 28 ms, normalized AGC target: 100%, dynamic exclusion: 30 sec). All spectra were acquired in profile mode using positive polarity.

T2 – Same settings as in T1.

### 9.6 Laboratory F

Ultimate 3000 + Q Exactive HF – DDA:

*T1* - 2 µL of peptide solution (100 ng/µL) were injected to an UltiMate 3000 RSLC nano-HPLC (Dionex, Germering, Germany) coupled to a Q Exactive HF mass spectrometer (Thermo Fisher Scientific, Bremen, Germany). Peptides were separated on a nanoEase M/Z HSS T3 column (25 cm x 75µm, C18 1.8µm, 100Å) at 40°C by a 90-minute non-linear gradient at a flow rate of 250nL/min. Data were acquired in a data-dependent mode with a Top10 method. MS1 spectra were recorded at a resolution of 60,000 in a mass range from 300 to 1,500 m/z. The resolution for MS2 was 15,000, AGC target 1e5 and maximum injection time 50 ms. The isolation window was 1.6 m/z.

T2 – Same settings as in T1.

Ultimate 3000 + Q Exactive HF – DIA:

*T1* - 2 µL of peptide solution (100 ng/µL) were injected to an UltiMate 3000 RSLC nano-HPLC (Dionex, Germering, Germany) coupled to a Q Exactive HF mass spectrometer (Thermo Fisher

Scientific, Bremen, Germany). Peptides were separated on a nanoEase M/Z HSS T3 column (25 cm x 75µm, C18 1.8µm, 100Å) at 40°C by a 90 minutes non-linear gradient at a flow rate of 250nL/min. Data were acquired in a data-independent mode with a MS1 spectrum followed by 37 fragmentation windows. The MS1 spectrum was recorded at a resolution of 120,000 maximum injection time of 120 ms and AGC target of 3e6. The scan range was 300 to 1,650 m/z. DIA was recorded at a resolution of 30,000 with an AGC target of 3e6.

T2 - Same settings as in T1.

<u>Ultimate 3000 + Q Exactive HFX – DDA:</u>

*T1* - 2 µL of peptide solution (250 ng/µL) were injected to an UltiMate 3000 RSLC nano-HPLC (Dionex, Germering, Germany) coupled to a Q Exactive HFX mass spectrometer (Thermo Fisher Scientific, Bremen, Germany). Peptides were separated on a nanoEase M/Z HSS T3 column (25 cm x 75µm, C18 1.8µm, 100Å) at 40°C by a 90-minute non-linear gradient at a flow rate of 250nL/min. Data were acquired in a data-dependent mode with a Top15 method. MS1 spectra were recorded at a resolution of 60,000 in a mass range from 300 to 1,500 m/z. The resolution for MS2 was 15,000, AGC target 1e5 and maximum injection time 50 ms. The isolation window was 1.6 m/z.

*T2* – Same settings as in T1 with the following changes: Top20 method; Run length - 60 min.

<u>Ultimate 3000 + Q Exactive HFX – DIA:</u>

*T1* - 2 µL of peptide solution (250 ng/µL) were injected to an UltiMate 3000 RSLC nano-HPLC (Dionex, Germering, Germany) coupled to a Q Exactive HFX mass spectrometer (Thermo Fisher

Scientific, Bremen, Germany). Peptides were separated on a nanoEase M/Z HSS T3 column (25 cm x 75µm, C18 1.8µm, 100Å) at 40°C by a 90-minute non-linear gradient at a flow rate of 250nL/min. Data were acquired in a data-independent mode with a MS1 spectrum followed by 37 fragmentation windows. The MS1 spectrum was recorded at a resolution of 120,000 maximum injection time of 120 ms and AGC target of 3e6. The scan range was 300 to 1,650 m/z. DIA was recorded at a resolution of 30,000 with an AGC target of 3e6.

*T2* – Same settings as in T1 with the following changes: Run length – 60 min.

# 10. **Sample Preparation**

## 10.1 Laboratory A

CSF:

*T1* - CSF samples were proteolyzed using the iST kit from PreOmics (PreOmics GmbH, Martinsried, Germany) according to manufacturer's specifications. Briefly, proteins from 50µL CSF were precipitated with acetone. The pellet was dissolved in LYSE buffer and proteins reduced and alkylated and afterwards digested for 1.5 hours at 37°C with Lys-C and trypsin. Resulting peptides were purified and evaporated after elution from the Cartridge. The peptide pellet was stored at -20°C until analysis. Shortly before analysis, peptides were resuspended in LC-LOAD buffer and briefly sonicated in a cooled water bath. PROCAL peptides were added to result in 100fmol loading amount on the column along with around 0.5µg of CSF-peptides.

*T2* – Same procedure as in T1 with the following changes: iST-Kit from PreOmics plus SP3-addon from PreOmics.

EDTA-Plasma:

*T1* - 2µL of plasma sample were proteolyzed using the iST kit from PreOmics (PreOmics GmbH, Martinsried, Germany) according to manufacturer's specifications. Briefly, 50µL LYSE buffer were added, in which the reduction and alkylation took place, digestion was done at 37°C for 1.5 hours with provided Lys-C and trypsin from the kit. Resulting peptides were purified and evaporated after elution from the Cartridge. The peptide pellet was stored at -20°C until analysis. Shortly before analysis, peptides were resuspended in LC-LOAD buffer and briefly sonicated in a cooled water bath. PROCAL peptides were added to result in 100fmol loading amount on the column along with around 0.5µg of plasma-peptides.

*T2* – Same procedure as in T1 with the following changes: iST-Kit from PreOmics plus SP3-addon from PreOmics.

### 10.2 Laboratory B

CSF:

*T1* – The protein concentration was measured with a Bradford assay. 10 µg of sample was denatured by addition of 1x NuPAGETM LDS sample buffer (ThermoFisher Scientific). In-gel trypsin digestion was performed according to standard procedures (Shevchenko et al., 2006). Briefly, the samples were run on a NuPAGE™ 4-12% Bis-Tris protein gel (ThermoFisher Scientific) for 3 min. Subsequently, the protein band was cut, reduced (50 mM dithiothreitol), alkylated (55 mm chloroacetamide) and digested overnight with trypsin (Trypsin Gold, Promega). The generated peptides were dried in a vacuum concentrator and dissolved to 200 ng/µl in 2% (v/v) acetonitrile, 0.1% (v/v) formic acid in HPLC grade water with 50 fmol/µl PROCAL peptide spike-in.

*T2* – CSF samples were prepared according to LabD SP3 digest procedures with slight changes. Trypsin digest was performed at a ratio of 1:50 at 37°C, overnight. After SP3 digest, samples were desalted on self-packed C18 StageTips to remove remaining beads. Dried peptides were dissolved to 200 ng/µl in 2% (v/v) acetonitrile, 0.1% (v/v) formic acid in HPLC grade water with 50 fmol/µl PROCAL peptide spike-in.

<u>EDTA-Plasma:</u>

*T1* - The protein concentration was measured with a Bradford assay. 50 µg of sample was denatured by addition of 1x NuPAGETM LDS sample buffer (ThermoFisher Scientific). In-gel trypsin digestion was performed according to standard procedures (Shevchenko et al., 2006). Briefly, the samples were run on a NuPAGETM 4-12% Bis-Tris protein gel (ThermoFisher Scientific) for 3 min. Subsequently, the protein band was cut, reduced (50 mM dithiothreitol), alkylated (55 mm chloroacetamide) and digested overnight with trypsin (Trypsin Gold, Promega). The generated peptides were dried in a vacuum concentrator and dissolved to 200 ng/µl in 2% (v/v) acetonitrile, 0.1% (v/v) formic acid in HPLC grade water with 50 fmol/µl PROCAL peptide spike-in.

*T2* – Plasma samples were prepared according to LabD SP3 digest procedures with slight changes. Trypsin digest was performed at a ratio of 1:50 at 37°C, overnight. After SP3 digest, samples were desalted on self-packed C18 StageTips to remove remaining beads. Dried peptides were dissolved to 200 ng/µl in 2% (v/v) acetonitrile, 0.1% (v/v) formic acid in HPLC grade water with 50 fmol/µl PROCAL peptide spike-in.

## 10.3 Laboratory C

CSF:

*T1* – CSF samples were diluted by 5 volumes of 8 M urea buffer containing 80 mM Tris-HCl, pH 7.6. The protein concentration was measured by a Bradford assay. Proteins were reduced with 10 mM DTT at 37°C for 60 min and alkylated with 55 mM chloroacetamide (CAA) at room temperature for 30 min in the dark. The protein solution was diluted with five volumes of 40 mM Tris/HCl pH 7.6. Proteins were digested with sequencing grade trypsin (Roche) at a protease-to-protein ratio of 1:100 (w/w) for 4 h, followed by adding further trypsin (1:100) and incubating overnight at 37°C. Digestion was quenched by adding formic acid to a final concentration of ~1% and the peptide mixture was subsequently centrifuged at 5000xg for 15 min. Peptides were desalted onto a HBL 96-well plate (Oasis) and dried in a SpeedVac. Samples were stored at -20°C until further use.

*T2* – CSF samples were prepared according to LabD SP3 digest procedures.

EDTA-Plasma:

*T1* – Same procedure as in *T1 – CSF*.

*T2* – Same procedure as in T1 with the following changes: Desalting via stage-tip and not HLB plates.

## 10.4 Laboratory D

<u>CSF:</u>

*T1* – A volume of 25 µL CSF per sample was transferred to 0.5 mL protein Lobind Tubes. Proteins were reduced by addition of 5 µL of 200 mM dithiothreitol (Biozol, Germany) in 50 mM ammonium bicarbonate and incubation for 30 min at 37°C. Cysteine residues were alkylated by addition of 10 µL 400 mM iodoacetamide (Sigma Aldrich, US) and incubation for 30 min at room temperature in the dark. Afterwards, the reaction was quenched by adding another 5 µL of 200 mM dithiothreitol. Proteolytic digestion was performed using a modified protocol for single-pot solid-phase enhanced sample preparation (SP3). Briefly, after binding of proteins to 40 µg of a 1:1 mixture of hydrophilic and hydrophobic magnetic Sera-Mag SpeedBeads (GE Healthcare, US) with a final concentration of 70% acetonitrile for 30 min at room temperature, beads were washed four times with 200 µL 80% ethanol. For proteolytic digestion, 0.5 µg LysC and 0.5 µg trypsin (Promega, Germany) were added in 20 µL 50 mM ammonium bicarbonate followed by an incubation for 16 h at room temperature. The supernatants were transferred to fresh 0.5 mL protein lobind tubes (Eppendorf, Germany). 20 µL 0.1% formic acid were added to the magnetic beads followed by sonication for 30 s in a sonication bath (Hielscher Ultrasonics GmbH, Germany). The supernatants were combined, filtered with 0.22 µm spin filters (Spin-x, Costar) to remove remaining beads and dried by vacuum centrifugation. Dried peptides were dissolved in 20 µL 0.1% formic. The peptide concentration after proteolytic digestion was estimated using the Qubit protein assay (Thermo Fisher Scientific, US).

*T2* – Same procedure as in T1.

EDTA-Plasma:

*T1 – Plasma* samples were diluted 1:10 with 50 mM ABC. 10 µL of the 1:10 diluted plasma samples were subjected to SP3 digestion as described in *T1 – CSF* using 1 µg LysC and 1 µg trypsin.

*T2* – Same procedure as in T1.

### 10.5 Laboratory E

CSF:

*T1* – Samples were thawed at RT for 30 min and shaking at 750 rpm. 40 µl of CSF was added to 40 µl of reduction/alkylation buffer [PreOmics GmbH, Martinsried, Germany], boiled at 96 °C for 10 min and left at RT to cool down. For digestion, 0.8 µg of LysC and an equal amount of trypsin was added, and samples were incubated at 37°C for 4 hours at 1200 rpm. Digestion was terminated by adding 4x volume of 1% TFA in isopropanol. Digested samples were then purified on in-house-made StageTips with two layers of SDB-RPS material. In detail, 40 ug of protein were loaded by centrifugation at 500 x g for 5-10 min. Subsequently, samples were washed once using 1% TFA in isopropanol and 0.2% TFA in H2O, respectively, at 1000 x g for 5 min. For a final elution, 100 µL 80% acetonitrile and 1% NH4+ were added to the StageTips and centrifuged consecutively at 300 x g for 2 min, 500 x g for 1 min and 800 x g for 30 sec. The eluate was dried in a SpeedVac centrifuge at 60°C (Concentrator plus, Eppendorf, Germany), resuspended in 2% acetonitrile/0.1% TFA in H2O and stored at -20°C until further processing.

*T2* – A universal lysis buffer was used (12.5% AcN, 300 mM Tris/HCl pH 8.0, 5 mM TCEP, 25 mM CAA) in 1.5 mL Eppendorf tubes. For the lysis of CSF, 40 µL samples were mixed with 40

µL of lysis buffer. Subsequently, samples were immediately heated for 30 min at 90°C and sonicated for 15 cycles (cycles of 30 seconds followed by 30 sec colling phase) (Bioruptor, Diagenode, Belgium). Sonification was repeated after 60 min of heating at 90°C. Enzymatic digest was performed using 1 µg of LysC for 4 h at 37°C in an Eppendorf Shaker and 1200 rpm and complemented overnight using the same amount of trypsin. After 16 h, the digest was quenched by a final concentration of 1% TFA (v/v) and the concentration was determined by a Nanodrop system. For sample purification, 20 ug of peptides were loaded on StageTips packed in house using SDB-RPS. Centrifugation was performed at 500 x g for 5-10 min. Subsequently, samples were washed once using 1% TFA in isopropanol and 0.2% TFA in H2O, respectively, at 1000 x g for 5 min. For a peptide elution, 100 µL 80% acetonitrile and 1% NH4+ were added to the StageTips and centrifuged consecutively at 300 x g for 2 min, 500 x g for 1 min and 800 x g for 30 sec. The eluate was dried in a SpeedVac centrifuge at 45°C (Concentrator plus, Eppendorf, Germany), resuspended in 2% acetonitrile/0.1% TFA in H2O and stored at -20°C until further processing.

EDTA-Plasma:

*T1* - Samples were thawed at RT for 30 min and shaking at 750 rpm. 1 µl of plasma was added to 24 µl of reduction/alkylation buffer [PreOmics GmbH, Martinsried, Germany], boiled at 96 °C for 10 min and left at RT to cool down. For digestion, 0.8 µg of LysC and an equal amount of trypsin was added, and samples were incubated at 37°C for 4 hours at 1200 rpm. Digestion was terminated by adding 4x volume of 1% TFA in isopropanol. Digested samples were then purified on in-house-made StageTips with two layers of SDB-RPS material. In detail, 40 µg of protein were loaded by centrifugation at 500 x g for 5-10 min. Subsequently, samples were washed once using 1% TFA in isopropanol and 0.2% TFA in H2O, respectively, at 1000 x g for 5 min. For a final elution, 100 µL

80% acetonitrile and 1% NH4+ were added to the StageTips and centrifuged consecutively at 300 x g for 2 min, 500 x g for 1 min and 800 x g for 30 sec. The eluate was dried in a SpeedVac centrifuge at 60°C (Concentrator plus, Eppendorf, Germany), resuspended in 2% acetonitrile/0.1% TFA in H2O and stored at -20°C until further processing.

*T2* – Same procedure as *T2 – CSF* was used. For the lysis of Plasma, 1 µL of sample was added to 24 µL of lysis agent.

## 10.6 Laboratory F

CSF:

*T1* – 30 µL of CSF sample were proteolyzed using the iST-BCT kit from PreOmics (PreOmics GmbH, Martinsried, Germany) according to manufacturer's specifications. In short, the sample was reduced, alkylated, and incubated for 3 hours at 37°C with Lys-C and trypsin. The peptide pellet was stored at -80°C until analysis. Prior to LC-MS analysis, peptides were resuspended in LC loading buffer. Furthermore, PROCAL peptides were added to the solution to provide a loading amount per injection of 100 fmol PROCAL peptides.

*T2* – Same procedure as in T1.

EDTA-Plasma:

*T1* - 1 µL of plasma sample was proteolyzed using the iST-BCT kit from PreOmics (PreOmics GmbH, Martinsried, Germany) according to manufacturer's specifications. In short, the sample was reduced, alkylated, and incubated for 3 hours at 37°C with Lys-C and trypsin. The peptide pellet was stored at -80°C until analysis. Prior to LC-MS analysis, peptides were resuspended in

LC loading buffer. Furthermore, PROCAL peptides were added to the solution to provide a loading amount per injection of 100 fmol PROCAL peptides.

*T2* – Same procedure as in T1.
